# Platelet-Rich Plasma and Platelet-Rich Fibrin in Endodontics: A Scoping Review

**DOI:** 10.3390/ijms26125479

**Published:** 2025-06-07

**Authors:** Simão Rebimbas Guerreiro, Carlos Miguel Marto, Anabela Paula, Joana Rita de Azevedo Pereira, Eunice Carrilho, Manuel Marques-Ferreira, Siri Vicente Paulo

**Affiliations:** 1 Institute of Endodontics and Laboratory of Evidence-Based and Precision Dentistry, Faculty of Medicine, University of Coimbra, 3000-075 Coimbra, Portugal; joanarapereira.md@gmail.com (J.R.d.A.P.); mmferreira@fmed.uc.pt (M.M.-F.); sirivicentepaulo@gmail.com (S.V.P.); 2 Institute of Experimental Pathology, Faculty of Medicine, University of Coimbra, 3000-548 Coimbra, Portugal; 3 Institute of Integrated Clinical Practice and Laboratory of Evidence-Based and Precision Dentistry, Faculty of Medicine, University of Coimbra, 3000-075 Coimbra, Portugal; anabelabppaula@sapo.pt (A.P.); ecarrilho@fmed.uc.pt (E.C.); 4 Coimbra Institute for Clinical and Biomedical Research (iCBR), Area of Environment, Genetics and Oncobiology (CIMAGO), Faculty of Medicine, University of Coimbra, 3000-548 Coimbra, Portugal; 5 Centre for Innovate Biomedicine and Biotechnology (CIBB), University of Coimbra, 3000-548 Coimbra, Portugal; 6 Clinical Academic Center of Coimbra (CACC), 3004-561 Coimbra, Portugal; 7 Centre for Mechanical Engineering, Materials and Processes (CEMMPRE), Advanced Production and Intelligent Systems (ARISE), University of Coimbra, 3030-788 Coimbra, Portugal

**Keywords:** endodontics, platelet-rich fibrin, platelet-rich plasma, scoping review

## Abstract

Conventional endodontic treatment has several disadvantages, which lead to the introduction of regenerative endodontic procedures aiming to maintain tooth vitality. Platelet concentrates possess relevant biological properties, and their application has been explored in various endodontic procedures. The aim of this scoping review is to identify the applications of platelet-rich plasma (PRP) and platelet-rich fibrin (PRF) in endodontics. To identify and map the types of studies, the protocols for obtaining PRF/PRP, the most productive authors, and the journals where most articles were published on this topic until 2023. A literature search was performed in four databases (Medline, Embase, Cochrane Library, and Web of Science) until 20 December 2023. From the included articles, the following information was extracted: first author and publication year, endodontic procedure, platelet concentrate used, type of study, and journal of publication. A sampling methodology was adopted, and the five most recent articles for each procedure were used for additional information extraction: sample size and characteristics, pulp and periapical diagnosis, study protocol, platelet substrate and protocol for its obtention, treatment outcome, and follow-up. After selection, 412 articles were included. As for the type of endodontic procedure, regeneration procedures of immature teeth were the most reported, followed by apical surgery and pulpotomy and pulp protection. It was concluded that PRF is the most reported platelet concentrate. Regenerative procedures in immature teeth are the most described endodontic procedure. The success rate of PRF and PRP use is comparable to or even higher than that of conventional procedures and materials. However, there is significant heterogeneity in the protocols used for obtaining PRF and PRP and their clinical application.

## 1. Introduction

Endodontic pathologies are widely prevalent, and although conventional root canal therapy presents high success rates, it is associated with some disadvantages, such as loss of vitality and tooth structure [1]. This way, the preservation and regeneration of the pulpal–dentinal complex is highly desirable to maintain the teeth’ biological and mechanical functions. Tissue engineering procedures can be defined as regenerative procedures that allow a damaged or absent tissue to be replaced by a biological one, restoring its lost functions. To achieve such a result, a combination of stem cells, scaffolds, and a suitable microenvironment is used [2]. When applied to regenerative endodontics (RET), the aim is to regenerate the pulp–dentin complex tissue and the root structures, restoring the lost physiologic functions [3].

Besides biomaterials used in conventional endodontic treatment, such as calcium hydroxide, mineral trioxide aggregate (MTA), and calcium silicate materials, autologous platelet concentrates are being used with promising results. Platelet concentrates have been used in the past in several medical fields [4]. Among them, platelet-rich plasma and platelet-rich fibrin have been widely explored due to their ability to promote healing, tissue regeneration, and immunomodulatory properties [5,6]. These concentrates are obtained through centrifugation of blood and collection of the elements that enhance tissue regeneration, namely platelets, due to their growth factors, fibrin, which acts as a supporting matrix, and leukocytes due to their role in self-regulation of inflammatory and infectious phenomena [6].

The PRP preparation was first described by Whitman et al. in 1997; however, as the consistency of his product was gelatinous, it was labelled platelet gel [6,7,8]. According to the review by Umakanth et al., the term PRP defined by Kingsley in 1998 when performing experiments on blood coagulation [8]. In 2009, Dohan et al. classified PRP as P-PRP (poor leukocyte or pure PRP) and L-PRP (leukocyte PRP) [9]. The main difference between the two is the leukocyte concentration. Also, in the P-PRF protocol, a synthetic anticoagulant material and a gelling agent are needed [10].

In 2000, Choukroun and collaborators described the preparation protocol and application of a new autologous platelet concentrate, named Choukroun’s platelet-rich-fibrin, and branded it as a “second generation” platelet concentrate [11]. Choukrouns’ PRF was obtained by centrifuging collected blood with no anticoagulant addition at 3000 rpm for 10 min. As time passed, the original protocol developed by Choukroun et al. was modified to 2700 rpm for 12 min, as it gives a better polymerization of the fibrin clot and, therefore, stronger membranes [6]. Nowadays, this type of PRF preparation has become known as leukocyte and platelet-rich fibrin (L-PRF) due to the high concentration of leukocytes trapped in the fibrin clot [10,12]. As previously referred to, L-PRF was the first of the second-generation autologous platelet concentrates to be described, and it is one of the most used concentrates today [9].

Since the first time they were described, the classification, definition, and preparation protocols of platelet concentrates in the literature have been reported to have several variations. Consequently, the authors report a lack of uniformization, which limits the reproducibility of the concentrates’ obtention protocols and their applicability in everyday clinical practice [9].

More recently, the variants and subtypes of platelet concentrates have grown, mainly based on different centrifugation protocols. Such variants include the advanced-PRF (A-PRF) [13], the concentrated growth factor (CGF) [14], the injectable-PRF (I-PRF) [11], and titanium-PRF (T-PRF) [11], among others [11].

In the endodontic field, as in other dentistry fields, platelet concentrate use has been increasing for several indications, replacing or combining with existing materials. The use of PRF and PRP has been reported for procedures such as pulpotomy and as a pulp capping agent [15]; to promote the apexification and revascularization of immature teeth [16]; in apical surgery, combined with bone graft materials [17]; in tooth reimplantation of avulsed teeth [18] or in intentional reimplantation [19]; or in the management of root resorptions and/or root fractures [20].

Although PRP and PRF are being extensively used in various endodontic procedures, there is a lack of uniformization and detail when describing the preparation protocols, the type of concentrate used, and its application. This limits an appropriate evaluation of the protocols, treatment outcomes, and indications of such treatments. To surpass such limitations, a scoping review was planned and conducted to map the existing information on this topic published until 2023, as well as to identify any knowledge gap that should be addressed.

## 2. Results

### 2.1. Study Selection

**Figure 1:**
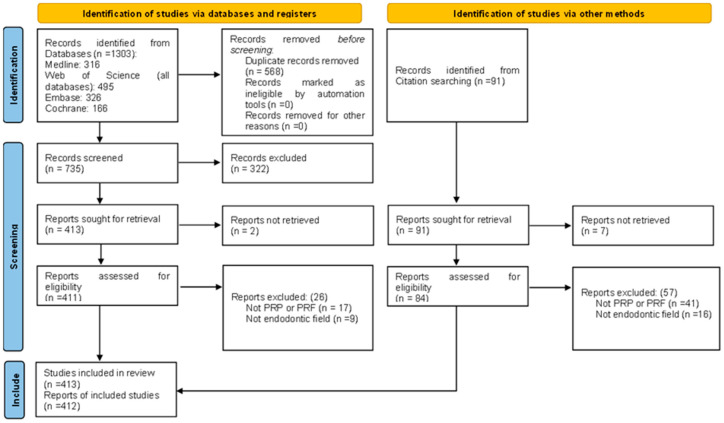
PRISMA flow diagram of the identification and selection of the results.

After the literature search process, a total of 1303 records were retrieved. The duplicate removal resulted in the exclusion of 568 articles. After, 735 records were screened by title and abstract, resulting in 413 that were sought for full-text retrieval. Of those, two could not be retrieved. Of the 411 records, after the screening phase by full text, 385 articles met the inclusion criteria and were included in this review. Furthermore, the references of the 385 included articles were searched for pertinent data, resulting in the inclusion of more than 27 studies, making 412 the number of articles included in this review (Figure 1).

### 2.2. Characteristics of the Included Studies

To map the information included in this review, a quantitative analysis was made of the following topics: type of platelet concentrate, study type, type of procedure, top 10 first-authors of included publications, top 10 journals where the included articles were published, and the number of articles published on this topic per year.

**Figure 2:**
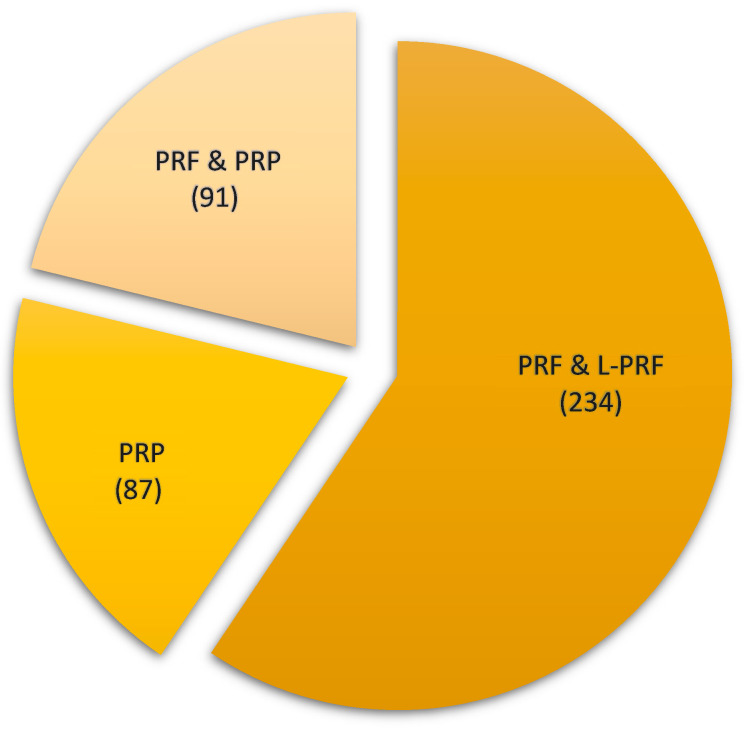
Type of platelet concentrate and respective number of reports. PRF: Platelet-rich fibrin; PRP: Platelet-rich plasma; L-PRF: Leukocyte and platelet-rich fibrin.

Regarding the type of platelet concentrate, PRF and L-PRF were the most described. Figure 2 presents the number of reports of each concentrate type.

Regarding the study type, case reports (*n* = 93), randomized controlled trials (RCTs) (*n* = 62), and animal studies (n = 34) were the most frequent original research reported. For review reports, narrative reviews were the most frequent ones (*n* = 66). Fifty-two randomized clinical trial protocols were also retrieved. The total of study types was 413, more than the records that were included in this review, 412. This can be explained by the fact that one study was categorized as both in vivo and in vitro. A detailed list of study types and respective number of reports is shown in Table 1 and Table S1. A complete list of the journals where the articles were published is presented in Table S2.

When evaluating the type of endodontic procedures described, most included articles (n = 185) address immature teeth regenerative procedures, mainly reporting revascularization/revitalization procedures, followed by apical surgery procedures (n = 80) where PRF and PRP are used as scaffolds in bone defects or for retro-obturation procedures, combined with bone graft materials. A complete list of endodontic procedures where PRF and PRP are being used, and the respective number of reports is shown in Table 1.

The most prolific authors on the topic were also identified and are presented in Table 1. The journals where most studies were published were also mapped. As presented in Table 1, journals within the endodontics and restorative dentistry scope were the most frequently included.

Finally, as shown in the table, an increase in the number of published studies on this topic was seen after 2011, with the last years presenting a larger number of published articles per year.

### 2.3. Endodontic Procedures

As stated in the Materials and Methods, to complement the information extracted from all included articles, a sampling methodology was also adopted to extract more in-depth information from the records collected. For that, the studies were first divided into the type of endodontic procedure performed, and the five most recent ones for each procedure were selected. The extracted information is presented in Tables S3–S15.

#### 2.3.1. Pulpotomy and Pulp Capping

Of the 412 included studies, 56 reported the use of PRP and PRF for pulpotomy and/or pulp capping procedures (Table 1). Of those, 44 describe the use of PRF, 4 describe the use of both PRF and PRP, and 8 describe the use of PRP. The sample was composed of 15 RCT protocols, 13 RCTs, 8 animal studies, 6 narrative reviews, 5 case series, 3 systematic reviews, 3 case reports, 1 in vitro study, 1 cohort, and 1 case-control study. Among the studies, both anterior and posterior teeth were reported to be submitted to this procedure. Moreover, among the included studies, some report the use of PRF/PRP in mature teeth and in the management of irreversible pulpitis [21].

The five more recent articles describing the use of PRP and PRF for pulpotomy or pulp capping procedures include one case report of a pulpotomy using PRF [22], two RCT protocols of studies aimed to evaluate the success of PRF in pulpotomy [23,24], and two RCT, one where the effectiveness of PRF was compared to nano-hydroxyapatite and MTA in pulpotomy of immature molars [15], and another where the effectiveness of PRF was compared to TheraCal or MTA as a pulpotomy agent [21]. Table S3 presents the extracted information from each one.

The studies mostly report the use of PRF for pulpotomy combined with pulp capping procedures. Interestingly, although some studies report and consider the use of platelet concentrates in vital pulp treatments, other studies include irreversible pulpitis situations. The PRF use was compared in most situations to the gold-standard pulp capping material Biodentine (Septodont, Saint-Maur-des-Fossés, France). The treatment success can be evaluated both by clinical (for instance, pain, tooth sensitivity), radiological methods (presence of apical lesion, presence of root resorption), or even histological methods (in animal studies). Regarding the obtained results, in the case report described by Mandviwala et al., root resorption was observed after the use of PRF combined with Biodentine (Septodont, Saint-Maur-des-Fossés, France), and conventional endodontic treatment needed to be performed [22]. In the RCT by Eid et al. comparing PRF with MTA and with nano-hydroxyapatite, the three groups presented apical lengthening and closure; however, a higher tendency to cause pulp obliteration was observed in the MTA and nano-hydroxyapatite groups compared to the PRF one [15]. Mohamed et al. compared PRF usage in pulpotomy with MTA and TheraCal (Bisco, Schaumburg, IL, USA) in irreversible and reversible pulpitis, showing the regaining of sensibility on 15 out of 20 teeth treated with PRF plus MTA [21]. The sampled studies also described the PRF application in different forms, such as membranes rather than fibrin gel/clot [15,21,24].

#### 2.3.2. Regenerative Endodontic Procedures in Immature Teeth

Of the 412 included reports, 185 described the use of PRF and/or PRP as a scaffold for regenerative endodontic procedures in immature teeth (Table 1). Of those, 73 report the use of PRF, 65 report the use of both PRF and PRP, and 47 report the use of PRP. Regarding the study type, 50 are narrative reviews, 38 are case reports, 23 are RCTs, 23 are systematic reviews, 15 are RCTs protocols, 15 are animal studies, 11 are case series, 4 are cohort studies, 2 are case-control studies, 2 are scoping reviews, and 2 is an umbrella review. In the referred studies, anterior immature teeth, mostly incisors, were the most reported teeth to be submitted to this type of procedure.

As shown in Table S4, the five more recent articles on this procedure include one RCT comparing the application of PRF and blood clot revascularization in immature teeth [25], two case reports describing the use of PRF in the revascularization of necrotic and irreversible pulpitis teeth [26,27], one systematic review of RCTs evaluating the revascularization of immature teeth using PRF, PRP and blood clot [28], and one case-control study comparing revascularization of necrotic immature teeth using concentrated growth factors or PRF [29]. All studies reported a significant increase in root length and dentin thickness and in apical response scores (periapical healing) in the PRF groups. The results of the original studies were supported by the systematic review performed by Rios-Osorio et al., which concluded that both PRP and PRF promote better root length growth, apical closure, and increased periapical bone density than BC [28]. As a limitation of the treatment, Kumar et al. reported a case where pulp canal obliteration was seen after PRF was used in revascularization [27]. Although two studies did not report the scaffold preparation protocol [27,28], the remaining followed the standard protocol of 3000 rpm for 10 min [25,29] or 2700 rpm for 12 min [26].

#### 2.3.3. Apexification

Of the 412 studies that reported the use of PRF and/or PRP in endodontic procedures, 20 reported its use in apexification procedures (Table 1). Of those, 16 used PRF, and 4 used PRF and PRP. The included records comprise five case series, six case reports, four systematic reviews, two RCTs, one animal study, one narrative review, and one RCT protocol. Among those studies, anterior teeth were the most frequent teeth being treated with platelet concentrates.

The sample of the five most recent studies in apexification includes two case series [30,31], two systematic reviews [32,33], and one RCT [34]. Detailed information on each study is presented in Table S5.

Biradar et al. [30] and Pruthi et al. [31] both used PRF plugs as a matrix to promote the apical closure of open apex teeth with great clinical success. Both authors reported the scaffold preparation as centrifuging the collected blood at 3600 rpm and 3000 rpm, respectively, for 10 min. Murray et al. systematic review supports the original studies’ findings and concludes that PRF and PRP can promote apical closure better than blood clots when used in revascularization procedures [32]. Saxen et al. added more information, reporting that PRF yields better outcomes than PRP [33]. Regarding the manipulation of the concentrates, Santhakumar et al. reported that PRF in the form of a membrane was easier to manipulate than as a clot when performing its clinical application [34].

#### 2.3.4. Apical Surgery

Eighty of the included studies reported the use of PRF and PRP in apical/ endodontic root end surgery (Table 1). Of those, 59 described the use of PRF, 9 described the use of PRF and PRP, and 12 described the use of PRP. The study types include 27 case reports, 16 RCTs, 15 RCTs protocols, 7 case series, 10 narrative reviews, and 5 systematic reviews. In most studies, PRF was used in the management of periapical lesions such as cysts, applied alone or in combination with bone graft materials to fill the bone defect. There are also reports where the platelet concentrates were used as retrograde filling material in apicectomy cases.

Regarding the five most recent articles on this procedure, two systematic reviews [35,36], one RCT protocol [37], one narrative review [17], and one case report [38] were selected. Table S6 presents the information retrieved from each mentioned article.

All sampled studies confirm the high success of the use of PRF and PRP in apical surgery cases. The reports support the claim that concentrates enhance periapical tissue regeneration and bone healing, which was attributed to their biological properties and their ability to bond directly to the bone. Interestingly, several studies report less pain and swelling in the cases where PRF and/or PRP were used, which is a relevant result from the patient’s perspective. As a limitation, only the study by Govindaraju et al. reported the PRF preparation method (3000 rpm for 10 min) [38].

#### 2.3.5. Reimplantation of Avulsed Tooth and Intentional Reimplantation

Of the 412 studies that reported the use of PRF and/or PRP in endodontic procedures, 13 reported its use in reimplantation of avulsed teeth or intentional reimplantation (Table 1). Of those, eight studies describe the use of PRF, two studies describe the use of PRF and PRP, and three studies describe the use of PRP. The records obtained for this type of procedure include six case reports, two systematic reviews, two animal studies, one case series, and two narrative reviews. Anterior teeth were the most reported teeth type for this procedure.

The representative sample in this procedure includes one case series [39], two case reports [19,40], one animal study [41], and one systematic review [18]. Detailed information in each report is provided in Table S7.

The reports on the reimplantation of avulsed teeth or intentional reimplantation represent a diversity of clinical situations where PRF and/or PRP were used. Yang et al. [39] present two cases where avulsed incisors were reimplanted and PRF small granules were used. In the case report of Parthasarathy et al. [40], both central incisors were avulsed, and root canal treatment was made extraoral before the teeth were reimplanted. Yang et al. [19] reported a case of intentional reimplantation of a mature maxillary premolar with sinus tract, fractured dens evaginatus, and internal root resorption with L-PRF. In Behnhaz et al. [41] animal study, the PRF effect of delayed tooth reimplantation on beagle dogs was evaluated. Overall, all studies show that using the concentrates contributes to healthy and intact periapical structures, decreased mobility, enhanced bone formation, reduced ankylosis, and less inflammatory resorption. In vitro studies support such results, showing that PRF increases periodontal ligament proliferation and bone regeneration.

The PRF and PRP can be applied in both the socket and intracanal, as reported in the systematic review by Khurshid et al. [18].

Regarding the scaffold preparation, only Yang et al. [19] (400× *g* for 10 min) and Benhaz et al. [41] (2700 rpm for 12 min) reported the preparation method.

#### 2.3.6. Autotransplantation

Autotransplantation is one of the less described procedures where PRF and/or PRP are being used, with only 2 studies from the 412 included ones reporting it (Table 1) reported the use of PRP, a case report [42], and a systematic review [43].

As detailed in Table S8, Gavinõ Orduña et al. published a case report where a 22-year-old female inferior first premolar was extracted and transplanted to the alveolar socket of her upper first premolar using the fragile fracture technique and extraoral apicectomy. PRP was prepared using the commercial PRGF-Endoret kit and centrifuged using a BTI System centrifuge (BTI Biotechnology Institute S.L., Miñano Meno, Spain), and half of the PRP, inactivated, was placed in the apical portion of the tooth, and the other half was activated and injected into the alveolar socket. The tooth was then placed in the receptor site. The authors reported clinical success with no root resorptions or pulp canal calcifications during follow-up and a positive response to cold tests [42]. The systematic review by Iqbal et al. included the above-mentioned case report and concludes there is reasonable evidence that pulp regeneration can be achieved via tissue engineering with PRP and PRF in transplantation cases [43].

#### 2.3.7. Biological Effects

Among the 412 included studies, 38 reported the evaluation of the biological effects and properties of PRF and PRP (Table 1). Of those, 21 reported the use of PRF, 11 reported the use of PRF and PRP, and 6 reported the use of PRP. The reports include 23 in vitro studies, 11 narrative reviews, 2 systematic reviews, 2 animal studies, and 1 RCT. One of the studies reports both an animal and an in vitro study.

The sample of the five most recent studies on this procedure (presented in Table S9) is composed of one systematic review of in vitro studies evaluating the antimicrobial effect of PRF [44], two in vitro studies, one evaluating the cytotoxicity of different pulp capping agents and another analyzing the interaction of PRF with MTA [45,46], and two narrative reviews describing the effects and application of PRF and PRP in pulp tissue healing [47].

Overall, the studies confirm the biological properties of PRF and PRP. The systematic review by Moraschini et al. [44] shows there is some evidence that PRF possesses antibacterial capacity, and Khatri et al. [46] concluded that PRF influences the pH and Ca^2+^ ion release from MTA over time. Panda et al. reported that PRF promoted the highest cell viability after 48 h and the highest potential to enhance cell differentiation and proliferation as a pulp-capping agent [45]. Elver et al. reported PRF affects angiogenesis, accelerating new blood vessel formation and increasing cell viability, decreasing inflammation, and accelerating bone regeneration [11]. The same conclusions were obtained in the study by Sandra et al., reporting PRF promotes vasculogenesis, increases cell migration, proliferation, and differentiation, and is less biologically risky than PRP as it is not biochemically manipulated [47]. All studies used standard protocols for PRF obtention, with only small variations.

#### 2.3.8. Endo-Perio Lesions

Sixteen reports from a total of 412 included studies report the use of PRF and/or PRP in the management of endo-perio lesions (Table 1). Of those, 13 used PRF, 1 used PRF and PRP, and 2 used PRP. Regarding the study types, the record includes five case reports, five RCTs, two narrative reviews, two systematic reviews, one case series, and one scoping review. The reports describe several procedures where endo-perio lesions were managed, including performing endodontic conventional treatment first and then periodontal surgical phase; endodontic treatment at the same time as periodontal surgical treatment; tooth intentional reimplantation with extraoral root canal treatment and apicectomy or endodontic apical surgery followed by periodontal surgery at the same time.

The records sampled for these procedures were composed of two RCTs [48,49], two systematic reviews [50,51], and one scoping review [52], as detailed in Table S10.

Choudhary et al. compared titanium-PRF to PRF in endo-perio lesions in molars, and both had positive outcomes [49]. Thakur et al. reported the management of necrotic teeth with endo-perio lesions with PRF clots being used to fill bone defects after apicectomy and PRF membranes used in the root surfaces with the increasing of satisfactory periapical healing [48]. Both Oktawati et al. [50] and Onicas et al. [51] reported that the use of PRF in endo-perio lesions leads to a decrease in probing depth and periodontal ligament healing. Ardila et al. scoping review confirmed the original studies’ results, showing promising results when PRF was applied [52]. Regarding the protocol used to produce PRF, Onicas et al. [51] used a relative centrifugal force of 700× *g* to 200× *g* for 12 min, Thakur et al. [48] 2700 rpm for 12 min, and Choudhary et al. [49] 3000 rpm for 10 min.

#### 2.3.9. Root Fracture

Root fracture management using PRF and/or PRP was one of the least reported procedures, with only three studies referring to it (Table 1). Both reports described the use of PRF and correspond to one case series [31] and two case reports [20,53].

As shown in Table S11, the three studies refer to anterior teeth with root fractures at the apical third, although the case reported by Kapoor et al. [20] also presented a fracture along the root and Arango-Goméz et al. [53] various horizontal fractures in the apical and middle thirds. Both Kapoor et al. [20] and Pruthi et al. [31] cases combine the use of PRF with MTA or Biodentine to manage the fracture, and Arango Goméz et al. [53] combined PRP with MTA. The outcomes were favorable in the three cases described. The case presented by Kapoor et al. [20] showed a decrease in probing depth, mobility, and bone regeneration. In the case presented by Pruthi et al. [31], no tooth mobility at follow-up was observed, and the fracture site was repaired by interposition of connective tissue. Arango-Goméz et al. reported a complete remission of symptoms and that the tooth treated with PRP showed better calcification of the fractures than the tooth treated with standard blood clot revascularization [53]. As a limitation, the case report by Kapoor et al. did not describe the preparation of the PRF protocol [20].

#### 2.3.10. Management of Root Perforation

The use of PRF and/or PRP for the management of root perforations was described in 10 articles of the 412 included (Table 1). Of those, eight studies report the use of PRF, and two studies report the use of PRF and PRP. The study types include four case reports, three narrative reviews, two animal studies, and one case series. The most frequent perforations reported were strip perforations or pulp floor perforations in permanent molars.

The five more recent articles on this procedure, selected using the sampling method, are presented in Table S12. The records include one animal study [54], two case reports [55,56], one narrative review [11], and one case series [31].

The case reports and case series describe severely compromised teeth with strip and furcal perforations. The case management was performed using conventional root canal filling combined with surgical procedures, where PRF was used alone (Pruthi et al. [31]) or in combination with MTA (Teja et al. [55]) or Bio-oss (Cordova-Malca et al. [56]). The cases were successfully treated, with signs of tissue healing. In an animal study, Mohamed et al. compared the association of CGF + MTA, PRF + MTA, and MTA alone in the management of contaminated and non-contaminated furcal perforations in dogs’ molars. PRF and CFG groups demonstrated superior bone formation and fewer inflammatory cells than the MTA group [54].

All studies used standard protocols for PRF obtention, with only small variations.

#### 2.3.11. Mechanical Properties

Together with the root fracture procedures, studies describing the evaluation of mechanical properties of PRF and/or PRP were the least reported ones, with only two included (Table 1). Both studies analyzed the mechanical properties of PRF and were performed in vitro [57,58].

As described in Table S13, the studies focus on analyzing diverse parameters of PRF, such as tensile strength, degradation, cellular distribution, pH, and morphology. Those properties were then compared with other materials.

#### 2.3.12. Management of Root Resorption

Management of root resorptions using PRF and/or PRP was reported by 5 of the 385 included studies (Table 1). Of those, three studies used PRF, and two studies used PRP. The study types include four case reports and one systematic review.

The five representative articles selected as a sample for this procedure are composed of four case reports [20,59,60,61] and one systematic review [62]; these are presented in Table S14.

The included cases describe PRF application both in the canal at the lesion site or applied externally at the root surface. In two cases, PRF was combined with hydroxyapatite. The lesions were arrested in all cases with favorable periodontal and bone outcomes. Regarding the scaffold preparation, Gupta et al. [60] and Johns et al. [59] used the standard 3000 rpm for 10 min, while Nalawade et al. [61] did not report the centrifuged velocity and detailed that, before the application, PRP was mixed with bioactive synthetic bone draft, thrombin, and calcium chloride.

#### 2.3.13. Regeneration of Mature Teeth

The regeneration of mature teeth using PRF and/or PRP is reported by 39 articles (Table 1). Of those, 16 describe the use of PRF, 7 describe the use of PRF and PRP, and 16 describe the use of PRP. These articles include 12 narrative reviews, 7 RCTs, 6 RCTs protocols, 6 systematic reviews, 5 animal studies, and 3 case reports.

The representative sample of the five most recent reports on this procedure is composed of one animal study [63], two RCTs [64,65], one systematic review [66], and one narrative review [11]. The details of each study are presented in Table S15.

The sampled studies confirmed the success of PRF and PRP in the revascularization of mature necrotic teeth. The positive outcomes include higher scores in patients’ satisfaction and teeth function. The animal study by Eldessoky et al. confirmed that new tissue formed inside the root canal, which resembled periodontal connective tissue, with inflammatory cells and a moderate number of blood vessels after PRF was used in revascularization [63]. The systematic review by Li et al. confirmed such results, describing success rates for mature teeth as 95% [66]. Also, in the narrative review by Elver et al., PRF has been proven to increase healing and lead to better outcomes and prognosis of endodontic regenerative approaches for mature teeth [11].

Regarding scaffold preparation, Elver et al. [11] and Eldessoky et al. [63] used standard preparation methods, while Ahmed et al. [64] reported the preparation of PRP using a sodium citrate vacuum tube, single centrifugation technique at 460× *g* for 8 min, collection of PRP using an automatic pipette and transferred to a sterile plain vacuum chloride to be used as liquid.

## 3. Discussion

The aim of this scoping review was to identify, map, and analyze the existing literature on the application of PRF, L-PRF, and PRP in endodontic procedures. Additionally, it was intended to collect information on the protocols being used for PRF and PRP preparation and to collect bibliographic data from the published studies.

The collected information supports that PRF and PRP present great utility in endodontics, being used in almost all endodontic procedures, mostly in regenerative procedures. The use of platelet concentrate has been increasing in all medical fields, and dentistry has been following the same tendency [67]. The interest in PRF and PRP can be explained by their relevant biological properties, mainly due to the high concentration of platelets. This high concentration is responsible for a high concentration of platelet growing factors such as platelet-derived growth factor (PDGF); insulin-like growth factor 1 and 2 (IGF-1 and IGF-2); various interleukins, including interleukin-1 (IL-1), interleukin-4 (IL-4) and interleukin-6 (IL-6); vascular endothelial growth factor (VEGF);, and transforming growth factor B1 (TGF-B1) [5,68]. Consequently, PRF and PRP present several relevant properties: anti-fungal and antimicrobial, anti-inflammatory, angiogenic, osteo-inductive, and analgesic [68].

Also, PRF can be combined with various biomaterials, such as MTA, Biodentine, and hydroxyapatite, among others, to improve treatment outcomes. Due to their properties, platelet derivates can also be used as a carrier for antibiotics, steam cells, or drugs, boosting the procedure’s efficacy. The success of such a combination is described in the literature. For instance, a study by Niemczyk et al. supports the idea that different formulations of platelet concentrates are suitable carriers for antibiotics [69]. These results were also supported by a systematic review which concluded that the mechanical properties of the PRF clots are favorable to the incorporation of antibiotics, resulting in increased antimicrobial activity of the scaffold for long periods of time, with continuous release of the antibiotic at the surgical site, and consequent improved healing and decrease in postoperative pain and infection [70]. Hoveizi et al. also reported the incorporation of odontoblasts in a fibrin gel and combined with bone morphologic protein-2 for pulp capping procedures. Using an animal model in rats, the authors showed that the combination was successful in promoting dentin regeneration [71].

Regarding the endodontic procedures where PRF and PRP have been used, regeneration of immature teeth is the most reported one, with about half of the included studies referring to this procedure. Several reasons can explain such a fact. First, the loss of vitality in immature teeth presents a greater impact than in mature teeth. It leads to an incomplete development of the tooth, with an open apex, a more fragile structure, and an incapacity to respond to aggressions [72,73]. This way, treatments that can promote the revascularization of immature teeth and, consequently, the continuation of tooth development are of fundamental importance and are widely explored in the clinic. Second, international guidelines on the topic, such as the American Association of Endodontics Clinical Considerations for Regenerative Procedures, recommended REP to be performed in immature teeth due to the above-mentioned issues [74]. Third, the biological and mechanical properties of PRF and PRP make them excellent materials for use as a scaffold in REP [11,12,45]. Even more, its autologous origin avoids potential rejection issues [12]. The same regenerative and anti-inflammatory properties can be used to explain the high number of papers reporting the use of concentrates in procedures such as pulpotomy and pulp capping or apicectomy [10,45].

On the other hand, few reports were identified for some procedures such as intentional reimplantation, root canal perforations and root canal resorptions treatment, autotransplantation, and root canal fractures. This limited body of evidence can be explained by the frequency of such procedures in the clinic. They are less frequent and/or represent terminal treatment options; hence, they are less reported [19,75]. Also, professionals may lack information on the possible use of platelet concentrates to manage such situations since there are no protocols or guidelines defined for using PRF and/or PRP in such procedures. Moreover, the standard choices are conventional treatments already described or extraction [75,76,77].

Regarding the endodontic procedures where PRF and/or PRP are being used, although it was not the aim of this review, the gathered information allowed us to draw some conclusions about their efficacy. Most studies report similar or even higher success rates of PRF and PRP when compared to standard materials or procedures. These high success rates were reported in procedures such as RET in immature teeth [25,26,27,28], pulpotomy and pulp capping procedures [15,21], apexification [30,31,32,34], apical surgery [17,35,36,38], tooth reimplantation [18,19,39,40,41], autotransplantation [42,43], endodontic-periodontal lesions management [48,49,50,51,52], root fracture treatment [20,31,53], management of root iatrogenic perforations [11,31,54,55], root resorptions healing [59,60,61,62] and mature teeth revascularization [11,63,64,65,66]. Such positive results allow us to consider PRF and PRP as predictable and reproducible procedures. Interestingly, PRF and PRP can be used instead or in combination with several materials such as MTA, Biodentine, bone substitutes, or blood cloth, expanding their clinical indications. However, it should be mentioned that, for some procedures, due to the limited number of articles and types of studies included, no definitive conclusion about their efficacy can be drawn.

In this review, only the original Choukroun’s PRF and L-PRF subtypes were included. Although some variations in the protocol exist, most articles report the centrifuging of venous blood at 3000 rpm or 2700 rpm for 10 or 12 min, respectively, which allows obtaining PRF with similar characteristics. On the other hand, several protocols and commercial kits for PRP obtention are described in the literature. The main differences refer to centrifugation forces and time, performing one or two centrifugations, and the type of anticoagulant added [43]. If two centrifugations are made, there is also variation in the second phase among commercial kits [9]. These differences in the protocols used for obtaining the concentrates make it difficult to determine the correct nomenclature of the concentrate and compare the results of different studies. Future studies on this topic should compare the different protocols and establish the more efficient one. Also, the studies should report centrifugation forces in relative centrifugal force (RCF)/g-force or the rotor radius to allow the studies reproducibility and comparison, as most studies only report rpm.

Although reports on the use of both PRF and PRP have been included in this review, the majority describe the use of PRF, with 234 reporting the use of PRF and 91 the use of PRF and PRP, compared with only 87 records reporting the use of PRP (Table 1). This difference can have several explanations. The authors report that PRF is easier to handle due to its ability to turn a clot into a membrane [34]. Furthermore, the obtention of PRF is easier, less time-consuming, cheaper than PRP, and without the need to add anticoagulants and/or activators. The addition of such agents can also negatively influence the wound healing and tissue regeneration properties of the agent [11]. It should also be noted that PRP protocols using two centrifugation phases are more sensitive to human error and less reproducible [9,68]. Finally, many authors report that the mechanical properties of PRF are better compared to PRP. The polarized fibrin matrix with the platelet growing factors trapped in the networks allows a release along the time, contributing to better chemotaxis and wound healing [6].

Considering the type of studies included, case reports represent most of the primary research studies. A possible explanation for such a high prevalence of this study type is how easily it is performed, besides being less time- and resource-consuming, mainly for clinicians. Case reports mostly focus on relevant clinical cases that are not performed on a daily basis or present unusual findings. Also, they represent situations where non-standardized treatments were performed many times [78].

Animal and in vitro studies are also fundamental to understanding PRF and PRP mechanisms of action in each procedure and tissue since they allow a more in-depth analysis, such as a histological one. More studies of these types should be conducted since there are still mechanisms of action of the concentrates on the tissues that are not yet fully understood.

Important aspects to mention are the number of publications on this topic increasing every year and the number of RCT protocols included in this review. Such a high number supports this a topic of interest for both clinicians and scientists. Consequently, there should be an increase in the number of publications on this topic in the next few years, which could help increase the evidence on the use of PRF and PRP in endodontic procedures.

Although this review represents an important step in mapping the information available on this topic, some limitations exist. This scoping review was developed focusing on PRP, the second-generation platelet concentrate developed by Choukroun in 2001, and the leukocyte and platelet-rich fibrin, which are the most widely used protocols for PRF. Therefore, it only included reports describing the two types of PRF protocols listed above and PRP from the vast group of platelet concentrates. Therefore, future reviews on this topic could include all the concentrate types and expand the information available. Furthermore, although no bias evaluation was performed during the data extraction process, it was possible to see that several articles present methodological issues, from the study design to the information available in the reports, which limits the evidence obtained. The already mentioned variability in PRF and PRP obtention protocols and nomenclature is also a relevant aspect to mention since it may bias the information mapped. Finally, for the list of authors, only the first author was considered, which may misrepresent the most prolific authors in this field.

In this way, a more understanding map of information can be achieved for clinicians, and gaps in knowledge can be better recognized and addressed.

## 4. Materials and Methods

### 4.1. Protocol Registration

This review was planned and reported following the Preferred Reporting Items for Systematic Reviews and Meta-Analysis Protocols, scoping review extension guidelines (PRIMSA-ScR) [79], and the recommendations by the Joan Briggs Institute [80]. The PRISMA-ScR checklist is presented in Table S16.

The review protocol was registered in the Open Science Framework (OSF) platform before the review began and can be assessed at https://doi.org/10.17605/OSF.IO/6UY49.

### 4.2. Review Questions

The following research question was drafted following the PCC (Population, Concept, Context) framework:

“What are the applications of PRP and PRF in endodontics?” (Table 2)

### 4.3. Information Sources

Four databases were searched for relevant articles: Medline (using PubMed), Cochrane Library, Web of Science (all databases), and Embase. The literature search was conducted from inception until 20 December 2023. Additionally, the references of included studies were searched to find other potentially relevant papers.

### 4.4. Search Strategy

A search strategy combining relevant Medical Subject Headings (MeSH) terms and keywords was created for PubMed and adapted to the other databases. Language (articles published in Portuguese, English, Spanish, and French) and type of publication filters were used to refine the search. The complete search strategy is presented in Table S17.

### 4.5. Eligibility Criteria

Inclusion and exclusion criteria were defined before the literature search to determine the studies to be included.

Inclusion criteria: studies that describe the use of L-PRF, Choukroun’s PRF, or PRP in endodontic procedures. Any study type was considered.

Exclusion criteria: studies were excluded if they did not report the use of L-PRF, PRF, or PRP or if they did not report endodontic procedures.

### 4.6. Selection Process

The literature search results were imported to Ryyan software, and the duplicates were removed [81]. After, the studies were screened first by title and abstract and later by full text independently by two authors (S.G. and C.M.M.), considering the pre-defined inclusion and exclusion criteria. In case of conflicting opinions about a study inclusion, a third author was consulted (S.P.), and a decision was reached by consensus.

### 4.7. Data Extraction

The data extraction occurred in two phases. In the first phase, information on the type of study, type of platelet concentrate, first author name, publication year, journal where the study was published, and type of endodontic procedure was collected from the 412 records.

The studies were then organized by type of endodontic procedure. In the second extraction phase, and considering the number of included studies, a sampling method was employed, and the five most recent ones were selected for complete data extraction [82]. From the sampled articles, the following information was collected: sample size; sample characterization; periapical and pulp diagnosis; study protocol; agent used and the protocol for its preparation; clinical procedure being studied; outcome of the treatment and follow-up period(s). For the extracted data synthesis, the studies were grouped by the type of endodontic procedure and presented in a tabular manner, complemented with a narrative approach.

## 5. Conclusions

The platelet concentrates, PRF and PRP, are being used in several endodontic procedures, including the revascularization of immature teeth, apical surgery, and pulpotomy/pulp capping the main ones. Most studies report the use of PRF, probably due to the easier obtention protocol, less time-consuming process, and cheaper biological and mechanical properties. The success rate of PRF and PRP use is comparable to or even higher than standard procedures and materials. There is high variability in the PRF and PRF obtention protocols, which limits the comparison of the obtained results. Future studies on this topic should use standardized preparation protocols and evaluation outcomes. An effort should be made to summarize the available evidence and create protocols for clinicians to incorporate platelet concentrates in their daily practice.

## Figures and Tables

**Table 1:** Characteristics of the included studies.

	*n*	%
STUDY TYPE		
Case report	93	22.52%
Narrative review	66	13.98%
Randomized controlled trial	62	15.01%
Randomized controlled trial protocol	52	12.59%
Systematic review	42	10.17%
Animal	34	8.23%
Case-series	27	6.54%
In vitro	24	5.81%
Cohort	5	1.21%
Case-control	3	0.73%
Scoping review	3	0.73%
Umbrella review	2	0.48%
Total (Studies)	413	100%
TYPE OF PROCEDURE		
Regeneration of immature teeth	185	44.90%
Apical surgery	80	19.42%
Pulpotomy/Pulp capping	56	13.59%
Regeneration of mature teeth	39	9.47%
Biological properties evaluation	38	9.22%
Apexification	20	4.85%
Endo-perio lesions	16	3.88%
Intentional reimplantation	13	3.16%
Perforation	10	2.43%
Resorption	5	1.21%
Root fracture	3	0.73%
Auto transplant	2	0.49%
Mechanical properties evaluation	2	0.49%
Total (Articles)	412	100%
TOP 10 JOURNALS		
Journal of Endodontics	55	13.35%
International Endodontic Journal	20	4.85%
Journal of Conservative Dentistry	19	4.61%
Journal of Clinical and Diagnostic Research	17	3.13%
Contemporary Clinical Dentistry	10	2.43%
International Journal of Pediatric Dentistry	8	1.94%
Restorative Dentistry and Endodontics	8	1.94%
The Journal of Contemporary Dental Practice	7	1.70%
Indian Journal of Dental Research	7	1.70%
BMC Oral Health	6	1.46%
Total (Articles)	412	100%
TOP 10 FIRST AUTHORS		
Johns, D. A.	4	0.97%
Jadhav, G.R.	4	0.97%
Torabinejad, M.	4	0.97%
Meschi, N.	4	0.97%
Chen, Y.	3	0.73%
Alawwad, M.	3	0.73%
Bezgin, T.	3	0.73%
Shivashankar, V. Y.	3	0.73%
Nagaraja, S.	3	0.73%
Hiremath, H.	3	0.73%
Total (Articles)	412	100%
PUBLICATION YEAR		
2023	41	9.95%
2022	53	12.86%
2021	43	10.44%
2020	56	13.59%
2019	31	7.52%
2018	21	5.10%
2017	34	8.25%
2016	34	8.25%
2015	27	6.55%
2014	21	5.01%
2013	21	5.01%
2012	13	3.16%
2011	9	2.18%
2010	3	0.73%
2009	3	0.73%
2008	1	0.24%
2007	1	0.24%
2006	0	0%
2005	0	0%
2004	1	0.24%
Total (Articles)	412	100%

**Table 2:** PCC question.

PCC	
Population	Patients and experimental models of endodontic treatment
Concept	PRF and PRP application
Context	Endodontic treatment

## Data Availability

The original contributions presented in this study are included in the article/Supplementary Materials. Further inquiries can be directed at the corresponding authors.

## Supplementary Materials

The following supporting information can be downloaded at: https://www.mdpi.com/article/10.3390/ijms26125479/s1.

## Abbreviations

The following abbreviations are used in this manuscript:

A-PRF	Advanced platelet-rich fibrin
CGF	Concentrated growth factors
IGF-1	Insulin-like growth factor 1
IGF-2	Insulin-like growth factor 2
I-PRF	Injectable platelet-rich fibrin
IL-1	Interleukin-1
IL-4	Interleukin-4
IL-6	Interleukin-6
L-PRF	Leucocyte platelet-rich fibrin
MeSH	Medical subject headings
MTA	Mineral trioxide aggregate
OSF	Open science framework
P-PRP	Pure platelet-rich plasma
PCC	Population, concept, context
PDGF	Platelet-derived growth factor
PRF	Platelet-rich fibrin
PRISMA-ScR	Preferred reporting items for systematic reviews and meta-analysis protocols, Scoping review extension guidelines
PRP	Platelet-rich plasma
RCT	Randomized controlled trial
RET	Regenerative endodontic treatment
RPM	Revolution per minute
T-PRF	Titanium platelet-rich fibrin
TGF-B1	Transforming growth factor B1
VEGF	Vascular endothelial growth factor

## References

[1] Morotomi T., Washio A., Kitamura C. (2019). Current and Future Options for Dental Pulp Therapy. Jpn. Dent. Sci. Rev..

[2] Murray P.E., Garcia-Godoy F., Hargreaves K.M. (2007). Regenerative Endodontics: A Review of Current Status and a Call for Action. J. Endod..

[3] Gathani K., Raghavendra S. (2016). Scaffolds in Regenerative Endodontics: A Review. Dent. Res. J. (Isfahan).

[4] Zhang W., Yelick P.C. (2021). Tooth Repair and Regeneration: Potential of Dental Stem Cells. Trends Mol. Med..

[5] Hotwani K., Sharma K. (2014). Platelet Rich Fibrin—A Novel Acumen into Regenerative Endodontic Therapy. Restor. Dent. Endod..

[6] Agrawal A.A. (2017). Evolution, Current Status and Advances in Application of Platelet Concentrate in Periodontics and Implantology. World J. Clin. Cases.

[7] Whitman D.H., Berry R.L., Green D.M. (1997). Platelet Gel: An Autologous Alternative to Fibrin Glue With Applications in Oral and Maxillofacial Surgery. J. Oral Maxillofac. Surg..

[8] Umakanth K., Balaji Ganesh S., Smiline Girija A. (2020). Applications Of Platelet Concentrates In Endodontics—A Review. Int. J. Pharm. Res..

[9] Dohan Ehrenfest D.M., Rasmusson L., Albrektsson T. (2009). Classification of Platelet Concentrates: From Pure Platelet-Rich Plasma (P-PRP) to Leucocyte- and Platelet-Rich Fibrin (L-PRF). Trends Biotechnol..

[10] Arshad S., Tehreem F., Rehab khan M., Ahmed F., Marya A., Karobari M.I. (2021). Platelet-Rich Fibrin Used in Regenerative Endodontics and Dentistry: Current Uses, Limitations, and Future Recommendations for Application. Int. J. Dent..

[11] Elver A., Caymaz M.G. (2023). Novel Approaches to the Use of Platelet-Rich Fibrin: A Literature Review. Saudi Dent. J..

[12] Pietruszka P., Chruścicka I., Duś-Ilnicka I., Paradowska-Stolarz A. (2021). PRP and PRF—Subgroups and Divisions When Used in Dentistry. J. Pers. Med..

[13] Fujioka-Kobayashi M., Miron R.J., Hernandez M., Kandalam U., Zhang Y., Choukroun J. (2017). Optimized Platelet-Rich Fibrin With the Low-Speed Concept: Growth Factor Release, Biocompatibility, and Cellular Response. J. Periodontol..

[14] Bolhari B., Meraji N., Ghorbanzadeh A., Sarraf P., Moayeri R. (2019). Applications of Fibrin-Based Products in Endodontics: A Literature Review. Dent. Hypotheses.

[15] Eid A., Mancino D., Rekab M.S., Haikel Y., Kharouf N. (2022). Effectiveness of Three Agents in Pulpotomy Treatment of Permanent Molars with Incomplete Root Development: A Randomized Controlled Trial. Healthcare.

[16] Riaz A., Shah F.A. (2021). Regenerating the Pulp–Dentine Complex Using Autologous Platelet Concentrates: A Critical Appraisal of the Current Histological Evidence. Tissue Eng. Regen. Med..

[17] Alsolaihim A., Alsolaihim A., Alsolaihim N., Alowais L. (2023). Biomimetic Regenerative Materials in Restorative Dentistry and Endodontics. J. Int. Oral. Health.

[18] Khurshid Z., Asiri F.Y.I., Najeeb S., Ratnayake J. (2022). The Impact of Autologous Platelet Concentrates on the Periapical Tissues and Root Development of Replanted Teeth: A Systematic Review. Materials.

[19] Yang Y., Zhang B., Huang C., Ye R. (2021). Intentional Replantation of a Second Premolar with Internal Resorption and Root Fracture: A Case Report. J. Contemp. Dent. Pract..

[20] Kapoor S. (2015). Surgical Management of a Non-Healing Intra-Alveolar Root Fracture Associated with Pulpal Calcification and Root Resorption: A Case Report. J. Clin. Diagn. Res..

[21] Mohammed S.E., Gawdat S.I., Ibrahim S.M. (2022). Role of Prf With Mta and Theracal After Pulpotomy In Relieving Pain and Maintaining the Vitality of the Remaining Radicular Pulp Tissue in Permanent Posterior Teeth with Closed Root Apices: “Randomized Controlled Trial”. J. Pharm. Negat. Results.

[22] Mandviwala D.K., Arora A.V., Kapoor S.V., Shah P.B. (2022). Internal Root Resorption: A Rare Complication of Vital Pulp Therapy Using Platelet-Rich Fibrin. J. Oral. Maxillofac. Pathol..

[23] (2023). Ctri Pulpotomy in Mature Permanent Tooth Using Various Biomaterials. https://trialsearch.who.int/Trial2.aspx?TrialID=CTRI/2023/06/053989.

[24] (2023). Ctri Comparasion of Success Rate of Pulpotomy with Biodentine Using PRF Membrane and Collagen Scaffold in Permanent Molars. https://trialsearch.who.int/Trial2.aspx?TrialID=CTRI/2023/03/050834.

[25] Naik S.V., Attiguppe P., Prakash A.J. (2023). Comparative Evaluation of the Regenerative Potential of Blood Clot and Platelet-Rich Fibrin in Young Permanent Teeth Based on the Revised American Academy of Endodontics Clinical Considerations for Regenerative Procedure: 2016. Int. J. Clin. Pediatr. Dent..

[26] Das S., Srivastava R., Thosar N.R., Khubchandani M., Ragit R., Malviya N. (2023). Regenerative Endodontics-Reviving the Pulp the Natural Way: A Case Report. Cureus.

[27] Kumar J.K., Surendranath P., Eswaramoorthy R. (2023). Regeneration of Immature Incisor Using Platelet Rich Fibrin: Report of a Novel Clinical Application. BMC Oral Health.

[28] Ríos-Osorio N., Caviedes-Bucheli J., Jimenez-Peña O., Orozco-Agudelo M., Mosquera-Guevara L., Jiménez-Castellanos F., Muñoz-Alvear H. (2023). Comparative Outcomes of Platelet Concentrates and Blood Clot Scaffolds for Regenerative Endodontic Procedures: A Systematic Review of Randomized Controlled Clinical Trials. J. Clin. Exp. Dent..

[29] Li J., Zheng L., Daraqel B., Liu J., Hu Y. (2023). The Efficacy of Concentrated Growth Factor and Platelet-Rich Fibrin as Scaffolds in Regenerative Endodontic Treatment Applied to Immature Permanent Teeth: A Retrospective Study. BMC Oral Health.

[30] Biradar N., Ragulakollu R., Bogishetty C., Tej G., Gandham S., Vardhan P. (2023). Combination Therapy of Antibiotics and Platelet-Rich Fibrin for Apical Closure: Case Series. Int. J. Clin. Pediatr. Dent..

[31] Pruthi P.J., Goel S., Yadav P., Nawal R.R., Talwar S. (2020). Novel Application of a Calcium Silicate—based Cement and Platelet-Rich Fibrin in Complex Endodontic Cases: A Case Series. Gen. Dent..

[32] Murray P.E. (2018). Platelet-Rich Plasma and Platelet-Rich Fibrin Can Induce Apical Closure More Frequently Than Blood-Clot Revascularization for the Regeneration of Immature Permanent Teeth: A Meta-Analysis of Clinical Efficacy. Front. Bioeng. Biotechnol..

[33] Hugar S.M., Gokhale N., Soneta S.P., Joshi R.S., Dialani P.K., Saxena N. (2022). Evaluation of the Treatment Protocols in the Management of Pulpally Involved Young Permanent Teeth in Children: A Systematic Review and Meta-Analysis. Int. J. Clin. Pediatr. Dent..

[34] Santhakumar M., Yayathi S., Retnakumari N. (2018). A Clinicoradiographic Comparison of the Effects of Platelet-Rich Fibrin Gel and Platelet-Rich Fibrin Membrane as Scaffolds in the Apexification Treatment of Young Permanent Teeth. J. Indian Soc. Pedod. Prev. Dent..

[35] di Lauro A.E., Valletta A., Aliberti A., Cangiano M., Dolce P., Sammartino G., Gasparro R. (2023). The Effectiveness of Autologous Platelet Concentrates in the Clinical and Radiographic Healing after Endodontic Surgery: A Systematic Review. Materials.

[36] Sinha A., Jain A.K., Rao R.D., Sivasailam S., Jain R. (2023). Effect of Platelet-Rich Fibrin on Periapical Healing and Resolution of Clinical Symptoms in Patients Following Periapical Surgery: A Systematic Review and Meta-Analysis. J. Conserv. Dent..

[37] (2023). Nct Post-Operative Evaluation of Endodontic Microsurgeries Done Using a Piezoelectric Ultrasonic Technique: An in Vivo Study. https://clinicaltrials.gov/show/NCT05863728.

[38] Govindaraju L., Antony D.P., S P. (2023). Surgical Management of Radicular Cyst With the Application of a Natural Platelet Concentrate: A Case Report. Cureus.

[39] Yang Y., Liu Y.-L., Jia L.-N., Wang J.-J., Zhang M. (2023). Rescuing “Hopeless” Avulsed Teeth Using Autologous Platelet-Rich Fibrin Following Delayed Reimplantation: Two Case Reports. World J. Clin. Cases.

[40] Parthasarathy R., Srinivasan S., C V., Thanikachalam Y., Ramachandran A. (2022). An Interdisciplinary Management of Avulsed Maxillary Incisors: A Case Report. Cureus.

[41] Behnaz M., Izadi S.S., Mashhadi Abbas F., Dianat O., Sadeghabadi S., Akbarzadeh T., Haeri A., Kazem M., Younessian F. (2021). The Impact of Platelet-rich Fibrin (PRF) on Delayed Tooth Replantation: A Preliminary Animal Study. Aust. Endod. J..

[42] Gaviño Orduña J.F., García García M., Dominguez P., Caviedes Bucheli J., Martin Biedma B., Abella Sans F., Manzanares Céspedes M.C. (2020). Successful Pulp Revascularization of an Autotransplantated Mature Premolar with Fragile Fracture Apicoectomy and Plasma Rich in Growth Factors: A 3-year Follow-up. Int. Endod. J..

[43] Iqbal A., Riaz A., Waheed A., Khan S.U., Nawadat K., Islam S. (2021). Reorienting Goals in Endodontic Therapy: Pulp Revitalization, on the Brink of a Paradigm Shift. J. Pak. Med. Assoc..

[44] Moraschini V., Miron R.J., Mourão C.F.d.A.B., Louro R.S., Sculean A., da Fonseca L.A.M., Calasans Maia M.D., Shibli J.A. (2024). Antimicrobial Effect of Platelet-rich Fibrin: A Systematic Review of in Vitro Evidence-based Studies. Periodontol. 2000.

[45] Panda P., Govind S., Sahoo S.K., Pattanaik S., Mallikarjuna R.M., Nalawade T., Saraf S., Khaldi N.A., Jahdhami S.A., Shivagange V. (2023). Analysis of Pulp Tissue Viability and Cytotoxicity of Pulp Capping Agents. J. Clin. Med..

[46] Khatri S., Mathew S., Nagaraja S., Hegde S., Ghosh S., Ravichandran K. (2023). Comparative Evaluation of PH and Ca+ Ion Release from MTA on Interaction with Platelet-Rich Fibrin and Blood Clot: An in Vitro Study. F1000Research.

[47] Sandra F., Sutanto A., Wulandari W., Lambertus R., Celinna M., Dewi N.M., Ichwan S.J.A. (2023). Crucial Triad in Pulp-Dentin Complex Regeneration: Dental Stem Cells, Scaffolds, and Signaling Molecules. Indones. Biomed. J..

[48] Thakur V., Mittal S., Tewari S., Kamboj M., Duhan J., Sangwan P., Kumar V., Gupta A. (2023). Comparative Histological Evaluation of Two PRF Formulations (PRF High and PRF Medium) on Quality of Life and Healing Outcome of Apicomarginal Defects: A Randomized Clinical Trial. J. Cranio-Maxillofac. Surg..

[49] Makkad R.S. (2023). Platelet-Rich Fibrin and Titanium-Prepared Platelet-Rich Fibrin in Endoperio Lesion Management. Bioinformation.

[50] Oktawati S., Siswanto H., Mardiana A., Supiaty, Neormansyah I., Basir I. (2020). Endodontic–Periodontic Lesion Management: A Systematic Review. Med. Clín. Práct..

[51] Onicas M.I., Narita L.E., Mester A., Onisor F., Mancini L. (2021). Platelet-Rich Fibrin: A Viable Therapy for Endodontic-Periodontal Lesions? A Preliminary Assessment. Appl. Sci..

[52] Ardila C.M., Vivares-Builes A.M. (2022). Clinical Efficacy of Treatment of Endodontic-Periodontal Lesions: A Systematic Scoping Review of Experimental Studies. Int. J. Environ. Res. Public Health.

[53] Arango-Gómez E., Nino-Barrera J.L., Nino G., Jordan F., Sossa-Rojas H. (2019). Pulp Revascularization with and without Platelet-Rich Plasma in Two Anterior Teeth with Horizontal Radicular Fractures: A Case Report. Restor. Dent. Endod..

[54] Mohamed D.A.-A., Abdelwahab S.A., Mahmoud R.H., Taha R.M. (2023). Radiographic and Immuno-Histochemical Evaluation of Root Perforation Repair Using MTA with or without Platelet-Rich Fibrin or Concentrated Growth Factors as an Internal Matrix in Dog’s Teeth: In Vivo Animal Study. Clin. Oral. Investig..

[55] Teja K., Ramesh S. (2021). Nonsurgical Management of Strip Perforation Using Platelet-Rich Fibrin and MTA by Matrix Concept—A Case Report with One Year Follow-Up. Contemp. Clin. Dent..

[56] Córdova-Malca F., Coaguila-Llerena H., Garré-Arnillas L., Rayo-Iparraguirre J., Faria G. (2022). Endodontic Micro-Resurgery and Guided Tissue Regeneration of a Periapical Cyst Associated to Recurrent Root Perforation: A Case Report. Restor. Dent. Endod..

[57] Nagaraja S., Mathew S., Rajaram R., Pushpalatha C., Abraham A., Chandanala S. (2019). Evaluation of Histological and PH Changes in Platelet-Rich Fibrin and Platelet-Rich Fibrin Matrix: A In Vitro Study. Contemp. Clin. Dent..

[58] Chhaya D., Vaidya N., Patel V., Chudasama K., Doshi S., Kumar P. (2022). Evaluation and Comparison of Mechanical Properties of Platelet-Rich Fibrin Membrane, Fish Collagen Membrane, Bovine Collagen Membrane and Chorionic Membrane—An SEM Study. Indian J. Dent. Res..

[59] Johns D., Shivashankar V., Maroli R., Joseph R. (2013). Invasive Cervical Root Resorption: Engineering the Lost Tissue by Regeneration. Contemp. Clin. Dent..

[60] Gupta G., Agarwal A., Ansari A.A., Singh R.K. (2022). Non-Surgical Management of a Large Periapical Lesion with Internal Resorption Using PRF, Hydroxyapatite and MTA. BMJ Case Rep..

[61] Nalawade T.M., Telgi C.R., Arora G., Rachappa M. (2011). Platelet Rich Plasma and Bone Graft for Rehabilitation of Luxation Injuries to Permanent Incisors. J. Adv. Oral. Res..

[62] Dadpe A.M. (2023). Regenerative Endodontic Procedures in Teeth with Root Resorption: A Systematic Review. Eur. Endod. J..

[63] Eldessoky A.E., Khalefa M.M., Abu-Seida A.M. (2023). Regenerative Endodontic Therapy in Mature Teeth with Necrotic Pulp and Apical Periodontitis Using Two Disinfection Protocols. BMC Oral Health.

[64] Ahmed Y.E., Ahmed G.M., Ghoneim A.G. (2023). Evaluation of Postoperative Pain and Healing Following Regenerative Endodontics Using Platelet-rich Plasma versus Conventional Endodontic Treatment in Necrotic Mature Mandibular Molars with Chronic Periapical Periodontitis. A Randomized Clinical Trial. Int. Endod. J..

[65] Wu Z., Lin Y., Xu X., Chen Z., Xiang Y., Yang L., Zhang W., Xiao S., Chen X. (2023). Clinical Observation of Autologous Platelet Rich Fibrin Assisted Revascularization of Mature Permanent Teeth. Head Face Med..

[66] Li J., Zheng L., Daraqel B., Liu J., Hu Y. (2023). Treatment Outcome of Regenerative Endodontic Procedures for Necrotic Immature and Mature Permanent Teeth: A Systematic Review and Meta-Analysis Based on Randomised Controlled Trials. Oral Health Prev. Dent..

[67] Ding Z.-Y., Tan Y., Peng Q., Zuo J., Li N. (2021). Novel Applications of Platelet Concentrates in Tissue Regeneration (Review). Exp. Ther. Med..

[68] Cecerska-Heryć E., Goszka M., Serwin N., Roszak M., Grygorcewicz B., Heryć R., Dołęgowska B. (2022). Applications of the Regenerative Capacity of Platelets in Modern Medicine. Cytokine Growth Factor. Rev..

[69] Niemczyk W., Kępa M., Żurek J., Aboud A., Skaba D., Wiench R. (2025). Comparative Evaluation of Platelet-Rich Fibrin (PRF) and Concentrated Growth Factor (CGF) as Carriers for Antibiotics—In Vitro Study. Int. J. Mol. Sci..

[70] Niemczyk W., Żurek J., Niemczyk S., Kępa M., Zięba N., Misiołek M., Wiench R. (2025). Antibiotic-Loaded Platelet-Rich Fibrin (AL-PRF) as a New Carrier for Antimicrobials: A Systematic Review of In Vitro Studies. Int. J. Mol. Sci..

[71] Hoveizi E., Naddaf H., Ahmadianfar S., Bernardi S. (2023). Using Odontoblasts Derived from Dog Endometrial Stem Cells Encapsulated in Fibrin Gel Associated with BMP-2 in a Rat Pulp-Capping Model. Curr. Issues Mol. Biol..

[72] Singh R.K., Shakya V.K., Khanna R., Singh B.P., Jindal G., Kirubakaran R., Sequeira-Byron P. (2017). Interventions for Managing Immature Permanent Teeth with Necrotic Pulps. Cochrane Database Syst. Rev..

[73] Wei X., Yang M., Yue L., Huang D., Zhou X., Wang X., Zhang Q., Qiu L., Huang Z., Wang H. (2022). Expert Consensus on Regenerative Endodontic Procedures. Int. J. Oral Sci..

[74] Law A.S. (2013). Considerations for Regeneration Procedures. J. Endod..

[75] Martin K., Nathwani S., Bunyan R. (2018). Autotransplantation of Teeth: An Evidence-Based Approach. Br. Dent. J..

[76] Patel S., Krastl G., Weiger R., Lambrechts P., Tjäderhane L., Gambarini G., Teng P. (2023). ESE Position Statement on Root Resorption. Int. Endod. J..

[77] Clauder T. (2022). Present Status and Future Directions—Managing Perforations. Int. Endod. J..

[78] Dikensoy O. (2019). Importance of Reading and Publishing Case Reports. Turk. Thorac. J..

[79] Tricco A.C., Lillie E., Zarin W., O’Brien K.K., Colquhoun H., Levac D., Moher D., Peters M.D.J., Horsley T., Weeks L. (2018). PRISMA Extension for Scoping Reviews (PRISMA-ScR): Checklist and Explanation. Ann. Intern. Med..

[80] Peters M., Godfrey C., Mcinerney P., Munn Z., Trico A., Khalil H., Aromataris E., Munn Z. (2020). Chapter 11: Scoping Reviews. JBI Manual for Evidence Synthesis.

[81] Ouzzani M., Hammady H., Fedorowicz Z., Elmagarmid A. (2016). Rayyan—A Web and Mobile App for Systematic Reviews. Syst. Rev..

[82] Booth A. (2016). Searching for Qualitative Research for Inclusion in Systematic Reviews: A Structured Methodological Review. Syst. Rev..

