# Supplementary material for: Platelet-Rich Plasma and Platelet-Rich Fibrin in Endodontics: A Scoping Review
Source: Int J Mol Sci. 2025 Jun 7;26(12):5479. doi: 10.3390/ijms26125479 (PMC12193084; doi:10.3390/ijms26125479)
Supplement: Supplementary file 1 [file ijms-26-05479-s001.zip › ijms-3652330-supplementary.pdf]

International Journal of *Molecular Sciences*

MDPI

Review

Platelet-Rich Plasma and Platelet-Rich Fibrin in Endodontics: A Scoping Review

Simão Rebimbas Guerreiro [1,*,†], Carlos Miguel Marto [2,3,4,5,6,7,*,†], Anabela Paula [3,4,5,6,7], Joana Rita de Azevedo Pereira [1], Eunice Carrilho [3,4,5,6,7], Manuel Marques-Ferreira [1,4,5,6,7] and Siri Vicente Paulo [1,4]

1 Institute of Endodontics and Laboratory of Evidence-Based and Precision Dentistry, Faculty of Medicine, University of Coimbra, 3000-075 Coimbra, Portugal; joanarapereira.md@gmail.com (J.P.); mmferreira@fmed.uc.pt (M.M.-F.); sirivicentepaulo@gmail.com (S.V.P.)

2 Institute of Experimental Pathology, Faculty of Medicine, University of Coimbra, 3000-548 Coimbra, Portugal

3 Institute of Integrated Clinical Practice and Laboratory of Evidence-Based and Precision Dentistry, Faculty of Medicine, University of Coimbra, 3000-075 Coimbra, Portugal; anabelabppaula@sapo.pt (A.P.); ecarrilho@fmed.uc.pt (E.C.)

4 Coimbra Institute for Clinical and Biomedical Research (iCBR), Area of Environment, Genetics and Oncobiology (CIMAGO), Faculty of Medicine, University of Coimbra, 3000-548 Coimbra, Portugal

5 Centre for Innovate Biomedicine and Biotechnology (CIBB), University of Coimbra, 3000-548 Coimbra, Portugal

6 Clinical Academic Center of Coimbra (CACC), 3004-561 Coimbra, Portugal

7 Centre for Mechanical Engineering, Materials and Processes (CEMMPRE), Advanced Production and Intelligent Systems (ARISE), University of Coimbra, 3030-788 Coimbra, Portugal

* Correspondence: simao.guerreiro14@gmail.com (S.G.); cmiguel.marto@uc.pt (C.M.M.)

† These authors contributed equally to this work

Academic Editor: Diana Torge

Received: 4 May 2025
Revised: 1 June 2025
Accepted: 3 June 2025
Published: 7 June 2025

Citation: Rebimbas Guerreiro, S.; Marto, C.M.; Paula, A.; Azevedo Pereira, J.R.; Carrilho, E.; Marques-Ferreira, M.; Vicente Paulo, S. Platelet-Rich Plasma and Platelet-Rich Fibrin in Endodontics: A Scoping Review. *Int. J. Mol. Sci.* **2025**, *26*, 5479. https://doi.org/10.3390/ijms26125479

Copyright: © 2025 by the authors. Submitted for possible open access publication under the terms and conditions of the Creative Commons Attribution (CC BY) license (https://creativecommons.org/licenses/by/4.0/).

Table S1. Identification of the included studies

	N	Study
**STUDY TYPE**		
**Case report**	93	[1–92]
**Narrative review**	66	[93–157]
**Randomized controlled trial**	62	[158–219]
**Randomized controlled trial protocol**	52	[220–271]
**Systematic review**	42	[272–313]
**Animal**	34	[314–347]
**Case-series**	27	[348–374]
In vitro	24	[318,375–397]
**Cohort**	5	[398–402]
**Case-control**	3	[403–405]
**Scoping review**	3	[406–408]
**Umbrella review**	2	[409,410]
**Total (Studies)**	413	

Table S2. Journals where articles on the topic were published.

Scientific Journal	N
Journal of Endodontics	55
International Endodontic Journal	20
Journal of Conservative Dentistry	19
Journal of Clinical and Diagnostic Research	17
Contemporary Clinical Dentistry	10
International Journal of Clinical Pediatric Dentistry	8
Restorative Dentistry & Endodontics	8
The journal of Contemporary Clinical Practice	7
Indian Journal of Dental Research	7
Materials – Basel	7
Dental Traumatology	6
Case Reports in Dentistry	6
BMC Oral Health	6
Applied Sciences-Basel	6
Journal of Pharmacy and Bioallied Sciences	5
Indian Journal of Public Health Research and Development	5
Giornale Italiano di endodonzia	4
Clinical Oral Investigations	4
International Journal of Clinical and Experimental Medicine	4
Nigerian Journal of Clinical Practice	3
Saudi Dental Journal	3
Platelets	3
Journal of Indian Society of Periodontology	3
Journal of the Indian Society of Pedodontics and Preventive Dentistry	3
Journal of Clinical and Experimental Dentistry	3
Australian Endodontic Journal	3

Cureus Journal of Medical Science	3
Journal of Tissue Engineering and Regenerative Medicine	3
European Endodontic Journal	3
Endo-endodontic practice	3
Frontiers in Bioengineering and Biotechnology	3
Pediatric Dental Journal	2
BMJ Case Reports	2
Medical Science	2
Brazilian Dental Journal	2
Journal of Clinical Medicine	2
Evidence-Based dentistry	2
European Archives of Paediatric Dentistry	2
The Chinese Journal of Dental Research	2
Australian Dental Journal	2
European Journal of Molecular and Clinical Medicine	2
International Journal of Pharmaceutical Research	2
Biomedical Research (India)	2
Current Stem Cell Research & Therapy	2
Journal of the Pakistan Medical Association	2
Acta Veterinaria-Belograd	2
Dental Research Journal	2
Dental Clinics of North America	2
Journal of Dentistry	2
Endodontology	2
International Journal of Clinical Pediatric Dentistry	2
Mediators of Inflammation	2
Scientific World Journal	2
International Journal of Environmental Research and Public Health	2
Journal of the Korean Academy of Pediatric Dentistry	1
Journal of Health and Allied Sciences NU	1
Clinical Case Reports	1

Indian Journal of Pharmaceutical Sciences	1
Experimental and Therapeutic Medicine	1
International Journal of Paediatric Dentistry	1
Journal of Pharmaceutical Negative Results	1
Journal of Pharmaceutical Sciences Research	1
Pediatric Dentistry	1
Periodontology 2000	1
Cell and Tissue Research	1
Advances in Clinical and Experimental Medicine	1
Kathmandu University Medical Journal	1
Journal of Oral and Maxillofacial Pathology	1
Medicine and Health-Kuala Lumpur	1
Journal of Istanbul University Faculty of Dentistry	1
European Journal of Dentistry	1
European Cells and Materials	1
Journal of International Medical Research	1
Cell Journal	1
Bioinformation	1
National Journal of Maxillofacial Surgery	1
Healthcare – Basel	1
Bioactive Materials	1
Dental and Medical Problems	1
Journal of Cranio-Maxillofacial Surgery	1
Dental Hypotheses	1
Journal of the Liaquat University of Medical and Health Sciences	1
Macedonian Journal of Medical Sciences	1
F1000Reseach	1
Journal of International Oral Health	1
Cytokine and Growth Factor Review	1
Iranian Endodontic Journal	1
Pharmaceuticals – Basel	1

Current Therapeutic Research	1
Research Journal of Pharmacy and Technology	1
World Journal of Clinical Cases	1
Journal of the Formosan Medical Association	1
Journal of Advanced Oral Research	1
BMJ Open	1
Today´s FDA	1
Scientific Reports	1
Journal of Periodontal and Implant Science	1
Journal of Clinical and Translational Research	1
General Dentistry	1
Biomimetics	1
Archives of Oral Biology	1
Experimental and Toxicologic Pathology	1
International Journal of Surgery Case Reports	1
Annals of Anatomy	1
Journal of Research in Medical and Dental Science	1
Head and Face Medicine	1
Journal of Natural Science, Biology and Medicine	1
Stem Cells International	1
Molecular Biology Reports	1
International Journal of Experimental Pathology	1
Medicina Clinica Pratica	1
Journal of Veterinary Dentistry	1
Indonesian Biomedical Journal	1
Indian Journal of Forensic Medicine and Toxicology	1
Journal of Dental Science	1
Frontiers in Dental Medicine	1
Current Issues in Pharmacy and Medical Sciences	1
Journal of the International Society of Preventive and Community Dentistry	1
Journal of Personalized Medicine	1

International Journal of Oral Science	1
Oral health & Preventive Dentistry	1
International Journal of Pharmaceutical and Clinical Research	1
Minerva Stomatology	1
Indian Drugs	1
Journal of Operative Dentistry & Endodontics	1
Biomedical Journal of Scientific & Technical Research	1
International Journal of Current Research	1
Journal of dental sciences and research	1
Journal of Interdisciplinary Dentistry	1
Advances in Bioresearch	1
Journal of Dental and Medical Sciences	1
International Journal of Prosthodontics and Restorative Dentistry	1

Table S3. Pulpotomy/Pulp capping studies.

Author & year	Study type	Sample Size	Sample characteristic	Pulp/PA diagnosis	Study Protocol	Platelet concentrate used and protocol	Procedure	Treatment outcome	Follow up
**Garg S., *et al.*, 2023** [261]	RCT protocol	N=30 NR	Mandibular first and second permanent molars.	Irreversible pulpitis. Exposed pulp of permanent molars w/ symptomatic pulpitis, w/ no swelling, no chronic lesion, mobility nor periodontal involvement, or long history of pain. PA widening (mild) may or may not be present. Sensitivity to hot and cold, acute pain and tenderness on percussion	Group A- Biodentine. Pulpotomy of permanent teeth. Application of a 2mm thickness Biodentine material over the pulp. Control Intervention- PRF and Biodentine: Pulpotomy of permanent teeth and application of PRF. Application of a 2mm thickness Biodentine material over the pulp.	PRF	Pulpotomy + pulp capping	Primary Outcomes: Clinical Evaluation - Absence of symptomatology: pain, swelling, tenderness on percussion, mobility. Radiographic Assessment- Absence of evident pathosis, for instance, RR, furcal pathosis, new PA lesion, and/or resolution of apical widening. PT- Positive response. Secondary Outcomes: Clinical Evaluation - Absence of symptomatology: pain, swelling, tenderness on percussion, mobility. Radiographic Assessment- Absence of evident pathosis, for instance, RR, furcal pathosis, new PA lesion, and/or resolution of apical widening. PT- Positive response.	Main outcomes follow-up: 1, 3, 6 months Secondary outcomes follow up: 8, 12 months

				may or may not be present.					
**Singla M. *et al.*, 2023** [411]	RCT protocol	N=48 NR	25–45-year-old patients	Irreversible pulpitis. Curiously exposed pulp of permanent molars with mature apex, with symptoms of IP and positive response to vitality tests,	Intervention 1: Pulpotomy using PRF and Biodentine VS, Pulpotomy using PRF, Biodentine, and Collaplug. Control Intervention 1: Pulpotomy using Biodentine	PRF In the form of a membrane.	Pulpotomy + pulp capping	Main outcomes: Spontaneous pain, sensitivity, and tenderness to percussion Secondary outcomes: PA index compared to baseline using radiographic evaluation.	Follow-up main outcomes: 1 day 2 days, and 7 days Follow-up secondary outcomes: 3, 6, and 12 months

				with no radiographic lesion visible.					
**Eid *et al.*, *2022*** [177]	RCT	N=63 teeth<br><br>Grupo A (PRF): 21<br>Grupo B (MTA): 21<br>Grupo C (NHA): 21	Boys and girls between 6-12 years-old	Vital	Unique session, if pulp hemostasis was not achievable w/in 5 minutes the tooth was excluded from the study.<br>Patients returned 6 and 12 months later for evaluation.	PRF<br>5 mL of venous blood was collected in a 10 mL glass tube w/out anticoagulant and then centrifugated using a tabletop centrifuge (Hettich, Hohberg, Germany) at 3,000 rpm for 10 minutes.<br>The PRF clot was then dried w/ sterile gauze for it to become a membrane	Pulpotomy	In all three groups was observed radicular formation, including the complete closure of the open apex.<br>Furthermore, MTA and NHA showed a higher tendency to promote pulp obliteration than PRF.	6 and 12 months

**Mandviwala *et al.*, 2022** [51]	Case report	N=1 tooth	25 years-old female w/ pain on #46 tooth for 1 month. The pain was dull and aggravated w/ cold. The tooth was not tender to percussion and had a positive response to PT.	Symptomatic IP, No PA lesion was visible in the radiography.	Rubber dam isolation, remaining caries excavation, and partial coronal pulp removal were done using a Sterile Round Diamond Bur and irrigation. Hemostasis was achieved w/in 5 min using saline-soaked cotton pellets, after which a small piece of PRF was used to cover the pulpal wound, and a 2-mm thick layer of Biodentine was placed over it.	PRF 302×g for 10 minutes	Pulpotomy + pulp capping	At 6 months follow up a radiolucent lesion in the third of the mesial root and in the PA area was observed. CBCT was performed to evaluate the type of RR and confirmed an Internal RR. Diagnosis of PA periodontitis w/ RR was made and a multi-visit Root canal treatment was made, w/ filling of the RR w/ MTA and gutta percha obturation.	A telephonic follow-up was carried out after 48 h, 7 days, and 3 months. Follow-up of the Pulpotomy treatment was for 6 months, while the follow-up of the root canal treatment and RR management w/ Biodentine was performed after one year.
**Mohammed *et al.*, 2022** [196]	RCT	N=98 teeth<br>Group A: PRF and MTA<br>Group B: PRF and Theracal	Patients aged between 17-50 years old, healthy males and females.	Symptomatic and asymptomatic pulpitis	Local anesthesia, rubber dam isolation, caries removal w/ spherical bur and access cavity, and amputation of the pulp by the pulp pavement floor level. Hemostasis w/ cotton and saline for 5 minutes. PRF was laid on the pulp tissue, and MTA or TheraCal was inserted. Access cavity restoration.	PRF 10ml of venous blood w/out centrifuged at 3,000rpm for 12 minutes PRF was collected and squeezed into a membrane	Pulpotomy	Clinical success: absence of pain, swelling, and no tenderness to percussion; radiographically success: absence of widening in the lamina dura, periapical radiolucency, or furcal involvement after 12 months. At 6 months follow-up, the success rate was significantly higher in MTA+PRF and at 1 year follow up MTA+PRF showed more dentin bridge formation and positive vitality	3, 6 and 12 months

							test for 15 teeth in the MTA group. Success of 75% and 71.43% for each group.

RCT- Randomized controlled Trial; W/- With; PRF- Platelet-rich-Fibrin; PRP- Platelet-rich-plasma; PT- Pulp tests; RR- Root resorption; PA- Periapical; ICM- Intracanal medication; RPM- revolutions *per* minute; IP- Irreversible pulpitis; MTA- Mineral trioxide aggregate; CBCT- Cone-beam computerized tomography; NHA- Nano-hydroxyapatite; NR – Not reported.

Table S4. Regenerative endodontic procedures in immature teeth studies.

Author & year	Study type	Sample Size	Sample characteristic	Pulp/PA diagnosis	Study Protocol	Platelet concentrate used and protocol	Procedure	Treatment outcome	Follow up
**Prakash *et al.*, 2023** [199]	RCT	N=20 teeth	Children from 7-9 years old	Immature necrotic young permanent teeth w/ a radiotransparent lesion w/ less than 10 mm and apical foramen w/ 1 mm or more. No signs of RR.	First visit: Anesthesia and isolation, access cavity, and irrigation protocol of 1.5% NaOCl 20 mL *per* canal for 5 minutes. Canals were dried, access cavity was sealed. Second Visit (4 weeks later): Local anesthesia, irrigation w/ saline, and 20 ml of 17% EDTA. PRF Group: the PRF membrane was cut into small pieces, loaded into a spinal needle, and condensed into the canal. BC Group: blood clot was formed till the CEJ level. Bleeding was induced w/ a 15 k-file 2 mm beyond the apex. The BC was covered w/ a collagen plug. In both groups, the cavity was sealed w/ 3-4 mm of MTA and GIC.	PRF 10 mL of venous blood was withdrawn from the patient and centrifuged at 3,000 rpm for 10 minutes. The PRF clot was then separated from the other layers and compressed w/ sterile gauze to obtain a PRF membrane.	REP of immature permanent teeth with pulp necrosis (following the Current American Academy of Endodontics -AAE clinical considerations)	Clinical outcome: both groups showed a 100% resolution of the clinical symptoms. There was a clear increase in PA healing but w/ no significant difference between the two groups. There was a positive significant statistical difference of PRF over the BC group related to the thickness of dentin walls at baseline and after 6 months. In both the PRF and BC groups a statistically significant increase of root length and dentin thickness when comparing the baseline radiography to the 6 months follow-up was noted. In the PRF group, there was a positive significant difference noted with the apical response scores at 1, 3, and 6 months.	1, 3 and 6 months

**Li *et al.*, 2023** [412]	Case-Control Study	N=13 teeth<br><br>CGF group=7 teeth<br>PRF group=6 teeth	Patients with 6–16-year-old	Necrotic permanent immature teeth w/ a PAI less than 3-4 and a Nola development of at least 7-9 stages	First visit: Access cavity performed under isolation w/ round bur. RC disinfected with 10-20mL of 1-3% NaOCl w/ minimal mechanical preparation. ICM (Calcium hydroxide) was inserted in the dry canal and the access cavity was sealed.<br>Second visit (2 weeks later): removal of temporary restoration and rinse of ICM using saline and ultrasound methods. Final irrigation protocol using 10-15 mL of 17% EDTA. The canal was dried, and PRF/CGF was placed gently in the RC up to the CEJ level. Bioceramix was placed over the PRF to form a plug. Access cavity restored.	PRF<br>10 mL of venous blood was drawn into a 10 mL tube without coagulant, and centrifugated in a DT-F4 (Chengdu Dengtuo Medical Instrument Co., LTD) for 3,000 rpm for 10 minutes at room temperature. Three layers formed, the middle layer (PRF clot) was separated and put into a box-shaped separator to make it a membrane. It was then trimmed to a suitable size to better fit the RC.	REP of immature permanent teeth with pulp necrosis	The outcomes evaluated (root length increase, apical foramen closure, reduction of PA lesion, and the increased rate of radiographic root area) were compared to preoperative radiographs.<br>At the 3-6 months follow-up: The success rate of RET was 100% and there was no significant difference between CGF and PRF in the outcomes.<br>At the 6-12 months follow-up: No clinical symptoms in any tooth, and no calcification was reported. At the 12-24 months follow-up: The increase rate of radiographic root area of the PRF group was statistically significantly higher than the CGF group.	First follow-up: 3-6 months<br>Second follow-up: 6-12 months<br>Third follow-up: 12-24 months

**Das *et al.*, 2023** [16]	Case Report	N=1 tooth	A 7-year-old male patient w/ complains of pain in the lower jaw for 20 days. Pain was sudden onset, moderate to severe in intensity.	Immature mandibular permanent first molar w/ IP; deep occlusal caries tender on percussion; incomplete root formation and increased periodontal ligament space	REP was carried out in 2 visits. In the first visit, the decay was removed, and canal pulp extirpation and irrigation w/ 1.5% NaOCl was done. Saline irrigation was done to reduce cytotoxicity to stem cells, the canals were dried, and a layer of TAP was applied (0.1-1.0mh/mL, *ratio* 1/1/1 ciprofloxacin, metronidazole, and minocycline). The tooth was sealed with GIC. After 4 weeks, PRF was obtained; the RC was irrigated w/ 20 mL of 17% EDTA. The PRF membrane was cut into small pieces and condensed in each canal. Bleeding was stopped at a point where 3-4 mm of restorative material could be inserted. PRF was covered by a layer of MTA, and then a 3-4 mm layer of GIC and a composite resin restoration.	PRF 10 mL of blood was drawn from the patient's left forearm and centrifuged at 2,700 rpm for 12 minutes to obtain a PRF membrane	REP on Immature mandibular permanent first molar with IP	No pain nor discomfort to percussion or palpation after 3 months. The tooth was asymptomatic and did not react to cold or electric PT. An Increase in the root apex formation after 6 months of follow-up was observed.	every three months (radiographic and clinical examination)

**Rios-Osorio *et al.*, 2023** [303]	Systematic review	10 RCTs were included<br><br>N= 352 teeth PRF Group:73 PRP Group:113 BC group: 166	Patients ranged from 7 to 54 years w/ symptoms related to pulp necrosis such as pain, abscess, fistula, and tenderness to percussion and palpation	Immature permanent teeth w/ necrotic pulp w/ or w/out PA lesion	The protocols for revascularization of immature teeth were the same among the included studies. All used 1%-5.25% NaOCl as the main irrigant agent, and 3 reported the use of 17% EDTA irrigation as the final irrigant. All included RCTs used ICM in the form of TAP or DAP (Metronidazole, ciprofloxacin, minocycline, clindamycin)	PRF & PRP	REP in Immature permanent teeth w/ necrotic pulp	Of the 73 teeth treated w/ PRF, 1 failed, and the other 72 were considered clinical success, w/ an increase in root growth and maturation, resolution of fistula/abscess, pain, and PA lesion. In the PRP Group, of the 113 included teeth, 5 cases failed, and the remaining 105 showed total clinical resolution.<br>According to 1 of the 10 included studies, PRF showed a statistically significant difference over BC In root length growth, apical closure, and increased PA bone density, whereas 3 RCTs showed a statistically significant difference in PRP over BC in root length growth and dentin wall thickness. However, 1 RCT reported that the BC group promoted a better increase in the root area when compared to PRP	between 12 andasd 28 months

**Kumar *et al.*, 2023** [50]	Case Report	1	8-year-old female w/ pain in the upper front tooth region for 1 week History of trauma six months ago	Traumatized non-vital young permanent incisor w/ a fracture involving the pulp w/ blunderbuss canals. No response to the electric PT	Treatment was made in 2 visits. First visit: Isolation w/ rubber dam and access opening without bleeding occurred. Minimal instrumentation was done. Irrigation w/ 0.09% saline (20mL). The canals were dried using sterile paper points. TAP was placed, and the canal was sealed w/ zinc oxide eugenol cement. Second visit (after 2 weeks):TAP was removed w/ irrigation (saline), bleeding was induced and PRF was inserted into the RC. The canal was sealed w/ MTA, a cotton pellet, and zinc oxide eugenol cement for temporary restoration. After 24 hours the patient was recalled, the pellet was removed and type II GIC was placed. The final restoration was made in composite.	PRF PRF was prepared "as *per* standard preparation protocol" Squeezed into a sterile gauze to become a membrane.	Revascularization in a non-vital traumatized immature permanent incisor	Root length formation, increase in dentin thickness, and root end closure were assessed in the radiographic controls at 6 and 9 months. After 12 months, the tooth was asymptomatic, presenting increased dentinal thickness and lengthening, and apex closure. CBCT confirmed the apical closure had occurred. Pulp canal obliteration was noticed at the cervical third of the RC at the 12th month of follow-up.	6, 9, and 12 months (clinical and radiographic evaluation).

RCT- Randomized controlled Trial; W/-With; PRF- Platelet-rich-Fibrin; PRP- Platelet-rich-plasma; PT- Pulp tests; RR- Root resorption; GIC- Glass ionomer cement; REP- Regenerative endodontic procedure; CGF- concentrated grown factors; PAI- Periapical index; PA- Periapical; ICM- Intracanal medication; CEJ- Cement enamel junction; RC- Root-canal; RPM- revolutions *per* minute; BC- Blood clot; IP- Irreversible pulpitis; TAP- Tri-antibiotic paste; DAP- Double-antibiotic paste; MTA- Mineral trioxide aggregate

Table S5. Apexification studies.

Author & year	Study type	Sample size	Sample characteristics	Pulp/PA diagnosis	Study Protocol	Platelet concentrate used and protocol	Procedure	Treatment outcome	Follow-up
**Biradar *et al.*, 2023** [354]	Case series	N=3 patients	Case1: 11-year-old girl w/ swelling in the upper front tooth #11 and #12, spontaneous, throbbing, localized, radiating pain aggravated w/ hot, cold and sleeping.	Chronic PA abscess in relation to #11 and #12. A poor RC treatment was present at tooth #11 w/ a large radiolucency in the PA area of #11 and #12. No positive response to PT, suggestive of non-vitality of #12.	First visit: Access cavity followed by no instrumentation but repeated gentle irrigation w/ 1.5% NaOCl and saline. The RC was dried with sterile absorbent paper points. RC was filled w/ TAP (ciprofloxacin+metronidazole +minocycline) mixed w/ propyleneglycol below the CEJ. Second visit (after 6 weeks): anesthetic procedures, temporary restoration removed, and irrigation w/ 17% EDTA was done. The PRF membrane was inserted w/ the help of an endodontic plugger gently in the apical part of the root apex. A permanent restoration was done w/ GIC for a better seal. 12 months after treatment, as there was no significant	PRF 10 mL of blood from the medial cubital vein of the patient was collected into a tube w/ no anticoagulant and centrifugated at a frequency of 3,600 rpm for 10 minutes. After, the PRF clot was separated from the other components and squeezed into a sterile gauze to obtain a PRF membrane	Apexification of open apex teeth	Case 1: after 12 months there was still no apical closure, therefore the tooth had to undergo apexification w/ an apical MTA plug. Cases 2 and 3: there was apical closure by the time of the first follow-up consult (6 months after the procedure), and were considered clinical success	6 and 12 months

		increase in the thickness of the wall of the root, the canal was accessed and was filled apically with 3 mm of MTA and the rest with Gutta-Percha.
Case 2: 9-year-old boy; pain in the left upper front tooth for a few months. The pain was spontaneous, throbbing, localized, radiating, and aggravated w/ hot, cold, and sleeping.	Acute PA abscess in need of endodontic therapy w/ apical closure. PA radiograph showed a fractured tooth involving the pulp and PA radiolucency	The clinical protocol was the same as for the first case, except PRF was placed at the end of 2 weeks and the tooth was obturated after 6 months as there was apical closure

				suggestive of IP in relation to #21					
			Case 3: 13-year-old boy w/ chief complain of pain in his upper front teeth for a few months. Pain was spontaneous, dull, localized aggravating w/ cold and hot and while sleeping.	IP PA radiography showed a fracture of the tooth involving the pulp, suggestive of IP.	The clinical protocol was the same as the previous 2 cases, except, the tooth was obturated at the end of 3 months w/ an apical Biodentine plug and gutta-percha obturation				
**Saxena *et al.*, 2022** [305]	Systematic Review	14 articles were selected: 4 were RCT, 7 controlled clinical trials and 3 case reports) Of the included studies: 32 patients were	Children between 6-14 years old	NR	N/A	PRF & PRP	REP in pulpal-involved teeth	PRF Groups: 100% clinical success in the studies that used PRF as regenerative material. Patients were asymptomatic; however, the success rate was 94.73% at 18 months follow-up, and a few teeth had pain and mobility. A 94.73% radiographic success rate was observed at 12 months. PRF showed great results. At 18 months 98% of teeth showed PA healing, 40 %	From 12 to 18 months

		treated w/ PRF and 25 w/ PRP						showed apical closure, 99% showed root lengthening and 60% showed dentinal thickness.<br>PRP groups: 100% clinical and radiographic success. However, at 18 months PRP showed PA radiolucency whereas root lengthening and dentinal wall thickness were achieved among 40 and 20% of the patients, respectively.<br>The reason cited for less success rate in the PRP group was lesser healing kinetics on osteoblasts that led to leaching out of the growth factors.<br>In the REP procedures, among all the tested scaffolds, PRP and PRF showed better results.	
**Pruthi *et al.*, 2020** [366]	Case series	N=2 teeth	17-year-old girl w/ pain and discoloration of her maxillary anterior teeth. History of trauma 10 years prior.	Maxillary central incisors diagnosis with pulp necrosis w/ chronic PA periodontitis	First visit: teeth isolation and access opening. Working length was determined, the canal was mechanically debrided and prepared w/ an 80K-file; irrigation with 3% NaOCl. Ca(OH)2 paste was applied for 1 week as ICM	PRF<br>8 mL of venous blood was collected in a 10 mL glass tube w/ no anticoagulant and centrifugated for 10 minutes at 3,000 rpm. The	Apexification of central incisors w/ Biodentine and PRF as an apical matrix	The patient was asymptomatic in every follow-up visit; teeth were functional and PA lesions showed remarkable healing at the 3-year follow-up.	every 6 months until 3 years after treatment

					and a 3 mm thick temporary restoration was placed. Second visit: the patient was asymptomatic, removal of the ICM. Irrigation w/ 3% NaOCl, 17% EDTA, and saline, and after, the RC was dried. PRF was placed as an apical matrix in the PA area of each incisor. Biodentine was mixed and condensed against the PRF matrix until half the root length. The rest of the RC was filled w/ gutta percha and AH Plus sealer. The cavity was sealed w/ nanohybrid composite resin.	PRF clot was collected and squeezed in sterile gauze to remove excess fluid.			
**Murray *et al.*, 2018** [295]	Systematic Review	222 immature teeth 94 treated w/ PRP, 54 w/ PRF, and 74 w/ BC	Children and adults 6-28 years old	Necrotic pulp of immature permanent teeth	Procedure varies from study to study, regarding NaOCl irrigation concentration and the type of ICM used.	PRF & PRP	Apical closure	The success rate for apical closure or reduction after 1 year was: PRP (85.1%), PRF (85.2%), and BC (58.8%). The success rate for root lengthening after 1 year was: BC (64.1%), PRP (64.2%), and PRF (74.1%). PA lesion healing response was 88.9% for BC, 100% for PRP, and 100% for PRF. Dentinal wall thickening was 100% for BC,	12 months

								100% for PRP, and 100% for PRF. Apical closure occurred more frequently w/ PRP and PRF than w/ BC	
**Santhakumar *et al.*, 2018** [207]	RCT	PRF membrane group: 20 patients. PRF gel group: 20 patients.	7-12 years old Root length less than crown length, open apex w/ more than 2mm wide	Non vital maxillary central incisors	First visit: isolation, access opening, and non-vital pulp were removed. Irrigation was done w/ 3% NaOCl and saline. TAP was applied and temporary restoration was made. Second visit (21 days later): TAP was removed w/ saline irrigation. According to each group, a PRF membrane or gel was applied. Access was sealed w/ MTA, type II GIC, and composite material.	PRF 5 mL of venous blood was collected w/out anticoagulant and centrifuged at 3,000 rpm for 10 minutes. PRF membrane was done by squeezing PRF gel/clot between sterile gauzes.	Apexification	The patients were reassessed for clinical and radiographic success at 6, 12, and 18 months. PRF gel gave better radiographic success in a 12-month follow-up period. However, in the clinical assessment, PRF membrane and gel had the same success. REP w/ PRF is easier and less time-consuming because it facilitates the scaffold manipulation	18 months

RCT- Randomized controlled Trial; W/-With; PRF-Platelet-rich-Fibrin; PRP-Platelet-rich-plasma; PT-Pulp tests; RR-Root resorption; GIC- Glass ionomer cement; REP-Regenerative endodontic procedure; PA-Periapical; ICM- Intracanal medication; CEJ- Cement enamel junction; RC- Root-canal; RPM- revolutions per minute; BC- Blood clot; IP- Irreversible pulpitis; TAP-Tri-antibiotic paste; DAP-Double-antibiotic paste; MTA- Mineral trioxide aggregate; N/A-Not applicable; NR-Not reported.

Table S6. Apical surgery studies

Author & year	Study type	Sample size	Sample characteristics	Pulp/PA diagnosis	Study Protocol	Platelet concentrate used and protocol	Procedure	Treatment outcome	Follow-up
**di Lauro *et al.*, 2023** [284]	Systematic Review	14 studies included: 9 RCTs and 5 non-randomized clinical controlled trials.	NR	Persistent PA lesions	In the included studies RES and preparation were performed, and, after the PA lesion was removed, the bone defect was filled w/ materials that varied from study to study: PRF, PRP collagen membrane, hydroxyapatite, PRGF. The retrograde filling of the root end was made w/ Biodentine or MTA.	PRF & PRP	RES	4 studies evaluated only clinical outcomes; 4 studies analyzed the PA bone healing; 6 studies analyzed both outcomes. Clinical and radiographical findings showed that when PRF and PRP were used, patients exhibited less pain and swelling and a greater reduction of PA radiolucency after 12 months of follow-up on average	1 week to 12 months
**Sinha *et al.*, 2023** [308]	Systematic review with meta-analysis	10 RCTs were included in this review<br><br>421 PA lesions in total: 187 received PRF treatment and 234	NR	Teeth with PA lesion	N/A	PRF	Apical surgery	Outcomes: post-op pain; quality of life; PA bone healing.<br>2 studies analyzed the post-op pain, w/significantly lower pain in PRF groups than in control groups. 2 studies evaluated the bone density by CBCT: no statistically significant difference existed between the PRF and the control groups. Qualitative assessment of bone healing	From 1 week to 1 year

		were controls						by PA radiography showed no statistically significant difference between PRF and the control groups. However, quantitative assessment in two studies showed a significantly higher rate of healing after 9 months in the PRF group. Results suggest a reduction of pain post-op when PRF was used	
**Govindaraju *et al.*, 2023** [28]	Case Report	1	38-year-old male w/ pain and mild swelling in the upper front tooth region.	CBCT imaging showed a radiolucent PA lesion in relation to the right maxillary central and lateral incisor. The lesion had a 12 mm larger axis. History of trauma 15 years before.	2 visits were made for conventional endodontic treatment: first, access opening and working length determination were performed. Next, cleaning and shaping w/ K-files was done up to 80k file (being the master apical file 50k) and using a step back procedure. For irrigation, 5.25% NaOCl was used, followed by 17% EDTA gel. As final irrigation saline was used. $Ca(OH)2$ was used as ICM, placed twice inside the canal w/ a one-week interval, but the patient was still symptomatic. Obturation was done w/ gutta-percha and AH Plus sealer.	PRF 10 mL of venous blood was drawn from the patient and collected in two sterile vacutainer tubes of 5 mL each w/out anticoagulant and centrifuged at 3.000 rpm for 10 minutes. The middle layer containing the fibrin clot was collected by dividing the red blood cells and plasma. The junction layer of cellular plasma	RES	No symptoms such as pain, inflammation, or discomfort were reported nor observed in the follow-up of the case. Biopsy result: radicular cyst	3,6, and 9 months

					PA surgery was planned. Surgery was done under general anesthesia since the doctors wanted also to extract a supernumerary tooth and perform the cyst enucleation. Local anesthesia was also performed. A full-thickness mucoperiosteal flap was raised and the supernumerary tooth was extracted by palatal approach followed by cyst enucleation on the labial side. Biopsy was taken for analysis and RES w/ retrograde filling was made w/ MTA. PRF was placed in the cyst cavity and suturing was done.	and the fibrin was carefully preserved as it contains high platelet concentration.			
**Alsolaihim *et al.*, 2023** [106]	Narrative Review	N/A	N/A	Teeth with PA lesions	N/A	PRF & PRP	Review of materials used in clinical trials during RES	PRF + B-TCP: widely used in endodontic surgery to enhance bone formation. It's bioactive and fully resorbed w/ the release of osteoinductive elements. PRF: demonstrates superior PA tissue regeneration and healing.	Minimum 3-month

								PRP + TPC: provided satisfactory results on PA regeneration.<br>PRF + Hydroxyapatite: HA has shown to be a good scaffold, bonding directly to the existing bone, and it augments the regenerative effects seen in PRF.	
**Naga *et al.*, 2023** [256]	RCT protocol	N=20 patients	Mandibular first molars 18–45-year-old males	Non-vital teeth w/ iatrogenic errors in the 3 mm apical portion of the root	Piezosurgery or trephine bur will be used to access the apical portion of the root and to do the root end resection. Curettage of the lesions and in 2 groups PRF was placed in the bone defect and in the other 2 groups, BC served as scaffold.	PRF	RES	N/A	Pain and swelling accessed for five days every 24, 48-, 72-, 96-, and 120-hours post-op.

RCT- Randomized controlled Trial; W/-With; PRF-Platelet-rich-Fibrin; PRP-Platelet-rich-plasma; PT-Pulp tests; RR-Root resorption; GIC- Glass ionomer cement; REP-Regenerative endodontic procedure; PA-Periapical; ICM- Intracanal medication; CEJ- Cement enamel junction; RC- Root-canal; RPM revolutions per minute; BC- Blood clot; IP- Irreversible pulpitis; TAP-Tri-antibiotic paste; DAP-Double-antibiotic paste; RES- Root end Surgery; B-TCP- betatricalcium phosphate; MTA- Mineral trioxide aggregate; N/A-Not applicable; NR-Not reported

Table S7. Reimplantation of avulsed teeth and intentional reimplantation studies.

Author & year	Study type	Sample size	Sample characteristic	Pulp/PA Diagnosis	Study protocol	Platelet concentrate used and protocol	Procedure	Treatment outcome	Follow-up
**Yang *et al.*, 2023** [373]	Case series	N=2	Case 1: 14-year-old boy w/ avulsion of #21, 18h before presenting to the hospital<br><br>Case 2: 17-year-old boy w/ avulsion of #22, 2h before presenting to the hospital.	Avulsion	Case 1: the PRF clot was cut into small granules and the tooth was reimplanted alongside the PRF granules. The teeth were then splinted using a titanium labial arch for 4 weeks. The tooth was sensitive to percussion and w/ a small shadow in the PA region after 2 weeks, so RC therapy was performed. $Ca(OH)_2$ paste was used as ICM for 4 weeks. Obturation was done w/ hot-melt gutta percha and restoration w/ composite.<br><br>Case 2: tooth #22 was reimplanted w/ the PRF granules and splinted for 2 weeks. After pulp vitality assessment, the RC treatment was done w/ $Ca(OH)_2$ being the ICM for 4 weeks. Obturation was done w/ biotype RC filling sealer	PRF<br>10 mL of venous blood was drawn to a glass tube w/out anticoagulant and centrifugated at 400×g for 10 minutes. The fibrin clot was separated from the rest of the layers. The clot was squeezed in a sterile gauze to become a membrane.	Reimplantation	The reimplanted teeth showed no symptoms of inflammatory RR nor ankylosis after the treatment follow-up of 3, 6, and 12 months.<br>In neither tooth periodontal pockets, tooth discoloration or swelling were observed and, in both, a continuous PDL space was radiographic visible and no sign of resorption.	3, 6, and 12 months

					and hot-melt gutta-percha. The final restoration was done w/ composite.				
**Khurshid *et al.*, 2022** [287]	Systematic Review	10 case reports; 3 animal studies, 1 *in vitro* and 1 *in vitro* and animal study<br><br>N=10 teeth in the case reports; N=116 teeth in the animal study	Case reports: 8 male, 2 female; age 11-45.<br><br>Animal studies: 2 studies used adult mongrel dogs' teeth, the other didn't report the dogs' race.	Avulsed teeth	In 4 case reports, PRF was used in the alveolar socket, and in another, PRF was used intracanallary.<br>In 2 reports PRP was applied in the socket; in two others was used in the RC and, in one study, was mixed w/ HA and placed in the socket.<br><br>Animal studies: in two studies PRP and PRF were applied in the socket; in the other, PRP was applied intracanallary.<br><br>*In vitro* studies: hPDLSCs were cultured in PRF. In the other study hPDLCs on 30 extracted teeth were treated w/ PRF after a 40 min dry time	PRF & PRP	Tooth reimplantation	Case reports: PRP and PRF reduced the mobility to grade I from grade III in 12 months and new alveolar bone formation was enhanced. There was a reduction of the lesions' radiolucency.<br><br>Animal studies: PRP w/ calcium chloride group did not exhibit RR in one study; in another, PRP reduced ankylosis and formed more tissue than the control groups. In the last one, the PRF group showed less inflammatory RR<br><br>*In vitro* studies: PRF stimulated higher hPDLSCs proliferation, bone regeneration, and PDL-like tissue formation.	5.5 months to 2 years
**Parthasarathy *et al.*, 2022** [63]	Case Report	N=2 teeth	29-year-old male victim of a traumatic accident in the upper front tooth region	Teeth #11 and #12 avulsed w/ no fractures	RC therapy was performed in both teeth extra orally. Access opening and pulp extirpation were done, and the canal was cleaned and disinfected w/ EDTA, saline,	PRF<br>10 mL of the patient venous blood was collected and centrifugated to	Extraoral RC treatment followed by Reimplantation	After 2 weeks, a loss of labial cortex was evident in the #11 and #12 region. Periodontal examination showed a CAL of 10 mm and regenerative periodontal surgery was	2 weeks, 4 weeks, 3, 6 and 12 months

					and povidone iodine irrigation. Rotary Instrumentation (Protaper gold) was used for shaping the canal. ICM was done with $Ca(OH)_2$ and the access opening was sealed w/ GIC. 1.23% acidulated phosphate fluoride was used for 15 minutes to treat the root surface. PRF membrane was wrapped around the root surfaces. The sockets were debrided w/povidone-iodine w/out removing the coagulum. The teeth were then reimplanted w/ gentle digital pressure and were secured w/ a wire and composite splint for 2 weeks. Antibiotics were prescribed. After 2 weeks, the obturation was done w/ gutta-percha, and the access cavity was sealed w/ composite.	obtain a PRF clot which was then pressed against a sterile gauze to become a membrane.		realized. After periodontal treatment, the splint stayed for more 5 weeks. The splint was then removed w/ a positive periodontal outcome. The patient was followed for a year postop and PA radiography showed healthy and intact PA structures and PDL.	
**Yang *et al.,* 2021** [92]	Case Report	N=1 tooth	20-year-old male w/ swelling in the left maxillary premolar region w/ slight discoloration but no decay. Not	Mature maxillary premolar w/ Sinus tract and fracture at the occlusal	First, RC debridement and disinfection were done. After 14 days of the RC procedure, the inflammation had been mitigated and the sinus tract disappeared but	PRF 20 mL of venous blood was collected from the median cubital vein of the patient	RC treatment followed by extraoral retrograde filling and	At the 2-week follow-up, the splint was removed. No clinical symptoms such as pain, swelling, or exudates were present, while the	2 weeks, 1 month, 6 months, 12 months and 24 months

		tender to percussion or palpation, no positive response to PT. The tooth had a normal PD.	surface of the tooth. Large radiolucency area within the middle third of the root Diagnostic: Internal RR and root fracture	bleeding exudates from the perforation of the pulp cavity was still present. Intentional reimplantation was performed: the tooth was removed and the apical third was visibly fractured. The most apical part of the tooth was cleaned. The apical lesion was removed, and curettage of the apical part of the socket was done w/out touching the walls of the socket, to preserve the PDL, which was then irrigated w/ saline and the cavity of the lesion filled w/ an L-PRF clot. The extraoral manipulation included RC cleaning and reverse obturation. The canal was irrigated w/ 2.5% NaOCl followed by saline to remove remaining $Ca(OH)_2$. Reverse RC obturation w/ MTA was done up to the CEJ and GIC was used for restoration. After the extraoral manipulation, the tooth was replanted. The whole		Intentional replantation	replanted tooth showed class II mobility. At 4 weeks, the tooth presented class I mobility and a decrease in the radiolucency area around the tooth. At 6 months the patient had no symptoms, no mobility no metallic sound on percussion or pathologic PD. At 12 and 24 months, the radiographic examination showed no ankylosis, no RR, and a great resolution of the lesion.

					procedure took less than 15 minutes.<br>The tooth was splinted for 2 weeks				
**Behnaz *et al.*, 2021** [317]	Animal Study	Split mouth design<br>2 male 12-14 months beagle dogs<br><br>N=16 teeth<br>Control group: 8<br>PRF group: 8	Fully erupted w/ no pulpal, PA, or periodontal involvement and complete root formation	Avulsion	Atraumatic extraction of the maxillary and mandibular incisors and first premolars in both quadrants.<br>Extraoral RC therapy was performed: access cavity and instrumentation were carried out, followed by obturation w/ gutta-percha and AH26 sealer. The teeth were kept dried for a total of 60 minutes prior to replantation.<br>PRF was placed in the extraction socket of the experimental group.<br>Reimplantation was done w/ digital pressure, and the teeth were splinted to the adjacent teeth for 2 weeks.<br>Splints were removed 2 weeks later, and, during this time the dogs undergo a soft diet.	PRF<br>10 mL venous blood redraw from a brachial vein for 2 sterile vacutainer tubes w/ no anticoagulant, and centrifugated at 2,700 rpm for 12 minutes.<br>The PRF clot was collected 2 mm below the lower dividing line.	Delayed replantation of mature extracted teeth w/ extraoral endodontic manipulation. Histologic evaluation.	The maxillary and mandibular arches were resected for histological evaluation.<br>There was a statistically significant difference between PRF and the control group regarding inflammatory RR, but not in connective tissue healing, replacement resorption (ankylosis), total resorption, and progressive RR.<br>Despite PRF demonstrating greater epithelial attachment and less inflammation, the comparison was not statistically significant.<br>The frequency of overall RR, extent, and depth of resorption, the healing process of resorption, and ankylosis were not statistically significant between the 2 groups.	Dogs were euthanized 8 weeks after treatment.

RCT- Randomized controlled Trial; W/-With; PRF-Platelet-rich-Fibrin; PRP-Platelet-rich-plasma; PT-Pulp tests; RR-Root resorption; GIC- Glass ionomer cement; REP-Regenerative endodontic procedure; PA-Periapical; ICM- Intracanal medication; CEJ- Cement enamel junction; RC- Root-canal; RPM revolutions per minute; BC- Blood clot; IP- Irreversible pulpitis; TAP-Tri-antibiotic paste; DAP-Double-antibiotic paste; HA-Hydroxyapatite; HPDLSC- Human periodontal ligament stem cells; PDLC- Periodontal ligament cells; PD- Probing depth; MTA- Mineral trioxide aggregate

Table S8. Autotransplantation studies

Author & year	Study type	Sample size	Sample characteristics	Pulp/PA diagnosis	Study protocol	Platelet concentrate used and protocol	Procedure	Treatment outcome	Follow-up
**Iqbal *et al.* 2021** [285]	Systematic Review	One included study about autotransplantation	N/A	Fragile fracture apicoectomy	Fragile fracture apicoectomy; PRP placed in the alveolar socket and root tip. 2 weeks post-treatment, orthodontic traction was applied.	PRP	Auto transplanted mature mandibular premolar	According to the systematic review, there is reasonable evidence that pulp regeneration can be achieved via tissue engineering w/ PRP and PRF in cases of transplantations	3 weeks and 3, 6, 12, 24 and 36 months
**Gaviño Orduña *et al.* 2020** [24]	Case Report	N=1 tooth	22-year-old female w/ hypodontia of teeth 14, 15, 24 and 25 w/ deciduous teeth 64 and 65. Treatment plan: extraction of 64 and 34, autotransplantation of tooth 44 to tooth 14 position.	Vital and healthy	The receptor site was prepared using dental implant drills w/ saline irrigation. The diameter of the site was slightly larger than the size of the donor tooth. Atraumatic extraction of tooth 44. A 3mm fragile fracture was made extra-orally w/ turbine to produce a circular grove in the root not invading the root canal. Then the fragile fracture root.end resection was performed using a dental elevator, maintaining the apical pulp tissue intact. PRP was prepared and placed in the receptor site	PRP Manufacturer´s instruction (PRGF-Endoret) - 36 mL venous blood was collected into 9 mL extraction tubes w/ 3.8% sodium citrate as an anticoagulant. Centrifugated at 580×g for 8 min at room temperature using a BTI System centrifuge (BTI Biotechnology Institute S.L).	Pulp revascularization of an Auto transplanted mature tooth w/ fragile fracture apicoectomy	The patient had no discomfort, symptoms of pain, or inflammation on follow-up visits.<br>At 36 months follow-up, the transplanted tooth was properly aligned w/ occlusal and interproximal contacts. Response to cold PT was positive. CBCT was taken to evaluate PA healing. There was no PA radiolucent lesion and no replacement or inflammatory RR or presence of pulp canal obliteration.	3 weeks and 3, 6, 12, 24 and 36 months

		first part w/out activation was injected into the apical third of the receptor alveolar bone, and the second part was activated w/ 300 μL of 10% calcium chloride to form a 3-D fibrin clot and used to fill the apical third of receptor alveolar bone. The donor tooth was placed in the site and fixed using a silk suture		

W/- With; PRF- Platelet-rich-Fibrin; PRP- Platelet-rich-plasma; PA- Periapical; N/A- Not applicable; PT-Pulp test; CBCT-Cone beam computerized tomography; PRGF-Plasma rich in grown factors

Table S19. Biological effects evaluation studies

Author & year	Study type	Sample size	Sample characteristics	Pulp/PA diagnosis	Study protocol	Platelet concentrate used and protocol	Procedure	Treatment outcome	Follow-up
**Moraschini *et al.*, 2023** [294]	Systematic Review	16 *in vitro* studies The number of samples ranged from 5 to 64 in each included study.	*In vitro* growth of bacteria and fungus and application w/ different types of PRF	N/A	The antimicrobial effects of PRF variations were compared to each other and w/ controls, such as phosphate-buffered saline, PRP, platelet-poor plasma, chlorhexidine, and antibiotics. One study did not use a control group. Over 16 subgroups of bacteria from the oral, periodontal, and endodontic environments were analyzed. The most used culture set was blood agar plates. The culture and response time ranged from 16 to 168 h.	PRF 3,000 and 2,700 rpm for 10-12 minutes	Antimicrobial effect evaluation of PRF	2 studies compared PRF to I-PRF. PRF had a positive effect, but I-PRF was better. The other 2 studies concluded that the PRF membrane was not able to inhibit *C.Albicans* after 24 h. One study concluded that H-PRF had a greater antimicrobial effect than PRF against *Staphylococcus aureus and Escherichia coli* after 24 h.	N/A
**Khatri *et al.*, 2023** [380]	*In vitro* study	15 single-rooted extracted teeth were sectioned at the CEJ level	Extracted teeth	N/A	The apical end of the sectioned teeth was trimmed to obtain a 10 mm length for all specimens. RC was prepared using burs to achieve a 4 mm diameter. Irrigation w/ 1.5% NaOCl,	.PRF 5 mL of blood was drawn and transferred to 5 mL tubes w/out anticoagulant and centrifuged at	Evaluation of Ca^{2+} ions release and pH from MTA w/ or w/out PRF	The pH and Ca^{2+} ion release were significantly lower in the experimental groups (PRF+MTA and BC+MTA). The pH increased in all experimental groups over time. Ca^{2+} ion release peaked on day 7 and reduced	N/A

		Group I: MTA+ PRF Group II: MTA+ BC Group III: MTA (control)			saline, and 17% EDTA was performed. The coronal end of the root was sealed w/ wax. MTA was condensed into the prepared canal to obtain a thickness of 4 mm. The BC and PRF were inserted. Teeth were maintained in 10 mL distilled water at 37ºC	3,000 rpm for 10 min		significantly on the 14th day for all groups. PRF and BC influenced the pH and Ca^{2+} ion release from MTA	
**Panda *et al.*, 2023** [376]	*In vitro* Study	N/A	Group I: MTA Group II: Tetric N-Bond universal bonding agent Group III: Theracal PT Group IV: PRF	HDPSCs were isolated from healthy extracted permanent third molar w/ a sharp spoon excavator.	PRF was cut into 1 × 1 mm pieces using a scalpel and directly applied to the cells. Cytotoxicity was accessed at 24, 48, and 72-hour intervals.	PRF 10 mL of venous blood was collected into 5 mL tubes w/out anticoagulant and centrifuged at 3,000 rpm for 15 minutes	Cytotoxicity evaluation	PRF group had the highest cell viability after 48 hours then MTA, Theracal PT, and Thetric N-bond. PRF presented the highest potential to enhance cell proliferation and differentiation followed by Theracal PT, MTA, and Thetric N-Bonding agent at the 24 and 72 hours. PRF managed to keep the viability of HDPSC more effectively than the other agents.	N/A
**Sandra *et al.*, 2023** [144]	Narrative Review	N/A	N/A	N/A	N/A	PRF & PRP	Review evaluating the potential of blood-derived bioscaffolds	Unlike PRP, PRF does not dissolve quickly after application, as it is formed slowly as natural BC. When combined w/ stem cells, PRP and PRF show better regeneration potential.	N/A

								hDPSCs co-cultured w/ 10% PRP show higher expression of fetal liver Kinase(Flk)-1, VEGF, PDGF, and SDF-1. PRP can promote vasculogenesis. The combination of PRP+ hDPSCs showed a significant increase in cell migration, proliferation, and differentiation, although, in the HDPSCs+ PRF group, the cell migration was faster.	
**Elver *et al.*, 2023** [114]	Narrative Review	N/A	N/A	N/A	N/A	PRF 2,700 rpm for 12 min (following the protocol by Chandra *et al.* 2019)	Evaluation of the biological effect of PRF in tissue healing	PRF accelerates the formation of new vessels and wound healing by affecting angiogenesis. Using PRF and PRP increases cell viability. PRF accelerates bone regeneration and decreases inflammatory RR.	N/A

W/-With; PRF-Platelet-rich-Fibrin; PRP-Platelet-rich-plasma; I-PRF- Injectable platelet rich fibrin; PA-Periapical; CEJ- Cement enamel junction; RC- Root-canal; RPM revolutions per minute; BC- Blood clot; HDPSC-Human dental pulp stem cells; MTA- Mineral trioxide aggregate; EDTA- Ethylenediaminetetraacetic acid. N/A- Not applicable; VEGF-Vasoendotelial growth factor; PDGF-Platelet-derived growth factor; SCDF-1-Stromal cell-derived factor 1

Table S10. Endo-Perio lesions studies

Author & year	Study type	Sample Size	Sample characteristics	Pulp/PA diagnosis	Study protocol	Platelet concentrate used and protocol	Procedure	Treatment outcome	Follow-up
**Choudhary *et al.*, 2023** [173]	RCT	N=280 patients<br><br>Group PRF:140<br>Group T-PRF:140	18–58-year-old patients w/ EPL in mandibular molars	1, 2, 3 walled infrabonny abnormalities associated with EPL in mandibular molars	All teeth underwent RC therapy to manage the endodontic part of the lesion, and then, the periodontal surgery where the infrabony lesions were filled w/ platelet scaffolds	PRF<br>10 mL of venous blood was collected in a 10 mL glass tube w/ no anticoagulant and centrifugated for 10 minutes at 3,000 rpm.	Management of EPL	PD was measured after 3 and 6 months. In both PRF and T-PRF groups, there was a clear reduction of PD in both follow-up visits, w/ a significant statistical difference from the control group; however, w/ no statistically significant difference between the 2 groups.<br>Attachment level was also measured at 3 and 6 months and the same happened as PD evaluation. There was an increase in attachment in both groups but w/ no statistically significant difference between them.	3 and 6 months
**Thakur *et al.*, 2023** [212]	RCT	N=40 patients<br>PRF medium group=20<br>PRF high group=20	16–60-year-old patients	Symptomatic apical periodontitis and PA defects w/ apicomarginal communication	All teeth underwent RC therapy to manage the endodontic part of the lesion, and then the surgical phase where a flap was made, the apicomarginal defect was identified, and curettage of the lesion was	PRF<br>10 mL of venous blood was collected in a 10 mL glass tube w/ no anticoagulant and centrifugated	Management of EPL.	Evaluation parameters: clinical assessment; radiographic assessment; quality of life; histologic assessment<br>The PRF medium group patients reported significantly less swelling on the 1st, 2nd,	12-months

				in the non-vital tooth.	performed. The tooth tip was 3mm sectioned w/ a bevel using turbine bur. Root end preparation was done and filled w/ MTA. PRF clots were placed into the bone defect and other PRF clot was squeezed in sterile gauze to form a membrane and was applied on the denuded root surface before flap repositioning w/ 5-0 sutures.	for 10 minutes at 2,700 rpm.		and 3rd days, and average pain on the 2nd, 3rd, and 4th days postoperatively. The difference in success rate for PA healing was non-significant between the PRF medium (89.5%) and PRF high (90%), in both 2D and 3D imaging evaluation. The formation of buccal bone was observed in five cases (26.3%) and four cases (20%) in the PRF medium and PRF high groups, respectively, without a significant difference. PRF medium clots had a loose fibrin structure w/ a significantly higher number of neutrophils (473.79 ± 82.89 per mm^2) than PRF high clots, which had a dense structure and fewer neutrophils (253.15 ± 63.86 per mm^2) ($p < 0.001$). Autologous platelet concentrates promoted satisfactory periapical healing, w/ no significant difference between the groups.	
**Oktawati *et al.,***	Systematic	16 case reports.	NR	PA periodontitis with	All studies included the management of the EPL using RC treatment alone,	One of the studies used PRF to manage the EPL	Management of EPL	All the studies showed a decreased PD after the healing of the lesion.	From 6 to 24 months

**2020** [299]	Review	N= 22 patients		associated EPL.	RC treatment combination w/ bone graft, or RC treatment w/ PRF. In one study, RC treatment was performed extra orally and then the tooth was reimplanted.				
**Ardila *et al.*, 2022** [408]	Scoping Review	1 non-randomized clinical trial: 140 teeth Group A: PRF Group B: T-PRF	Patients were 18-58 years old 60 females and 80 males	Primary endodontic lesion with secondary periodontal involvement	Conventional RC treatment was performed before periodontal surgery. PRF was laid in the infrabony defect during periodontal surgery	PRF	Management of EPL	Improvement in PD and an increase in CAL were also reported.	6 months
**Onicas *et al.*, 2021** [138]	Systematic Review	6 studies were included in the review describing 174 patients treated w/ PRF.	The sample in the included studies varies from 1 to 140 patients w/ age from 18 to 59 years old	PA periodontitis associated w/ EPL	In the included studies, treatment was divided into two phases. First, endodontic, and second, periodontal. In some studies, RES was performed as well. PRF was applied to the infrabonny defects during periodontic surgery	PRF Relative centrifugal force of 700×g to 200×g for 12 minutes.	Review of clinical trials, case reports or case studies on the management of EPL using PRF	In a randomized controlled trial conducted on 15 patients after RES, the PRF was applied to the periodontal defects and the success rate was 83.33%. In one study using L-PRF, PD was reduced from 11mm to 3 mm and from 14 mm to 5 mm. Encouraging results were found in 2 case reports in which, at 9 months follow-up, the PD had reduced from 10 to 4 mm and from 8 mm to 3 mm.	Up to 12 months

W/- With; PRF- Platelet-rich-Fibrin; PRP- Platelet-rich-plasma; PA- Periapical; RC- Root-canal; RPM- revolutions *per* minute; BC- Blood clot; RCT- Randomized controlled trial; PD- Probing depth, CAL- Clinical attachment level; EPL- Endo-perio lesions; RES- Root-end surgery; MTA- Mineral trioxide aggregate; N/A- Not applicable; NR- Not reported

Table S11. Root fracture studies

Author & year	Study type	Sample size	Sample characteristic	Pulp/PA diagnosis	Study protocol	Platelet concentrate used and protocol	Procedure	Treatment outcome	Follow-up
**Kapoor *et al.*, 2015** [44]	Case Report	N=1 tooth	18-year-old male w/ pain in upper left front teeth for last 3-4 months. PT showed no response. Tenderness to palpation, percussion, and grade II mobility	The radiograph showed a horizontal fracture of the middle third of the root w/ radiolucency near the fracture site suggesting the presence of granulation tissue. The apical segment was displaced from its anatomical position. Pulp canal obliteration and attempted RC treatment.	After local anesthesia, a full-thickness periosteal flap was done, and it was clear a bone defect in the buccal site due to the lesion. Granulated tissue was removed and the pulp space at the fracture area was filled with MTA. The displaced apical segment was removed. The bone cavity was filled w/ PRF	PRF	Managemen t of radicular fracture	After a week, soft tissue healing was satisfactory. At the follow-up, a reduction of radiolucency was evident, and the mobility and PD decreased to a normal level.	1 week, 6 months, 8 months and 18 months
**Pruthi *et al.*, 2020** [366]	Case series	N=1 tooth	16-year-old w/ pain in maxillary right central incisor due to trauma 2 days	Horizontal root fracture at the junction of the	First visit: local anesthesia. The tooth was repositioned and fixed w/ a splint for 3 weeks. Cold and electric pulp	PRF 8 mL of venous blood was collected in a 10	Managemen t of radicular fracture	The patient was asymptomatic at the 2-year follow-up visit and the tooth had no mobility and was	3, 6, 12 and 24 months

			early. Tenderness to percussion and class II mobility	middle and apical thirds.	testing were consistent w/ IP diagnostic.<br>Second visit: RC treatment of the coronal fragment was done, an access opening was created, working length was determined w/ an apex locator. Canal instrumentation was done with K-files and irrigated w/ 3% NaOCl. Calcium hydroxide was used for 1-week as ICM.<br>Third visit: ICM was removed. PRF was placed in the fracture site, Biodentine was condensed over PRF and the access cavity was sealed w/ nanohybrid composite resin.	mL glass tube w/ no anticoagulant and centrifugated for 10 minutes at 3,000 rpm. The PRF clot was squeezed in sterile gauze.		functional. A radiographic assessment showed that the fracture site had been repaired by interposition of connective tissue.	
**Arango-Gómez *et al.*, 2019** [1]	Case Report	N=1 tooth	19 year-old patient with multiple root fractures in teeth 21 and 11, 2 years prior	Previosly treeted teeth using calcium hydroxide paste (treatment failed) with chronic periapical abcess and horizontal root fractures	First visit: Local anesthesia. Working length determined, irrigation w/ 1,25% sodium hypochlorite w/out instrumentation TAP was applied and tooth sealed with glass ionomer.<br>Second visit:PRP was prepared, RC irrigated w/ NaOCl and 5ml of sterile saline. RC dried w/paper points and irrigated w/ 17% EDTA. 60 K-file to induce	PRP<br>Blood was drawn into sterile tubes with 3.8% sodium citrate that were centrifuged for 10 minutes at a speed of 3,000 rpm in a standard laboratory centrifuge (ROTOFIX 32; Andreas Hettich	Managemen t of radicular fracture	The tooth treated with PRP showed better calcification of the root fractures than the tooth treated with BC	3, 7, 12 and 36 months

			bleeding and then injection of PRP in tooth 11 only. 3-mm-thick MTA was applied and temporary glass ionomer filling.	GmbH & Co., Tuttlingen, Germany). The PRP obtained was then transferred to a 10 mL hypodermic syringe to be injected into the canal	

W/- With; PRF- Platelet-rich-Fibrin; PRP- Platelet-rich-plasma; RC- Root-canal; RPM- revolutions per minute; PD- Probing depth, ICM- Intracanal medication; IP-irreversible pulpitis; MTA- mineral trioxide aggregate; PA- Periapical; BC-Blood clot; EDTA- ethylenediaminetetraacetic acid; TAP-Triple antibiotic paste

Table S12. Root Perforation studies

Author & year	Study type	Sample Size	Sample characteristics	Pulp/PA diagnosis	Study protocol	Platelet concentrate used and protocol	Procedure	Treatment outcome	Follow-up
**Mohamed *et al.*, 2023** [324]	Animal study	N=90 dog teeth<br><br>Neg.Control : W/out furcal Pf. Pos.Control =Furcal Pf w/out r: pair Group 1: MTA Group 2: MTA+PRF Group 3: MTA+CGF These groups were subdivided into non-contaminated (n=10) and contaminated (n=10)	3 healthy premolar teeth were chosen from each quadrant of 12 mongrel dogs. The teeth had mature roots, intact crowns, and no periodontal defects	Healthy premolar teeth	Teeth Isolation and disinfection, access cavity, root length determined, instrumentation and irrigation w/ 2.5% NaOCl, and final rinse w/ 17% EDTA. Canals dried and obturated w/ single cone and AH plus sealer. Contaminated subgroup: Pf was done at pulp floor w/ a turbine bur. Pf was standardized to 1.5 × 2 mm. The access cavities were left without coronal sealing for 4 weeks Non-contaminated subgroup: the access cavity was sealed w/ sterile cotton and temporary filling w/out any Pf being done. After 4 weeks, the access cavity was opened, and the Pf was flushed w/ 2.5% NaOCl and 9% saline. The inflammatory tissue was	PRF 10 mL of blood were drawn from the dogs and inserted in 10 mL sterile tubes w/out anticoagulant and centrifugated at 3,000 rpm for 12 min	Management of furcation Pf in dogs' premolars	Methods of evaluation: radiographic; histologic (inflammatory cell count, new cementum deposition, new bone formation, epithelial proliferation, immunohistochemistry). The MTA+PRF and MTA+CGF groups demonstrated significantly more bone formation, OPN immunolocalization, and fewer inflammatory cell counts than the MTA group. The MTA, MTA+PRF, and MTA+CGF groups showed significantly more favorable radiographic, histological, and immunohistochemical healing features than the positive control, especially in non-contaminated subgroups, that showed significantly better features than the contaminated ones.	3 months

					removed, and bleeding was stopped. In the MTA group, Pf was treated with MTA application, 3mm thickness. In the PRF+MTA and CGF+MTA groups pieces of the scaffolds were packed into the furcations w/ hand pluggers to the CEJ level. 3 mm thickness MTA was placed on top. The access cavity was sealed w/ GIC.				
**Teja *et al.*, 2021** [84]	Case Report	N=1 tooth	38-year-old female w/ pain in left back tooth region for 10 days. Tender to vertical percussion. The mandibular first molar had been submitted to multiple attempts of RC treatment. The radiograph showed radiolucency involving the RC space, break in the lamina dura continuity at the mesial furcation w/ radiolucency	Pulpal necrosis w/ symptomatic apical periodontitis	Anesthesia and isolation. The pulp chamber was disinfected using 2 ml of 2% CHX and saline. The pulpal floor examination showed a furcation Pf; when accessed w/ a #10k-file, the mesiolingual canal started bleeding, suggestive of a stripped perforation. Working length was determined and Pf was radiographically confirmed; the canals were rinsed w/ saline and dried. The canals were prepared using rotary ProTaper Gold files 1mm short from working length and disinfected w/ 10 mL of	PRF	Management of strip Pf in furcation and mesial canals of mandibular first molar	After 3 months, a prosthetic crown was made. The patient did not suffer any associated complications for a period of 1 year	3 months, 6 months, and 1 year

			surrounding the furcal area and mesial root apex		1% NaOCl. Calcium hydroxide was used as ICM for the visits. In the 4 visits: the patient was asymptomatic, irrigation w/ 1% NaOCl and 17% EDTA was done and canals dried. PRF obtained was cut into pieces and condensed in the Pf site, MTA was packed, and the canal was plugged w/ MTA. The other canals were obturated using gutta-percha and a bioactive sealer. After 2 days, a permanent restoration was performed w/ composite resin.				
**Cordova-Malca *et al.*, 2022** [15]	Case Report	N=1	21-year-old female	Failed RC therapy w/ symptomatic apical periodontitis	Anesthesia, isolation, and access cavity were performed, and the Pf site was identified (2-3 mm in diameter). Removal of obturation material was done. The working length was determined, and irrigation was performed with 2.5% NaOCl and final irrigation with 17% EDTA and distilled water. The RC was dried and obturated w/ gutta percha cones using a	PRF 18 mL of blood were drawn and inserted in 6 mL sterile tubes w/out anticoagulant and centrifugated at 3,000 rpm for 10 minutes (800D, Hiprove, Jiangsu, China)	Management of strip Pf in the canal and radicular cyst	After the first surgery, the patient returned 1 year later w/ a sinus tract due to failure in the Pf management. After the second microsurgery, 1-week and 2-week recalls showed a favorable evolution, and a crown was placed on the tooth. After 2 years cyst had healed, and a newly formed bone was present in the perforation area.	1.2 weeks and 2-year follow-up

					continuous wave technique. The access cavity was sealed w/ GIC. Surgical phase: a full-thickness flap was performed to access the Pf site. The lesion was removed, and apicectomy was performed by removing 3 mm of apex. Retrograde filling w/ Bioceramic material; bone defect filled w/ PRF and 1g of Bio-oss. Second surgical phase: after 1 year, the patient appeared w/ a sinus tract. Although the radicular cyst had decreased, there was a clinical failure in the Pf area. The same protocol was used for another micro-surgery. MTA was placed in the Pf followed by 0.25g Bio-oss and PRF membrane				
**Elver *et al.*, 2023** [114]	Narrative review	N/A	N/A	N/A	N/A	PRF 2.700 rpm for 12 min (following the protocol by Chandra *et al.* 2019)	Management of pulp floor Pf.	The review points to the utility of PRF in pulp floor Pf management	N/A

**Pruthi *et al.,* 2020** [366]	Case series	N=1	33-year-old woman w/ pain in mandibular right first molar, w/ incomplete RC treatment 6 months before.	Chronic apical periodontitis. Tooth severely damaged w/ pulp chamber opened and tenderness to percussion. Iatrogenic furcation Pf w/ bone loss in the furcal area and around the apex.	Isolation, access cavity made using a microscope, working length determined. RC was debrided w/ protaper universal instruments and irrigated w/ 3% NaOCl. The Pf area was irrigated w/ 1% NaOCl. Calcium hydroxide was placed in the canals and Pf, and the access cavity was restored.<br>1 week later, the ICM was removed, the orifices of the canals were blocked w/ cotton pellets and PRF was placed in the Pf, and Biodentine was compacted over PRF w/ pluggers. The canals were then obturated w/ gutta percha and AH Plus sealer.<br>The tooth was restored w/ a nanohybrid composite resin.	PRF<br>8 mL of venous blood was collected in a 10 mL glass tube w/ no anticoagulant and centrifugated for 10 minutes at 3,000 rpm. The PRF clot was collected and squeezed in sterile gauze to remove excess fluid.	Management of furcal Pf	2 weeks after treatment, the patient had no symptoms, and the tooth was restored w/ crown.<br>At 3, 6,12, and 24 months, the tooth was asymptomatic. w/ no mobility and functional.<br>At 24 months a radiograph showed a complete healing of furcal and PA lesions.	3, 6, 12 and 24-months

W/- With; PRF- Platelet-rich-Fibrin; PRP- Platelet-rich-plasma; CGF- Concentrated growth factors; RC- Root-canal; RPM- revolutions per minute; ICM- Intracanal medication; IP-Irreversible pulpitis; MTA-mineral trioxide aggregate; PA- Periapical; CEJ- Cemento enamel junction; Pf- Perforation; CHX-Chlorhexidine; GIC- Glass ionomer cement; EDTA- Ethylenediaminetetraacetic acid; N/A- Not applicable; OPN-Osteopontin

Table S13. Mechanical Properties evaluation studies

Author & year	Study type	Sample size	Sample characteristics	Pulp/PA diagnosis	Study protocol	Platelet concentrate used and protocol	Procedure	Treatment outcome	Follow-up
**Chhaya *et al.*, 2022** [387]	*In vitro* study	18 membranes	PRF membranes	N/A	The modulus of elasticity and hardness, and the degradation rate of PRF membrane, bovine collagen membrane, fish collagen membrane, and chorionic membrane were assessed. The membranes were also evaluated under a microscope.	PRF<br>10 mL of venous blood was drawn w/out any anticoagulants and centrifuged at 3,000 rpm for 10 minutes.<br>PRF membrane was done by compressing the PRF gel.	To compare the mechanical properties of different PRF membranes	Bovine collagen had the highest strength and PRF membrane the lowest. The degradation rate after 1 week was highest in the PRF membrane. Microscopic analysis shows bovine collagen had the highest number of collagen fibers. Only PRF had cellular distribution in its membrane, while the other commercial membranes had a significantly higher number of collagen fibers and the absence of any cellular component. The bovine membrane had the best mechanical properties.	N/A
**Nagaraja *et al.*, 2019** [388]	*In vitro* study	10 samples	PRF and PRF matrix	N/A	PRF and PRF matrix were subjected to analysis of platelet and leucocyte concentration. The pH of PRF and PRF matrix were evaluated over 5 days using a pH meter. PRF and PRF	PRF<br>4 mL venous blood was collected and centrifuged at 400×g for 10 minutes in a Remi Elektrotechnik	Evaluation of components and morphology of PRF and PRP matrix	There was a decrease in the pH in PRF which indicates an increasing acidity over 5 days. On microscopic observation, PRF showed a less dense fibrin network w/ thinner fibrous strand.	N/A

		matrix were subjected to histological and scanning electron microscopy.	Limited Vasai, Bharat

W/-With; PRF-Platelet-rich-Fibrin; PRP-Platelet-rich-plasma; RPM revolutions per minute; PA-Periapical; N/A- Not applicable

Table S14. Root Resorption studies

Author & year	Study type	Sample size	Sample characteristics	Pulp/PA diagnosis	Study protocol	Platelet concentrate used and protocol	Procedure	Treatment outcome	Follow-up
**Dadpe *et al.*, 2023** [282]	Systematic Review	N=1 tooth	One case report of avulsed tooth w/ mature apex	Mature tooth with pulp necrosis and avulsion	Pulp extirpation, irrigation with normal saline and 5.25% NaOCl, root apex was enlarged to 1.5-2 mm, soaked in doxycycline solution for 15 to 20 minutes the tooth was then reimplanted and PRP was injected inside the canal, and the canal was sealed with GIC.	PRP	Management of different types of RR	Positive response to PT	12 months
**Gupta *et al.*, 2022** [31]	Case Report	N=1 tooth	Young girl with decayed and discolored tooth. Trauma history when 10 years old. Swelling of the labial sulcus in relation to #21. The radiograph showed a large PA area along with an internal RR.	Pulp necrosis and internal inflammatory RR w/ associated PA lesion	First visit: anesthesia, isolation, and access cavity and decay removal in #21. Irrigation w/ 2.5% NaOCl and saline. Working length was determined and cleaning and shaping of the canal was performed. Calcium hydroxide was used as ICM for 2 weeks. Second visit: ICM was removed w/ 5% NaOCl and canal the was dried. TAP (ciprofloxacin;	PRF 10 mL of venous blood was collected into a sterile tube w/out anticoagulant and centrifuged at 3,000 rpm for 10 minutes.	Apexification in teeth with Internal RR	At 3 months the patient was asymptomatic and partial resolution of radiolucency was observed, so a ceramic crown was placed. At 6 months, a complete resolution of radiolucency was seen. After 24 months there was no abnormal finding.	3, 6, and 24 months

					metronidazole; doxycycline) was inserted in the canal for 2 weeks. After 2 weeks, TAP was removed, and a calcium hydroxide dressing was placed until the canal orifice to reduce the potential tooth discoloration. After 2 weeks PRF was mixed w/ HA and inserted in the canal 1-2 mm apical of the canal. After, MTA was placed in small increments until a barrier of 4.5 mm was established. MTA was also placed in the canal walls				
**Kapoor *et al.*, 2015** [44]	Case report	N=1	18-year-old male w/ pain in upper left front teeth, that lasted from 3-4 months. Vitality tests showed no response. Tenderness to palpation, percussion, and grade II mobility.	Pulp canal obliteration and attempted RC treatment. RR of the coronal segment. The radiograph showed a horizontal fracture of the middle third of the root w/ radiolucency near the fracture.	After anesthesia, a full-thickness periosteal flap was done, and it was clear a bone defect in the buccal site due to the lesion. Granulated tissue was removed and the pulp space at the fracture area was filled w/ MTA. The displaced apical segment was removed. The bone cavity was filled w/ PRF and a suture was done.	PRF	Management of RR involving the coronal root segment.	After a week, soft tissue healing was satisfactory. At the follow-up, it was evident a reduction in radiolucency, mobility, and PD decreased to a normal level. Follow-up showed the arrest of the resorption process.	1 week, 6 months, 8 months, and 18 months

**Johns *et al.*, 2013** [40]	Case report	N=1	35-year-old man, w/ swelling and pus discharge in the upper left canine. History of trauma about 20 years before.	Localized chronic periodontitis and pulp necrosis with external inflammatory RR of #11. The radiography showed a radiolucency at the mesial side of the root.	RC treatment was done. The access cavity was prepared after isolation and the working length was determined w/apex locater and RC was shaped and cleaned w/ rotary Ni-Ti Protaper files using crown down technique. Irrigation w/ 2.5% NaOCl. After a week, the tooth was asymptomatic. RC was irrigated and obturation was done w/ a selected gutta-percha master cone and AH-Plus sealer. Access cavity restored w/ composite. Surgical procedure on vestibular mucosa: full-thickness mucoperiosteal flap was elevated. Inflammatory tissue was removed and 90% of trichloroacetic acid was applied to the resorptive defect for coagulation necrosis. The resorptive defects were restored w/ microfilled composite and resin-modified GIC. PRF was placed over the root w/	PRF 12 mL of blood was drawn from the patient's right antecubital vein and centrifuged at 3,000 rpm for 10 min in a REMI Model R-8c with 12 mL × 15 mL swing out head. The PRF clot was squeezed into a membrane.	REP in teeth with External RR	The patient was asymptomatic 1 week after the surgery. At 2 years, periodontal status demonstrated mild mobility, normal PD, no recessions, and no loss of clinical attachment.	1 week, 6 months, 12 months and 24 months

					HA and the flap was closed w/ 4-0 suture.				
**Nalawade *et al.*, 2011** [60]	Case report	N=2 teeth	11-year-old girl w/ intrusive luxation and extrusive luxation of teeth 11 and 12	Traumatic Luxation of the central incisors w/ inflammatory RR	Tooth were fixated w/ a splint and RC treatment was performed in both teeth. Under generalized anesthesia, the mucoperiostic flap was elevated and root resorption areas were carefully curettage. The apicectomy of 11 and 12 was done and sealed with GIC. The surgical cavity was filled w/ PRP and bone graft and closed	PRP + bone graft 8 ml of venous blood was collected and centrifuged. Before application, PRP was mixed w/ bioactive synthetic bone draft, thrombin, and calcium chloride.	Management of inflammatory RR	After 18 months, the patient had no symptoms and there was no mobility of the teeth. There were radiographic signs of bone graft resorption.	18 months

W/- With; PRF- Platelet-rich-Fibrin; PRP- Platelet-rich-plasma; RC- Root-canal; RPM revolutions per minute; ICM- Intracanal medication; MTA- mineral trioxide aggregate; PA- Periapical; GIC- Glass ionomer cement; EDTA- Ethylenediaminetetraacetic acid; TAP- Tri-antibiotic paste; DAP- Dual antibiotic paste; PD- Probing depth; PDL- Periodontal ligament; PT- Pulp test; BC-Blood clot; REP- Regenerative endodontic procedure; HA- Hydroxyapatite; RR- Root resorption.

Table S15. Regenerative endodontic procedures on mature teeth studies

Author & year	Study type	Sample size	Sample characteristics	Pulp/PA diagnosis	Study protocol	Platelet concentrate used and protocol	Procedure	Treatment outcome	Follow-up
**Eldessoky *et al.*, 2023** [319]	Animal Study	N=40 Teeth<br>Group DAP= 10 teeth/20 roots<br>Group DL980nm= 10 teeth/20roots<br>Positive control= 10 teeth/20roots<br>Negative control= 10 teeth/20roots<br>The groups were subdivided into Group A- 1 month after treatment	2-year-old mongrel dogs	Mature teeth with pulp necrosis and apical periodontitis	To induce PA lesions: cavity access was carried out, exposing the pulp. Pulp was disrupted w/ a #15 k-file and a cotton pellet w/ dog´s supragingival plaque was collected and kept in a saline sterile solution so that a sponge could be dipped in it and put inside the pulp chamber. The coronal cavities were filled w/ cotton for a month after a piece of cotton was inserted in the entrance of each canal.<br>RC preparation: isolation was made, an apex locator was used for work length determination, and instrumentation was done w/ the Protaper universal system to the required length up to #F4. Irrigation was done with 2 mL of 1.5% NaOCl. Final rinse w/ 0.9% saline. The DAP group	PRF<br>20 mL of blood was collected from each dog's cephalic vein and centrifugated at 3,000 rpm for 10 min in a sterile tube w/out anticoagulant in a centrifuge (REMI Laboratories, Mumbai, India). PRF clot was squeezed w/ dry gauze and a finger plugger and cut into pieces	Revascularization of mature necrotic teeth	The dogs were euthanized, their jaws were separated, and bone segments, including the experimental and control teeth, were removed.<br>Findings in inflammation:<br>In subgroup A the highest number of inflammatory cells was recorded in the positive control followed by group DAP and DL. In subgroup B, the highest number of inflammatory cells was recorded in the positive group, followed by DAP, DL, and the control group.<br>In DL and DAP groups, the number of inflammatory cells decreased significantly after 3 months.<br>Nature and extent of vital tissue in-growth in the pulp space:<br>The newly formed tissue inside the canal resembled	Dogs were euthanized 1 month and 3 months after the treatment.

		and Group B- 3 months after treatment			received the DAP mixture in the canals and the access cavity was closed w/ GIC. In the DL980nm group, the laser fiber was applied 1 mm before the apex. After 1 month, the canals were reentered and in the DAP group, DAP was removed w/ irrigation of distilled water and 6 mL of 1.5% NaOCl and 17% EDTA solution. In the DL group, the canal was irrigated w/ 1.5% NaOCl solution and then activated w/ DL and 17% EDTA followed by 2 mL of 1.5% NaOCl irrigation. Then, in both experimental groups: cause bleeding w/ a #20K-file 2 mm outside the apex. BC formed over time. PRF membrane was inserted in the canal until the CEJ. MTA was used to plug orifices and GIC was used to close the coronal cavities.			periodontal connective tissue, w/ varying degrees of inflammatory cells and a low to moderate number of blood vessels that were engorged by red blood cells. New hard tissue formation: No statistically significant differences between the groups and subgroups in hard tissue formation. In subgroup B, new tissue revealed cementum-like tissue that lay down on the root´s outer surface. Bone resorption: No signs of bone resorption were recorded	
**Wu *et al.*, 2023** [217]	RCT	20 patients Group control=10 Group PRF=10	18–30-year-old patients w/ mature permanent teeth	Mature teeth w/ decayed pulp	First visit: the pulp was exposed w/ isolation; irrigation with 1.25% NaOCl for 5-10 min; rinse w/ saline for 5 min, the canal was	PRF	Revascularization of mature necrotic teeth	After treatment, the rate of clinical success of the PRF group was 100%, which was higher than the control group (50%). The thickness of the	21 months

					dried. TAP was prepared and applied into the canal, followed by a cotton pellet, and the canal was sealed w/ GIC. After 1 week, TAP was removed and, if the patient was asymptomatic on the second visit, the RC was irrigated w/ 20 mL of EDTA and dried. Intracanal bleeding was provoked w/ a #40k root file beyond the apex, and, in the PRF group, PRF was compacted into the canal. After, iRoot BP Plus was placed 4 mm below the enamel bone boundary. A wet cotton ball was placed above the iRoot and the cavity was sealed with GIC. On the day after, restoration w/ composite resin was made.<br>The control group followed the same protocol, except it didn't t receive PRF.			RC walls of the PRF group was also statistically significantly higher than the control group. However, the length of the crown/root was lower than the control group. Regarding dental function and satisfaction, the PRF group had a higher score of function and satisfaction compared to the control group, w/ a statistically significant difference.	
**Ahmed *et al.*, 2023** [165]	RCT	28 patients	Males or females aged between 9 and 30 years old with no systemic disease or under	Necrotic mature mandibular molars with chronic PA periodontitis	In the first visit, the teeth were instrumented, DAP was prepared and injected into the canals, and it was sealed with GIC. Three weeks later, the patients	PRP<br>Venous blood was collected from patients' forearm veins and transferred into a	Revascularization of mature necrotic teeth	Regarding the post-revascularization/obturation, there was no statistically significant difference between the pain in the two groups at all time intervals, except at 12	Post-operative pain was registered at 6, 12h, and daily for five days.

			systemic corticoid therapy		were randomized either to the control group where standard RC treatment was completed by lateral condensation technique w/ gutta-percha, or to the intervention group of PRP revascularization technique. DAP was removed by irrigation w/ NaOCl and saline and irrigation w/ 17%EDTA. After the dryness of the canals, hemorrhage was induced w/ a K-file 2mm over the apex, and when the bleeding reached the CEJ, the PRP was injected into the canal. CollaCote membrane was placed followed by a layer of MTA and a moister cotton pellet followed by GIC. 1 week later, the GIC was replaced with composite resin.	sterile sodium citrate vacuum tube. PRP was prepared by single centrifugation technique at 460×g for 8 minutes. PRP was collected using an automatic pipette and transferred to a sterile plain vacuum chloride to be used as liquid and injected inside the RC.		h, where the revascularization group showed a statistically significant higher prevalence of no pain than the RC treatment group. Regarding the healing, the PA lesions decreased significantly in size from the pre-operative values in both groups w/out a statistically significant difference between groups.	Clinical and radiographic healing were assessed after 6 and 12 months
**Li *et al.*, 2023** [288]	Systematic Review with Meta-analysis	27 RCTs included. N=178 mature permanent teeth and 676	The etiology of pulp necrosis was trauma, caries, and development abnormalities	Necrotic mature permanent teeth	The protocol varied in the 27 included RCTs. Irrigation with 1-5.25% NaOCl and saline was performed. The ICM used was mainly TAP or DAP but calcium hydroxide was also	PRF	REP in mature teeth	Outcome indicators were asymptomatic teeth, pulp sensitivity, and discoloration. The success rate for mature teeth was 95%. Mature teeth manage to have a high incidence of positive	From 12 to 28.25 months

		immature permanent teeth. Mature teeth treaded with PRF: 50			used in 4 studies. Final irrigation was 5-17% EDTA, except in 7 studies. 8 studies used PRF, 5 used PRP, and 4 used PRF and PRP in the experimental groups. However, only 3 RCTs reported the use of PRF in mature teeth			sensitivity response to electric PT, meaning the restoration of pulp sensibility was more evident in mature permanent teeth.	
**Elver *et al.*, 2023** [114]	Narrative Review	N/A	N/A	N/A	N/A	PRF 2,700 rpm for 12 min (following the protocol by Chandra *et al.* 2019)	REP in mature teeth	According to this review PRF can be an asset, and has proven to increase the healing and better prognosis of the REP approach of mature teeth (Youssef *et al.*, 2022)	N/A

W/- With; PRF- Platelet-rich-Fibrin; PRP- Platelet-rich-plasma; RC- Root-canal; RPM- revolutions per minute; ICM- Intracanal medication; MTA- mineral trioxide aggregate; PA- Periapical; GIC- Glass ionomer cement; EDTA- Ethylenediaminetetraacetic acid; TAP- Tri- antibiotic paste; DAP- Dual antibiotic paste; PT- Pulp test; BC-Blood clot; REP-Regenerative endodontic procedure; DL- diode laser; RCT- Randomized controlled trial; CEJ- Cement-enamel junction; N/A- Not applicabe

Table S16. PRISMA-ScR Check-list

SECTION	ITEM	PRISMA-ScR CHECKLIST ITEM	REPORTED ON PAGE #
**TITLE**			
Title	1	Identify the report as a scoping review.	1
**ABSTRACT**			
Structured summary	2	Provide a structured summary that includes (as applicable): background, objectives, eligibility criteria, sources of evidence, charting methods, results, and conclusions that relate to the review questions and objectives.	1
**INTRODUCTION**			
Rationale	3	Describe the rationale for the review in the context of what is already known. Explain why the review questions/objectives lend themselves to a scoping review approach.	2-3
Objectives	4	Provide an explicit statement of the questions and objectives being addressed with reference to their key elements (e.g., population or participants, concepts, and context) or other relevant key elements used to conceptualize the review questions and/or objectives.	2-3
**METHODS**			
Protocol and registration	5	Indicate whether a review protocol exists; state if and where it can be accessed (e.g., a Web address); and if available, provide registration information, including the registration number.	15
Eligibility criteria	6	Specify characteristics of the sources of evidence used as eligibility criteria (e.g., years considered, language, and publication status), and provide a rationale.	16
Information sources*	7	Describe all information sources in the search (e.g., databases with dates of coverage and contact with authors to identify additional sources), as well as the date the most recent search was executed.	15
Search	8	Present the full electronic search strategy for at least 1 database, including any limits used, such that it could be repeated.	Suplementary material
Selection of sources of evidence†	9	State the process for selecting sources of evidence (i.e., screening and eligibility) included in the scoping review.	16
Data charting process‡	10	Describe the methods of charting data from the included sources of evidence (e.g., calibrated forms or forms that have been tested by the team before their use, and whether data charting was done independently or in duplicate) and any processes for obtaining and confirming data from investigators.	16
Data items	11	List and define all variables for which data were sought and any assumptions and simplifications made.	16
Critical appraisal of individual sources of evidence§	12	If done, provide a rationale for conducting a critical appraisal of included sources of evidence; describe the methods used and how this information was used in any data synthesis (if appropriate).	-
Synthesis of results	13	Describe the methods of handling and summarizing the data that were charted.	5
**RESULTS**			
Selection of sources of evidence	14	Give numbers of sources of evidence screened, assessed for eligibility, and included in the review, with reasons for exclusions at each stage, ideally using a flow diagram.	3
Characteristics of sources of evidence	15	For each source of evidence, present characteristics for which data were charted and provide the citations.	5-7

SECTION	ITEM	PRISMA-ScR CHECKLIST ITEM	REPORTED ON PAGE #
Critical appraisal within sources of evidence	16	If done, present data on critical appraisal of included sources of evidence (see item 12).	-
Results of individual sources of evidence	17	For each included source of evidence, present the relevant data that were charted that relate to the review questions and objectives.	7-13
Synthesis of results	18	Summarize and/or present the charting results as they relate to the review questions and objectives.	7-13
**DISCUSSION**			
Summary of evidence	19	Summarize the main results (including an overview of concepts, themes, and types of evidence available), link to the review questions and objectives, and consider the relevance to key groups.	13-14
Limitations	20	Discuss the limitations of the scoping review process.	15
Conclusions	21	Provide a general interpretation of the results with respect to the review questions and objectives, as well as potential implications and/or next steps.	15
**FUNDING**			
Funding	22	Describe sources of funding for the included sources of evidence, as well as sources of funding for the scoping review. Describe the role of the funders of the scoping review.	17

Table S17. Databases search strategy

Database	Search Key	Filters
**Medline (using PubMed)**	("Platelet-rich fibrin"[Mesh] OR "Platelet-rich fibrin" OR “Fibrin, Platelet-Rich” OR "Fibrin Platelet Rich" OR "Platelet Rich Fibrin" OR "L-PRF" OR "Platelet-Rich Plasma"[Mesh] OR "Platelet-Rich Plasma" OR “Plasma, Platelet-Rich” OR “Platelet Rich Plasma” OR “Platelet-Rich Plasma” OR “thrombocyte rich plasma” OR PRF OR P-PRF OR PRP OR leukocyte-PRF OR thrombocyte-rich fibrin) AND ("endodontics"[Mesh] OR endodont* OR Apicoectom* OR “root end resection” OR “root end surgery” OR “root resection” OR “pulp capping*” OR “pulp cap” OR “Capping, Pulp” OR “Cappings, Pulp” OR “Capping, Dental Pulp” OR “Cappings, Dental Pulp” OR Pulpectom* OR Pulpotom* OR “Canal Therapies, Root” OR “Canal Therapy, Root” OR “Root Canal Therap*” OR “Therapies, Root Canal” OR “Therapy, Root Canal” OR “pulp canal therapy” OR “root canal procedure” OR Apexification* OR “apical closure” OR Apexogenesis OR Apexogeneses OR “Replantation, Tooth” OR “Replantations, Tooth” OR “Tooth Replantation*” OR “Reimplantation, Tooth” OR “Reimplantations, Tooth” OR “Tooth Reimplantation*” OR “tooth reinclusion” OR “dental reimplantation” OR “dental reinclusion” OR “dental replantation”) NOT ("Dental Implants"[Mesh] OR implant*)	Language: Portuguese, English, French, Spanish.
**Web of Science (all databases)**	("Platelet-rich fibrin" OR “Fibrin, Platelet-Rich” OR "Fibrin Platelet-Rich" OR "Platelet Rich Fibrin" OR "L-PRF" OR "Platelet-Rich Plasma" OR “Plasma, Platelet-Rich” OR “Platelet Rich Plasma” OR “Platelet-Rich Plasma” OR “thrombocyte rich plasma” OR PRF OR P-PRF OR PRP OR leukocyte-PRF OR thrombocyte-rich fibrin) AND (endodont* OR Apicoectom* OR “root end resection” OR “root end surgery” OR “root resection” OR “pulp capping*” OR “pulp cap” OR “Capping, Pulp” OR “Cappings, Pulp” OR “Capping, Dental Pulp” OR “Cappings, Dental Pulp” OR Pulpectom* OR Pulpotom* OR “Canal Therapies, Root” OR “Canal Therapy, Root” OR “Root Canal Therap*” OR “Therapies, Root Canal” OR “Therapy, Root Canal” OR “pulp canal therapy” OR “root canal procedure” OR Apexification* OR “apical closure” OR Apexogenesis OR Apexogeneses OR “Replantation, Tooth” OR “Replantations, Tooth” OR “Tooth Replantation*” OR “Reimplantation, Tooth” OR “Reimplantations, Tooth” OR “Tooth Reimplantation*” OR “tooth reinclusion” OR “dental reimplantation” OR “dental reinclusion” OR “dental replantation”) NOT (implant*)	Language: Portuguese, English, Spanish, Unspecified.<br><br>NOT Document types: Meeting or Book or Dissertation Thesis or Abstract or Editorial Material or News
**Embase**	('platelet-rich fibrin'/exp OR 'l-prf' OR 'p-prf' OR 'leukocyte-prf' OR 'platelet-rich fibrin' OR 'pure prf' OR 'thrombocyte-rich fibrin' OR 'fibrin, platelet-rich' OR 'fibrin platelet-rich' OR 'platelet rich fibrin' OR 'thrombocyte rich plasma'/exp OR 'platelet rich plasma' OR 'platelet-rich plasma' OR 'thrombocyte rich plasma' OR prf OR prp) AND ('endodontics'/exp OR endodontic* OR 'apicoectomy'/exp OR	Language: Portuguese, English, French, Spanish.

	'apicoectomy' OR 'root end resection' OR 'root end surgery' OR 'root resection' OR apicoectom* OR 'dental pulp capping'/exp OR 'dental pulp capping' OR 'pulp cap procedure' OR 'pulp cap technique' OR 'pulp capping' OR 'capping, pulp' OR 'cappings, pulp' OR 'capping, dental pulp' OR 'cappings, dental pulp' OR 'pulpectomy'/exp OR pulpectom* OR 'pulpotomy'/exp OR pulpotom* OR 'regenerative endodontics'/exp OR 'endodontic procedure'/exp OR 'pulp canal therapy' OR 'root canal procedure' OR 'canal therapies, root' OR 'canal therapy, root' OR 'root canal therap*' OR 'therapies, root canal' OR 'therapy, root canal' OR 'apexification'/exp OR 'apical closure (dental)' OR apexification* OR apexogenesis OR apexogeneses OR 'tooth replantation'/exp OR 'dental reimplantation' OR 'dental reinclusion' OR 'dental replantation' OR 'tooth reinclusion' OR 'replantation, tooth' OR 'replantations, tooth' OR 'tooth replantation*' OR 'reimplantation, tooth' OR 'reimplantations, tooth' OR 'tooth reimplantation*') NOT ('tooth implant'/exp OR implant*) AND ([english]/lim OR [french]/lim OR [portuguese]/lim OR [spanish]/lim) AND ([article]/lim OR [article in press]/lim OR [data papers]/lim OR [letter]/lim OR [review]/lim OR [short survey]/lim)	Type of article inclusion filter: article, article in press, data papers letter review and short survey.
**Cochrane library**	#1 MeSH descriptor: [Platelet-Rich Fibrin] explode all trees<br>#2 "Platelet-rich fibrin"<br>#3 "Fibrin, Platelet-Rich"<br>#4 "Fibrin Platelet Rich"<br>#5 "Platelet Rich Fibrin"<br>#6 L-PRF<br>#7 MeSH descriptor: [Platelet-Rich Plasma] explode all trees<br>#8 "Platelet-Rich Plasma"<br>#9 "Plasma, Platelet-Rich"<br>#10 "Platelet Rich Plasma"<br>#11 "Platelet-Rich Plasma"<br>#12 "thrombocyte rich plasma"<br>#13 PRF<br>#14 P-PRF<br>#15 PRP<br>#16 leukocyte-PRF<br>#17 "thrombocyte-rich fibrin"<br>#18 MeSH descriptor: [Endodontics] explode all trees<br>#19 endodont*<br>#20 MeSH descriptor: [Apicoectomy] explode all trees<br>#21 apicoectom*<br>#22 "root end resection"<br>#23 "root end surgery"<br>#24 "root resection"<br>#25 MeSH descriptor: [Dental Pulp Capping] explode all trees<br>#26 (pulp NEXT capping*)<br>#27 "pulp cap"<br>#28 (Capping* NEXT Pulp)<br>#29 (Capping* NEXT Dental Pulp)<br>#30 MeSH descriptor: [Pulpectomy] explode all trees<br>#31 Pulpectom*	

#32 Pulpotom*
#33 MeSH descriptor: [Root Canal Therapy] explode all trees
#34 (Canal Therap* NEXT Root)
#35 (Root Canal Therap*)
#36 (Therap* NEXT Root Canal)
#37 "pulp canal therapy"
#38 "root canal procedure"
#39 MeSH descriptor: [Apexification] explode all trees
#40 Apexification*
#41 "apical closure"
#42 Apexogenesis
#43 Apexogeneses
#44 MeSH descriptor: [Tooth Replantation] explode all trees
#45 (Replantation* NEXT Tooth)
#46 (Tooth Replantation*)
#47 (Reimplantation NEXT Tooth)
#48 (Tooth Reimplantation*)
#49 "tooth reinclusion"
#50 "dental reimplantation"
#51 "dental reinclusion"
#52 "dental replantation"
#53 MeSH descriptor: [Dental Implants] explode all trees
#54 implant*
#55 (#1 OR #2 OR #3 OR #4 OR #5 OR #6 OR #7 OR #8 OR #9 OR #10 OR #11 OR #12 OR #13 OR #14 OR #15 OR #16 OR #17) AND (#18 OR #19 OR #20 OR #21 OR #22 OR #23 OR #24 OR #25 OR #26 OR #27 OR #28 OR #29 OR #30 OR #31 OR #32 OR #33 OR #34 OR #35 OR #36 OR #37 OR #38 OR #39 OR #40 OR #41 OR #42 OR #43 OR #44 OR #45 OR #46 OR #47 OR #48 OR #49 OR #50 OR #51 OR #52) NOT(#53 OR #54)
166 results (21/12/2023)

References

1. Arango-Gómez, E.; Nino-Barrera, J.L.; Nino, G.; Jordan, F.; Sossa-Rojas, H. Pulp Revascularization with and without Platelet-Rich Plasma in Two Anterior Teeth with Horizontal Radicular Fractures: A Case Report. *Restor Dent Endod* **2019**, *44*, e35, doi:10.5395/rde.2019.44.e35.
2. Nagaveni, N.; Poornima, P.; Khan, M.M.; Mathew, M.G.; Soni, A.J. A Comparative Evaluation of Revascularization Done in Traumatized Immature, Necrotic Anterior Teeth with and without Platelet-Rich Fibrin: A Case Report. *Int J Clin Pediatr Dent* **2020**, *13*, 98–102, doi:10.5005/jp-journals-10005-1738.
3. Pathak, D.; Bansode, D.; Ahire, D. PRF as a Pulpotomy Medicament in a Permanent Molar with Pulpitis: A Case Report. *IOSR Journal of Dental and Medical Sciences (IOSR-JDMS) e-ISSN* **2014**, *13*, 5–09.
4. Arslan, H.; Şahin, Y.; Topçuoğlu, H.S.; Gündoğdu, B. Histologic Evaluation of Regenerated Tissues in the Pulp Spaces of Teeth with Mature Roots at the Time of the Regenerative Endodontic Procedures. *J Endod* **2019**, *45*, 1384–1389, doi:10.1016/j.joen.2019.07.016.
5. Lokade, J.; Chande, K.P.; Manwar, N.U. Retreatment of a Mutilated Tooth with Open Apex by Using PRF, MTA and Anatomic Post. *International Journal of Prosthodontics and Restorative Dentistry* **2013**, *3*, 105–110, doi:10.5005/jp-journals-10019-1087.
6. Kottoor, J.; Velmurugan, N. Revascularization for a Necrotic Immature Permanent Lateral Incisor: A Case Report and Literature Review. *Int J Paediatr Dent* **2013**, *23*, 310–316, doi:10.1111/ipd.12000.
7. Adhikari, H. Das; Gupta, A. Report of a Case of Platelet-Rich Fibrin-Mediated Revascularization of Immature 12 with Histopathological Evaluation. *J Conserv Dent* **2018**, *21*, 691–695, doi:10.4103/JCD.JCD_286_18.
8. Ari, G.; Kumar, A.K.; Ramakrishnan, T. Treatment of an Intrabony Defect Combined with an Endodontic Lesion: A Case Report. *Endo-Endodontic Practice Today* **2010**, *4*, 215–222.
9. Aunmeungtong, W.; Krongbaramee, T.; Khongkhunthian, P. Endodontic Management of a Chronic Periapical Abscess in a Maxillary Central Incisor with an Immature Root Apex Using Platelet-Rich Fibrin: A Case Report. *Eur Endod J* **2018**, *3*, 192–196, doi:10.14744/eej.2018.19483.
10. Bains, R.; Bains, V.K.; Loomba, K.; Verma, K.; Nasir, A. Management of Pulpal Floor Perforation and Grade II Furcation Involvement Using Mineral Trioxide Aggregate and Platelet Rich Fibrin: A Clinical Report. *Contemp Clin Dent* **2012**, *3*, S223-7, doi:10.4103/0976-237X.101100.
11. Bambal, D.; Manwar, N.U.; Chandak, M.; Rudagi, K. A Comparative Evaluation of the Healing Ability of Bilateral Periapical Lesions Treated with and without the Use of Platelet-Rich Fibrin. *Todays FDA* **2012**, *24*, 54–57.
12. Bilginaylar, K. Uncommon Odontogenic Orocutaneous Fistula of the Jaw Treated with Platelet-Rich Fibrin. *Case Rep Dent* **2017**, *2017*, 7174217, doi:10.1155/2017/7174217.
13. Kolcu, M.; Yurdakul, H.; Belli, S. Regenerative Endodontic Treatment of an Immature Incisor Tooth with a Novel Platelet-Rich Product: A Five-Year Follow-Up Case Report. *Niger J Clin Pract* **2023**, *26*, 1388–1392, doi:10.4103/njcp.njcp_747_22.
14. Chaudhari, P.; Chandak, M.; Chandak, M.; Sarangi, S.; Jaiswal, A.; Patel, A. Integrative Management of Open Apex with Regenerative Endodontic Therapy: A Case Report. *Med Sci* **2022**, *26*, doi:10.54905/disssi/v26i122/ms151e2124.
15. Córdova-Malca, F.; Coaguila-Llerena, H.; Garré-Arnillas, L.; Rayo-Iparraguirre, J.; Faria, G. Endodontic Micro-Resurgery and Guided Tissue Regeneration of a Periapical Cyst Associated to Recurrent Root Perforation: A Case Report. *Restor Dent Endod* **2022**, *47*, doi:10.5395/rde.2022.47.e35.
16. Das, S.; Srivastava, R.; Thosar, N.R.; Khubchandani, M.; Ragit, R.; Malviya, N. Regenerative Endodontics-Reviving the Pulp the Natural Way: A Case Report. *Cureus* **2023**, *15*, doi:10.7759/cureus.36587.
17. Das, A.N.; Geetha, K.; Kurian, A.V.; Nair, R.; Nandakumar, K. Interdisciplinary Approach to a Tooth with Open Apex and Persistent Sinus. *Case Rep Dent* **2015**, *2015*, doi:10.1155/2015/907324.
18. Demiralp, B.; Keçeli, H.G.; Muhtaroğullari, M.; Serper, A.; Eratalay, K. Treatment of Periapical Inflammatory Lesion with the Combination of Platelet-Rich Plasma and Tricalcium Phosphate: A Case Report. *J Endod* **2004**, *30*, 796–800, doi:10.1097/01.don.0000136211.98434.66.

19. Demir, B.; Demiralp, B.; Güncü, G.N.; Özgür Uyanik, M.; Çağlayan, F. Intentional Replantation of a Hopeless Tooth with the Combination of Platelet Rich Plasma, Bioactive Glass Graft Material and Non-Resorbable Membrane: A Case Report. *Dental Traumatology* **2007**, *23*, 190–194, doi:10.1111/j.1600-9657.2005.00414.x.
20. Deshpande, N.M.; Shah, D.; Wadekar, S. Maintenance of Cell Viability in Extraoral Conditions for a Case of Intentional Replantation to Retrieve a Separated Endodontic Instrument. *J Conserv Dent* **2019**, *22*, 207–212, doi:10.4103/JCD.JCD_461_18.
21. Dhirawani, V. Salvaging the Mutilated Internally Resorbed and Perforated Maxillary Incisor-One Year Six Months Follow-Up. *JOURNAL OF CLINICAL AND DIAGNOSTIC RESEARCH* **2016**, *10*, ZJ08–ZJ09, doi:10.7860/JCDR/2016/19333.7859.
22. Dudeja, P.G.; Dudeja, K.K.; Garg, A.; Srivastava, D.; Grover, S. Management of a Previously Treated, Calcified, and Dilacerated Maxillary Lateral Incisor: A Combined Nonsurgical/Surgical Approach Assisted by Cone-Beam Computed Tomography. *J Endod* **2016**, *42*, 984–988, doi:10.1016/j.joen.2016.03.020.
23. Faizuddin, U.; Solomon, R.V.; Mattapathi, J.; Guniganti, S.S. Revitalization of Traumatized Immature Tooth with Platelet-Rich Fibrin. *Contemp Clin Dent* **2015**, *6*, 574–576, doi:10.4103/0976-237X.169858.
24. Gaviño Orduña, J.F.; García García, M.; Dominguez, P.; Caviedes Bucheli, J.; Martin Biedma, B.; Abella Sans, F.; Manzanares Céspedes, M.C. Successful Pulp Revascularization of an Autotransplantated Mature Premolar with Fragile Fracture Apicoectomy and Plasma Rich in Growth Factors: A 3-year Follow-up. *Int Endod J* **2020**, *53*, 421–433, doi:10.1111/iej.13230.
25. Geethapriya, N.; Parthasarathy, R.; Mary, N.S.G.P.; Venkatesh, A. Review on Platelet Rich Fibrin and Case Report on Management of Large Periapical Lesion. *Indian J Public Health Res Dev* **2019**, *10*, 3064–3069, doi:10.5958/0976-5506.2019.04377.8.
26. Ghandi, M.; Ghorbani, F.; Sanaei-Rad, P. Management of Contralateral Molar Teeth with Necrotic Pulp and Open Apexes Using Platelet-rich Plasma and Vital Pulp Therapy. *Clin Case Rep* **2023**, *11*, doi:10.1002/ccr3.7230.
27. Goel, S.; Nawal, R.R.; Talwar, S. Management of Dens Invaginatus Type II Associated with Immature Apex and Large Periradicular Lesion Using Platelet-Rich Fibrin and Biodentine. *J Endod* **2017**, *43*, 1750–1755, doi:10.1016/j.joen.2017.04.005.
28. Govindaraju, L.; Antony, D.P.; S, P. Surgical Management of Radicular Cyst With the Application of a Natural Platelet Concentrate: A Case Report. *Cureus* **2023**, *15*, doi:10.7759/cureus.33992.
29. Goyal, L. Clinical Effectiveness of Combining Platelet Rich Fibrin with Alloplastic Bone Substitute for the Management of Combined Endodontic Periodontal Lesion. *Restor Dent Endod* **2014**, *39*, 51, doi:10.5395/rde.2014.39.1.51.
30. Gupta, B.; Gupta, S.; Wadhwa, J.; Gupta, A. Effect of Trauma to Primary Tooth on Permanent Maxillary Incisors: A Clinical Case Report. *Journal of Clinical and Diagnostic Research* **2017**, *11*, ZD06–ZD08, doi:10.7860/JCDR/2017/23935.9382.
31. Gupta, G.; Agarwal, A.; Ansari, A.A.; Singh, R.K. Non-Surgical Management of a Large Periapical Lesion with Internal Resorption Using PRF, Hydroxyapatite and MTA. *BMJ Case Rep* **2022**, *15*, e248907, doi:10.1136/bcr-2022-248907.
32. Güven Polat, G.; Yıldırım, C.; Akgün, Ö.M.; Altun, C.; Dinçer, D.; Özkan, C.K. The Use of Platelet Rich Plasma in the Treatment of Immature Tooth with Periapical Lesion: A Case Report. *Restor Dent Endod* **2014**, *39*, 230, doi:10.5395/rde.2014.39.3.230.
33. Hiremath, H.; Gada, N.; Kini, Y.; Kulkarni, S.; Yakub, S.S.; Metgud, S. Single-Step Apical Barrier Placement in Immature Teeth Using Mineral Trioxide Aggregate and Management of Periapical Inflammatory Lesion Using Platelet-Rich Plasma and Hydroxyapatite. *J Endod* **2008**, *34*, 1020–1024, doi:10.1016/j.joen.2008.05.004.
34. Hiremath, H.; Motiwala, T.; Jain, P.; Kulkarni, S. Use of Second-Generation Platelet Concentrate (Platelet-Rich Fibrin) and Hydroxyapatite in the Management of Large Periapical Inflammatory Lesion: A Computed Tomography Scan Analysis. *Indian J Dent Res* **2014**, *25*, 517–520, doi:10.4103/0970-9290.142556.
35. Hiremath, H.; Saikalyan, S.; Kulkarni, S.S.; Hiremath, V. Second-Generation Platelet Concentrate (PRF) as a Pulpotomy Medicament in a Permanent Molar with Pulpitis: A Case Report. *Int Endod J* **2012**, *45*, 105–112, doi:10.1111/j.1365-2591.2011.01973.x.

36. Jadhav, G.; Shah, D.; Raghvendra, S. Autologus Platelet Rich Fibrin Aided Revascularization of an Immature, Non-Vital Permanent Tooth with Apical Periodontitis: A Case Report. *J Nat Sci Biol Med* **2015**, *6*, 224, doi:10.4103/0976-9668.149187.
37. Jadhav, G.R.; Shah, N.; Logani, A. Platelet-Rich Plasma Supplemented Revascularization of an Immature Tooth Associated with a Periapical Lesion in a 40-Year-Old Man. *Case Rep Dent* **2014**, *2014*, 479584, doi:10.1155/2014/479584.
38. Johns, D.A.; Shivashankar, V.Y.; Krishnamma, S.; Johns, M. Use of Photoactivated Disinfection and Platelet-Rich Fibrin in Regenerative Endodontics. *J Conserv Dent* **2014**, *17*, 487–490, doi:10.4103/0972-0707.139850.
39. Johns, D.A.; Shivashankar, V.Y.; Maroli, R.K.; Vidyanath, S. Novel Management of Avulsed Tooth by Pulpal and Periodontal Regeneration. *J Endod* **2013**, *39*, 1658–1662, doi:10.1016/j.joen.2013.08.012.
40. Johns, D.; Shivashankar, V.; Maroli, R.; Joseph, R. Invasive Cervical Root Resorption: Engineering the Lost Tissue by Regeneration. *Contemp Clin Dent* **2013**, *4*, 536, doi:10.4103/0976-237X.123067.
41. Johns, D.A.; Vidyanath, S. Revitalization of Tooth with Necrotic Pulp and Open Apex by Using Platelet-Rich Plasma: A Case Report. *J Endod* **2011**, *37*, 743, doi:10.1016/j.joen.2011.03.018.
42. Jayalakshmi, K.B.; Agarwal, S.; Singh, M.P.; Vishwanath, B.T.; Krishna, A.; Agrawal, R. Platelet-Rich Fibrin with β-Tricalcium Phosphate-A Noval Approach for Bone Augmentation in Chronic Periapical Lesion: A Case Report. *Case Rep Dent* **2012**, *2012*, doi:10.1155/2012/902858.
43. Kanakamedala, A.; Ari, G.; Sudhakar, U.; Vijayalakshmi, R.; Ramakrishnan, T.; Emmadi, P. Treatment of a Furcation Defect with a Combination of Platelet-Rich Fibrin (PRF) and Bone Graft - a Case Report. *Endo-Endodontic Practice Today* **2009**, *3*, 127–135.
44. Kapoor, S. Surgical Management of a Non-Healing Intra-Alveolar Root Fracture Associated with Pulpal Calcification and Root Resorption: A Case Report. *Journal of clinical and diagnostic research* **2015**, *9*, ZD03–ZD05, doi:10.7860/JCDR/2015/12829.6046.
45. Kataria, V.; Sodhi, S.; Dogra, S.; Raju, V.G. Peri Radicular Cystic Enucleation and PRF Grafting Following Apicoectomy in Trichotillomania Patient- A Case Report. *Journal of Clinical and Diagnostic Research* **2020**, *14*, ZD01–ZD03, doi:10.7860/JCDR/2020/43455.14067.
46. Kavitha, M.; Krishnaveni, R.; Swathi, A.M.; M Abubacker, M.H. Evaluation of Healing by Cone Beam Computed Tomography (CBCT) Using Platelet-Rich Plasma (PRP) + β -Tricalcium Phosphate (β -TCP) and Platelet Rich Fibrin (PRF) + β -Tricalcium Phosphate (β - TCP) in Periapical Lesions: Case Report. *Niger J Clin Pract* **2020**, *23*, 1026–1029, doi:10.4103/njcp.njcp_54_20.
47. Keswani, D.; Pandey, R.K. Revascularization of an Immature Tooth with a Necrotic Pulp Using Platelet-Rich Fibrin: A Case Report. *Int Endod J* **2013**, *46*, 1096–1104, doi:10.1111/iej.12107.
48. Khetarpal, A.; Chaudhry, S.; Talwar, S.; Verma, M. Endodontic Management of Open Apex Using MTA and Platelet - Rich Fibrin Membrane Barrier: A Newer Matrix Concept. *J Clin Exp Dent* **2013**, *5*, e291-4, doi:10.4317/jced.51178.
49. Kumar, A.; Yadav, A.; Shetty, N. One-Step Apexification Using Platelet Rich Fibrin Matrix and Mineral Trioxide Aggregate Apical Barrier. *Indian J Dent Res* **2014**, *25*, 809–812, doi:10.4103/0970-9290.152210.
50. Kumar, J.K.; Surendranath, P.; Eswaramoorthy, R. Regeneration of Immature Incisor Using Platelet Rich Fibrin: Report of a Novel Clinical Application. *BMC Oral Health* **2023**, *23*, 69, doi:10.1186/s12903-023-02759-9.
51. Mandviwala, D.; Arora, A.; Kapoor, S.; Shah, P. Internal Root Resorption: A Rare Complication of Vital Pulp Therapy Using Platelet-Rich Fibrin. *Journal of Oral and Maxillofacial Pathology* **2022**, *26*, 132, doi:10.4103/jomfp.jomfp_389_21.
52. Martin, G.; Ricucci, D.; Gibbs, J.L.; Lin, L.M. Histological Findings of Revascularized/Revitalized Immature Permanent Molar with Apical Periodontitis Using Platelet-Rich Plasma. *J Endod* **2013**, *39*, 138–144, doi:10.1016/j.joen.2012.09.015.
53. Mayya, A.; Bhandary, S.; Kolakemar, A.; George, A.M. Management of Necrotic Immature Permanent Maxillary Incisors Using a Modified Technique of Revascularisation. *BMJ Case Rep* **2021**, *14*, doi:10.1136/bcr-2020-240203.
54. Meza, G.; Urrejola, D.; Saint Jean, N.; Inostroza, C.; López, V.; Khoury, M.; Brizuela, C. Personalized Cell Therapy for Pulpitis Using Autologous Dental Pulp Stem Cells and Leukocyte Platelet-Rich Fibrin: A Case Report. *J Endod* **2019**, *45*, 144–149, doi:10.1016/j.joen.2018.11.009.

55. Mishra, N.; Narang, I.; Mttal, N. Platelet-Rich Fibrin-Mediated Revitalization of Immature Necrotic Tooth. *Contemp Clin Dent* **2013**, *4*, 412–415, doi:10.4103/0976-237X.118379.
56. Mohan, A.; Krishnan, U.; Akber, M.; Nair, M.G.; Balan, A. Successful Management of a Case of True Radicular Dens Invaginatus Using Platelet-Rich Fibrin and Guided Tissue Regeneration. *Aust Endod J* **2020**, *46*, 94–100, doi:10.1111/aej.12377.
57. Nagaveni, N.B.; Poornima, P.; Joshi, J.S.; Pathak, S.; Nandini, D.B. Revascularization of Immature, Nonvital Permanent Tooth Using Platelet-Rich Fibrin in Children. *Pediatr Dent* **2015**, *37*, 1–6.
58. Nagaveni, N.B.; Pathak, S.; Poornima, P.; Joshi, J.S. Revascularization Induced Maturogenesis of Non-Vital Immature Permanent Tooth Using Platelet-Rich-Fibrin: A Case Report. *J Clin Pediatr Dent* **2016**, *40*, 26–30, doi:10.17796/1053-4628-40.1.26.
59. Nageh, M.; Ahmed, G.M.; El-Baz, A.A. Assessment of Regaining Pulp Sensibility in Mature Necrotic Teeth Using a Modified Revascularization Technique with Platelet-Rich Fibrin: A Clinical Study. *J Endod* **2018**, *44*, 1526–1533, doi:10.1016/j.joen.2018.06.014.
60. Nalawade, T.M.; N.L, C.; Arora, G.; Rachappa, M. Platelet Rich Plasma and Bone Graft for Rehabilitation of Luxation Injuries to Permanent Incisors. *J Adv Oral Res* **2011**, *2*, 41–44, doi:10.1177/2229411220110208.
61. Nosrat, A.; Bolhari, B.; Saber Tahan, S.; Dianat, O.; Dummer, P.M.H. Revitalizing Previously Treated Teeth with Open Apices: A Case Report and a Literature Review. *Int Endod J* **2021**, *54*, 1782–1793, doi:10.1111/iej.13570.
62. Parikh, B.; Navin, S.; Vaishali, P. A Comparative Evaluation of Healing with a Computed Tomography Scan of Bilateral Periapical Lesions Treated with and without the Use of Platelet-Rich Plasma. *Indian Journal of Dental Research* **2011**, *22*, 497, doi:10.4103/0970-9290.87087.
63. Parthasarathy, R.; Srinivasan, S.; C, V.; Thanikachalam, Y.; Ramachandran, A. An Interdisciplinary Management of Avulsed Maxillary Incisors: A Case Report. *Cureus* **2022**, *14*, doi:10.7759/cureus.23891.
64. Patel, G.K.; Deepika, P.C.; Sisodia, N.; Manjunath, M.K. Platelet Rich Fibrin in Management of Complex Endoperio Cases. *Kathmandu University Medical Journal* **2017**, *15*, 101–104.
65. Patel, G.K.; Gujjari, S.K.; Annapoorna, B.M.; Veerendra Kumar, S.C. Management of Chronic Luxated Central Incisor with Hopeless Prognosis. In *J Indian Soc Periodontol*; Department of Periodontology, JSS Dental College and Hospital, Mysore, Karnataka, India. India, 2013; Vol. 17, pp. 670–675 ISBN 0972-124X (Print) 0975-1580 (Electronic) 0972-124X (Linking).
66. Pawar, A.M.; Pawar, S.M.; Thakur, B.; Kokate, S. Successful Surgical Outcome for an Endodontic Failure Using Biodentine™ as Retrograde Restoration in Conjunction with Platelet Rich Fibrin (PRF) for Progressive Healing. *Journal of Clinical and Diagnostic Research* **2017**, *11*, ZJ03–ZJ04, doi:10.7860/JCDR/2017/25080.9688.
67. Pinto, N.; Harnish, A.; Cabrera, C.; Andrade, C.; Druttman, T.; Brizuela, C. An Innovative Regenerative Endodontic Procedure Using Leukocyte and Platelet-Rich Fibrin Associated with Apical Surgery: A Case Report. *J Endod* **2017**, *43*, 1828–1834, doi:10.1016/j.joen.2017.07.002.
68. Pradeep, K.; Kudva, A.; Narayanamoorthy, V.; Cariappa, K.; Saraswathi, Mv. Platelet-Rich Fibrin Combined with Synthetic Nanocrystalline Hydroxy Apatite Granules in the Management of Radicular Cyst. *Niger J Clin Pract* **2016**, *19*, 688, doi:10.4103/1119-3077.188711.
69. Prasad, J.; de Ataide, I. deNoronha; Chalakkal, P.; Likhyani, L. Comparison between the Outcomes of Two Platelet-Rich Concentrates on Apexogenesis in Young Permanent Incisors Requiring Endodontic Retreatment. *Contemp Clin Dent* **2018**, *9*, 156, doi:10.4103/ccd.ccd_9_18.
70. Priya M, H.; Tambakad, P.B.; Naidu, J. Pulp and Periodontal Regeneration of an Avulsed Permanent Mature Incisor Using Platelet-Rich Plasma after Delayed Replantation: A 12-Month Clinical Case Study. *J Endod* **2016**, *42*, 66–71, doi:10.1016/j.joen.2015.07.016.
71. Rahnama, M.; Kozicka-Czupkałło, M.; Czupkałło, Ł.; Jamrogiewicz, R. Surgical Treatment of Dentoalveolar Injury in the Anterior Region of Maxilla. *Current Issues in Pharmacy and Medical Sciences* **2013**, *26*, 401–405, doi:10.12923/j.2084-980X/26.4/a.11.
72. Ray Jr., H.L.; Marcelino, J.; Braga, R.; Horwat, R.; Lisien, M.; Khaliq, S. Long-Term Follow up of Revascularization Using Platelet-Rich Fibrin. *Dental Traumatology* **2016**, *32*, 80–84, doi:10.1111/edt.12189.

73. Rudagi, K.B.; Rudagi, B. One-Step Apexification in Immature Tooth Using Grey Mineral Trioxide Aggregate as an Apical Barrier and Autologus Platelet Rich Fibrin Membrane as an Internal Matrix. *J Conserv Dent* **2012**, *15*, 196–199, doi:10.4103/0972-0707.94582.
74. Sachdeva, G.S.; Sachdeva, L.T.; Goel, M.; Bala, S. Regenerative Endodontic Treatment of an Immature Tooth with a Necrotic Pulp and Apical Periodontitis Using Platelet-Rich Plasma (PRP) and Mineral Trioxide Aggregate (MTA): A Case Report. *Int Endod J* **2015**, *48*, 902–910, doi:10.1111/iej.12407.
75. Sakthivel, S.; Gayathri, V.; Anirudhan, S.; Roja, R.J.S. Platelet-Rich Fibrin and Collagen Matrix for the Regeneration of Infected Necrotic Immature Teeth. *J Clin Transl Res* **2020**, *6*, 1, doi:10.18053/jctres.06.202001.001.
76. Satheesh, S.L.; Jain, S.; Bhuyan, A.C.; Devi, L.S. Surgical Management of a Separated Endodontic Instrument Using Second Generation Platelet Concentrate and Hydroxyapatite. *Journal of Clinical and Diagnostic Research* **2017**, *11*, ZD01–ZD03, doi:10.7860/JCDR/2017/25761.9991.
77. Sharma, S.; Grover, S.; Dudeja, P.; Sharma, V.; Passi, D. Non-Surgical Management of Teeth with Wide Open Apices and Large Periapical Lesions: A Conservative Reality. *Journal of Clinical and Diagnostic Research* **2016**, *10*, ZJ01–ZJ02, doi:10.7860/JCDR/2016/21520.8765.
78. Shivashankar, V.Y.; Johns, D.A.; Vidyanath, S.; Kumar, M.R. Platelet Rich Fibrin in the Revitalization of Tooth with Necrotic Pulp and Open Apex. *J Conserv Dent* **2012**, *15*, 395–398, doi:10.4103/0972-0707.101926.
79. Johns, D.; Vidyanath, S.; Sam, G.; Shivashankar, V. Combination of Platelet Rich Fibrin, Hydroxyapatite and PRF Membrane in the Management of Large Inflammatory Periapical Lesion. *Journal of Conservative Dentistry* **2013**, *16*, 261, doi:10.4103/0972-0707.111329.
80. Shubhashini, N.; Kumar, R. V; Shija, A.S.; Razvi, S. Platelet-Rich Fibrin in Treatment of Periapical Lesions: A Novel Therapeutic Option. *Chin J Dent Res* **2013**, *16*, 79–82.
81. Singh, S. Management of an Endo Perio Lesion in a Maxillary Canine Using Platelet-Rich Plasma Concentrate and an Alloplastic Bone Substitute. In *J Indian Soc Periodontol*; Graded Specialist (Periodontology), 320 Field Hospital, C/O 99 APO, Pin - 903320, India. India, 2009; Vol. 13, pp. 97–100 ISBN 0975-1580 (Electronic) 0972-124X (Print) 0972-124X (Linking).
82. Solomon, R.; Faizuddin, U.; Guniganti, S.; Waghray, S. Analysis of the Rate of Maturogenesis of a Traumatized Cvek's Stage 3 Anterior Tooth Treated with Platelet-Rich Fibrin as a Regenerative Tool Using Three-Dimensional Cone-Beam Computed Tomography: An Original Case Report. *Indian Journal of Dental Research* **2015**, *26*, 90, doi:10.4103/0970-9290.156823.
83. Subash, D.; Shoba, K.; Aman, S.; Bharkavi, S.K.I. Revitalization of an Immature Permanent Mandibular Molar with a Necrotic Pulp Using Platelet-Rich Fibrin: A Case Report. *Journal of Clinical and Diagnostic Research* **2016**, *10*, ZD21–ZD23, doi:10.7860/JCDR/2016/21793.8902.
84. Teja, K.; Ramesh, S. Nonsurgical Management of Strip Perforation Using Platelet-Rich Fibrin and MTA by Matrix Concept – A Case Report with One Year Follow-Up. *Contemp Clin Dent* **2021**, *12*, 84, doi:10.4103/ccd.ccd_392_20.
85. Torabinejad, M.; Faras, H. A Clinical and Histological Report of a Tooth with an Open Apex Treated with Regenerative Endodontics Using Platelet-Rich Plasma. *J Endod* **2012**, *38*, 864–868, doi:10.1016/j.joen.2012.03.006.
86. Torabinejad, M.; Turman, M. Revitalization of Tooth with Necrotic Pulp and Open Apex by Using Platelet-Rich Plasma: A Case Report. *J Endod* **2011**, *37*, 265–268, doi:10.1016/j.joen.2010.11.004.
87. Vidhale, G.; Jain, D.; Jain, S.; Godhane, A. V; Pawar, G.R. Management of Radicular Cyst Using Platelet-Rich Fibrin & Iliac Bone Graft - A Case Report. *Journal of Clinical and Diagnostic Research* **2015**, *9*, ZD34–ZD36, doi:10.7860/JCDR/2015/13368.6136.
88. Viganò, R.; Disconzi, M.; Bertini, E.; Viganò, L.; Casu, C. B.P.F.C.® Bio-Plasma® with Pure Growth Factors (BioPlasma®) Used for the Treatment of a Persistent Great Periapical Lesion of an Endodontically Treated Tooth: A New Therapeutic Option. *Case Rep Dent* **2020**, *2020*, 1–6, doi:10.1155/2020/4876437.
89. Wadhwa, J.; Gupta, A.; Hans, S. Evaluation of Periapical Healing of Apicomarginal Defect in Mandibular First Molar Treated with Platelet Rich Fibrin: A Case Report. *Journal of Clinical and Diagnostic Research* **2017**, *11*, ZD01–ZD03, doi:10.7860/JCDR/2017/23992.9561.
90. Wang, Y.; Zhu, X.; Zhang, C. Pulp Revascularization on Permanent Teeth with Open Apices in a Middle-Aged Patient. *J Endod* **2015**, *41*, 1571–1575, doi:10.1016/j.joen.2015.04.022.

91. Yadav, P.; Pruthi, P.J.; Naval, R.R.; Talwar, S.; Verma, M. Novel Use of Platelet-rich Fibrin Matrix and <scp>MTA</Scp> as an Apical Barrier in the Management of a Failed Revascularization Case. *Dental Traumatology* **2015**, *31*, 328–331, doi:10.1111/edt.12168.
92. Yang, Y.; Zhang, B.; Huang, C.; Ye, R. Intentional Replantation of a Second Premolar with Internal Resorption and Root Fracture: A Case Report. *J Contemp Dent Pract* **2021**, *22*, 562–567, doi:10.5005/jp-journals-10024-3087.
93. Advancements in Regenerative Endodontics:Platelet-Rich Plasma(PRP) and Platelet –Rich Fibrin(PRF). Available online: https://www.researchgate.net/publication/332631728_Advancements_in_regenerative_endodonticsPlatelet-rich_plasmaPRP_and_platelet_-rich_fibrinPRF (accessed on 31 May 2025).
94. He, L.; Kim, S.G.; Gong, Q.; Zhong, J.; Wang, S.; Zhou, X.; Ye, L.; Ling, J.; Mao, J.J. Regenerative Endodontics for Adult Patients. *J Endod* **2017**, *43*, S57–S64, doi:10.1016/j.joen.2017.06.012.
95. Geisler, T.M. Clinical Considerations for Regenerative Endodontic Procedures. *Dent Clin North Am* **2012**, *56*, 603–626, doi:10.1016/j.cden.2012.05.010.
96. Platelet Concentrates: A Promising Innovation In ... - Ssdctumkur.Org Available online: https://www.yumpu.com/en/document/view/31297865/platelet-concentrates-a-promising-innovation-in-ssdctumkurorg (accessed on 1 June 2025).
97. Saoud, T.; Ricucci, D.; Lin, L.; Gaengler, P. Regeneration and Repair in Endodontics—A Special Issue of the Regenerative Endodontics—A New Era in Clinical Endodontics. *Dent J (Basel)* **2016**, *4*, 3, doi:10.3390/dj4010003.
98. Tabatabayi, M.H.; Ameghani, B.A.; Tavakoli, A. Review Article-Healing Process of Pulp Regeneration Using Bioactive Materials: A Review. *Adv. Biores* **2017**, *8*, 209–214, doi:10.15515/abr.0976-4585.8.3.209214.
99. Borie, E.; Oliví, D.G.; Orsi, I.A.; Garlet, K.; Weber, B.; Beltrán, V.; Fuentes, R. Platelet-Rich Fibrin Application in Dentistry: A Literature Review. *Int J Clin Exp Med* **2015**, *8*, 7922.
100. Namour, M.; Theys, S. Pulp Revascularization of Immature Permanent Teeth: A Review of the Literature and a Proposal of a New Clinical Protocol. *The Scientific World Journal* **2014**, *2014*, 1–9, doi:10.1155/2014/737503.
101. Bansal, R. Regenerative Endodontics: A Road Less Travelled. *JOURNAL OF CLINICAL AND DIAGNOSTIC RESEARCH* **2014**, *8*, doi:10.7860/JCDR/2014/8257.5034.
102. Diogenes, A.; Ruparel, N.B. Regenerative Endodontic Procedures. *Dent Clin North Am* **2017**, *61*, 111–125, doi:10.1016/j.cden.2016.08.004.
103. Lin, L.M.; Ricucci, D.; Huang, G.T. -J. Regeneration of the Dentine–Pulp Complex with Revitalization/Revascularization Therapy: Challenges and Hopes. *Int Endod J* **2014**, *47*, 713–724, doi:10.1111/iej.12210.
104. Velmurugan, N. Revascularization of Necrotic Immature Permanent Teeth: An Update. *Journal of Operative Dentistry & Endodontics* **2016**, *1*, 18–24, doi:10.5005/jp-journals-10047-0006.
105. Aksel, H.; Serper, A. Recent Considerations in Regenerative Endodontic Treatment Approaches. *J Dent Sci* **2014**, *9*, 207–213, doi:10.1016/j.jds.2013.12.007.
106. Alsolaihim, A.; Alsolaihim, A.; Alsolaihim, N.; Alowais, L. Biomimetic Regenerative Materials in Restorative Dentistry and Endodontics. *Journal of International Oral Health* **2023**, *15*, 250, doi:10.4103/jioh.jioh_162_22.
107. Araújo, L.; Goulart, T.S.; Gil, A.C.K.; Schuldt, D.P. V; Coelho, B.S.; Figueiredo, D.R.; Garcia, L.D.F.R.; Almeida, J. Do Alternative Scaffolds Used in Regenerative Endodontics Promote Better Root Development than That Achieved with Blood Clots? *Braz Dent J* **2022**, *33*, 22–32, doi:10.1590/0103-6440202204746.
108. Bezgin, T.; Sönmez, H. Review of Current Concepts of Revascularization/Revitalization. *Dental Traumatology* **2015**, *31*, 267–273, doi:10.1111/edt.12177.
109. Bolhari, B.; Meraji, N.; Ghorbanzadeh, A.; Sarraf, P.; Moayeri, R. Applications of Fibrin-Based Products in Endodontics: A Literature Review. *Dent Hypotheses* **2019**, *10*, 85, doi:10.4103/denthyp.denthyp_23_19.
110. Cecerska-Heryć, E.; Goszka, M.; Serwin, N.; Roszak, M.; Grygorcewicz, B.; Heryć, R.; Dołęgowska, B. Applications of the Regenerative Capacity of Platelets in Modern Medicine. *Cytokine Growth Factor Rev* **2022**, *64*, 84–94, doi:10.1016/j.cytogfr.2021.11.003.
111. Deepak, S.; Nivedhitha, M.S. Clinical Practice and Guidelines and Protocols for Revascularization Procedure - A Review. *Journal of Pharmaceutical Sciences and Research* **2017**, *9*, 2089–2092.

112. Del Fabbro, M.; Lolato, A.; Bucchi, C.; Taschieri, S.; Weinstein, R.L. Autologous Platelet Concentrates for Pulp and Dentin Regeneration: A Literature Review of Animal Studies. *J Endod* **2016**, *42*, 250–257, doi:10.1016/j.joen.2015.10.012.
113. Ducret, M.; Costantini, A.; Gobert, S.; Farges, J.-C.; Bekhouche, M. Fibrin-Based Scaffolds for Dental Pulp Regeneration: From Biology to Nanotherapeutics. *Eur Cell Mater* **2021**, *41*, 1–14, doi:10.22203/eCM.v041a01.
114. Elver, A.; Caymaz, M.G. Novel Approaches to the Use of Platelet-Rich Fibrin: A Literature Review. *Saudi Dent J* **2023**, *35*, 797–802, doi:10.1016/j.sdentj.2023.07.008.
115. Farhad, A.R.; Shokraneh, A.; Shekarchizade, N. Regeneration or Replacement? A Case Report and Review of Literature. *Dental Traumatology* **2016**, *32*, 71–79, doi:10.1111/edt.12200.
116. Yazid, F. Scaffold Selection for Tissue Engineering in Dentistry. *Med Health* **2020**, *15*, 34–53, doi:10.17576/MH.2020.1501.04.
117. Feigin, K.; Shope, B. Regenerative Endodontics. *J Vet Dent* **2017**, *34*, 161–178, doi:10.1177/0898756417722022.
118. Garrido-Parada, S.; Castelo-Baz, P.; Feijoo-Pato, N.; Gaviño-Orduña, J.; Martín-Biedma, B. Endodontic Regenerative Procedures in Necrotic Adult Teeth. *Applied Sciences* **2022**, *12*, 4212, doi:10.3390/app12094212.
119. Gathani, K.; Raghavendra, S. Scaffolds in Regenerative Endodontics: A Review. *Dent Res J (Isfahan)* **2016**, *13*, 379, doi:10.4103/1735-3327.192266.
120. George, R. Quality of Techniques Used to Assess Clinical Outcomes of Regenerative Endodontic Treatment in Necrotic Mature Teeth. *Evid Based Dent* **2022**, *23*, 98–99, doi:10.1038/s41432-022-0806-1.
121. Gong, T.; Heng, B.C.; Lo, E.C.M.; Zhang, C. Current Advance and Future Prospects of Tissue Engineering Approach to Dentin/Pulp Regenerative Therapy. *Stem Cells Int* **2016**, *2016*, doi:10.1155/2016/9204574.
122. Güven, E.P.; Karapınar-Kazandağ, M.; Tanalp, J. Revascularization: A Review of Clinical Reports on a Contemporary Treatment Modality for Endodontics. *Biomedical Research (India)* **2017**, *28*, 644–656.
123. Hameed, M.H.; Gul, M.; Ghafoor, R.; Badar, S.B. Management of Immature Necrotic Permanent Teeth with Regenerative Endodontic Procedures - a Review of Literature. *J Pak Med Assoc* **2019**, *69*, 1514–1520, doi:10.5455/JPMA.294366.
124. Hotwani, K.; Sharma, K. Platelet Rich Fibrin - a Novel Acumen into Regenerative Endodontic Therapy. *Restor Dent Endod* **2014**, *39*, 1, doi:10.5395/rde.2014.39.1.1.
125. Kim, S.G.; Malek, M.; Sigurdsson, A.; Lin, L.M.; Kahler, B. Regenerative Endodontics: A Comprehensive Review. *Int Endod J* **2018**, *51*, 1367–1388, doi:10.1111/iej.12954.
126. Gunasekaran, S.; Sakthivel, S.; M., S.B.; Babu, G.; Vijayan, V. Clinical Application of Platelet-Rich Fibrin in Pediatric Dentistry. *Journal of Health and Allied Sciences NU* **2022**, *12*, 186–190, doi:10.1055/s-0041-1736269.
127. Kim, S.; Shin, S.-J.; Song, Y.; Kim, E. *In Vivo* Experiments with Dental Pulp Stem Cells for Pulp-Dentin Complex Regeneration. *Mediators Inflamm* **2015**, *2015*, doi:10.1155/2015/409347.
128. Kontakiotis, E.G.; Filippatos, C.G.; Tzanetakis, G.N.; Agrafioti, A. Regenerative Endodontic Therapy: A Data Analysis of Clinical Protocols. *J Endod* **2015**, *41*, 146–154, doi:10.1016/j.joen.2014.08.003.
129. Krupińska, A.M.; Skośkiewicz-Malinowska, K.; Staniowski, T. Different Approaches to the Regeneration of Dental Tissues in Regenerative Endodontics. *Applied Sciences* **2021**, *11*, 1699, doi:10.3390/app11041699.
130. Kumar, N.; Maher, N.; Amin, F.; Ghabbani, H.; Zafar, M.S.; Rodríguez-Lozano, F.J.; Oñate-Sánchez, R.E. Biomimetic Approaches in Clinical Endodontics. *Biomimetics* **2022**, *7*, 229, doi:10.3390/biomimetics7040229.
131. Lim, G.S.; Wey, M.C.; Azami, N.H.; Noor, N.S.M.; Lau, M.N.; Haque, N.; Govindasamy, V.; Kasim, N.H.A. From Endodontic Therapy to Regenerative Endodontics: New Wine in Old Bottles. *Curr Stem Cell Res Ther* **2021**, *16*, 577–588, doi:10.2174/1574888X15999201116162256.
132. Lei, L.; Chen, Y.; Zhou, R.; Huang, X.; Cai, Z. Histologic and Immunohistochemical Findings of a Human Immature Permanent Tooth with Apical Periodontitis after Regenerative Endodontic Treatment. *J Endod* **2015**, *41*, 1172–1179, doi:10.1016/j.joen.2015.03.012.
133. Kahler, B.; Lin, L.M. A Review of Regenerative Endodontics: Current Protocols and Future Directions. *J Istanb Univ Fac Dent* **2017**, *51*, S41–S51, doi:10.17096/jiufd.53911.
134. Liu, H.; Lu, J.; Jiang, Q.; Haapasalo, M.; Qian, J.; Tay, F.R.; Shen, Y. Biomaterial Scaffolds for Clinical Procedures in Endodontic Regeneration. *Bioact Mater* **2022**, *12*, 257–277, doi:10.1016/j.bioactmat.2021.10.008.

135. Murray, P.E. Review of Guidance for the Selection of Regenerative Endodontics, Apexogenesis, Apexification, Pulpotomy, and Other Endodontic Treatments for Immature Permanent Teeth. *Int Endod J* **2023**, *56*, 188–199, doi:10.1111/iej.13809.
136. Nath, M.; Govind, S.; Jena, S.P. Platelet Rich Fibrin(Prf)-a Novel Generation of Regeneration in Endodontics: A Review. *Indian Journal of Forensic Medicine and Toxicology* **2021**, *15*, 3938–3943, doi:10.37506/ijfmt.v15i2.14988.
137. Noohi, P.; Abdekhodaie, M.J.; Nekoofar, M.H.; Galler, K.M.; Dummer, P.M.H. Advances in Scaffolds Used for <scp>pulp–Dentine</Scp> Complex Tissue Engineering: A Narrative Review. *Int Endod J* **2022**, *55*, 1277–1316, doi:10.1111/iej.13826.
138. Onicas, M.I.; Narita, L.E.; Mester, A.; Onisor, F.; Mancini, L. Platelet-Rich Fibrin: A Viable Therapy for Endodontic-Periodontal Lesions? A Preliminary Assessment. *Applied Sciences* **2021**, *11*, 7081, doi:10.3390/app11157081.
139. Pietruszka, P.; Chruścicka, I.; Duś-Ilnicka, I.; Paradowska-Stolarz, A. PRP and PRF—Subgroups and Divisions When Used in Dentistry. *J Pers Med* **2021**, *11*, 944, doi:10.3390/jpm11100944.
140. Pulyodan, M.K.; Mohan, S.P.; Valsan, D.; Divakar, N.; Moyin, S.; Thayylli, S. Regenerative Endodontics: A Paradigm Shift in Clinical Endodontics. *J Pharm Bioallied Sci* **2020**, *12*, 20–26, doi:10.4103/jpbs.JPBS_112_20.
141. Raddall, G.; Mello, I.; Leung, B.M. Biomaterials and Scaffold Design Strategies for Regenerative Endodontic Therapy. *Front Bioeng Biotechnol* **2019**, *7*, doi:10.3389/fbioe.2019.00317.
142. Rebentish, P.D.; Umashetty, G.; Kaur, H.; Doizode, T.; Kaslekar, M.; Chowdhury, S. Platelet-Rich Fibrin: A Boon in Regenerative Endodontics. *Minerva Stomatol* **2016**, *65*, 385–392.
143. Riaz, A.; Shah, F.A. Regenerating the Pulp–Dentine Complex Using Autologous Platelet Concentrates: A Critical Appraisal of the Current Histological Evidence. *Tissue Eng Regen Med* **2021**, *18*, 37–48, doi:10.1007/s13770-020-00291-3.
144. Sandra, F.; Sutanto, A.; Wulandari, W.; Lambertus, R.; Celinna, M.; Dewi, N.M.; Ichwan, S.J.A. Crucial Triad in Pulp-Dentin Complex Regeneration: Dental Stem Cells, Scaffolds, and Signaling Molecules. *The Indonesian Biomedical Journal* **2023**, *15*, 25–46, doi:10.18585/inabj.v15i1.2265.
145. Schmalz, G.; Widbiller, M.; Galler, K.M. Signaling Molecules and Pulp Regeneration. *J Endod* **2017**, *43*, S7–S11, doi:10.1016/j.joen.2017.06.003.
146. Smith, A.J.; Duncan, H.F.; Diogenes, A.; Simon, S.; Cooper, P.R. Exploiting the Bioactive Properties of the Dentin-Pulp Complex in Regenerative Endodontics. *J Endod* **2016**, *42*, 47–56, doi:10.1016/j.joen.2015.10.019.
147. Solete, P.; Vijaya, P.P. Advances in Pulp Capping Agents. *International Journal of Pharmaceutical and Clinical Research* **2014**, *6*, 320–323.
148. Staffoli, S.; Plotino, G.; Torrijos, B.G.N.; Grande, N.M.; Bossu, M.; Gambarini, G.; Polimeni, A. Regenerative Endodontic Procedures Using Contemporary Endodontic Materials. *MATERIALS* **2019**, *12*, doi:10.3390/ma12060908.
149. Sun, H.-H.; Jin, T.; Yu, Q.; Chen, F.-M. Biological Approaches toward Dental Pulp Regeneration by Tissue Engineering. *J Tissue Eng Regen Med* **2011**, *5*, e1–e16, doi:10.1002/term.369.
150. Umakanth, K.; Balaji Ganesh, S.; Smiline Girija, A. Applications Of Platelet Concentrates In Endodontics - A Review. *International Journal of Pharmaceutical Research* **2020**, *12*, 2102–2107, doi:10.31838/ijpr/2020.SP2.225.
151. Vijayalakshmi, B.; Pradeep, S. Role of Platelet Rich Fibrin in Dentistry. *Res J Pharm Technol* **2016**, *9*, 2037–2040, doi:10.5958/0974-360X.2016.00416.9.
152. Wei, X.; Yang, M.; Yue, L.; Huang, D.; Zhou, X.; Wang, X.; Zhang, Q.; Qiu, L.; Huang, Z.; Wang, H.; et al. Expert Consensus on Regenerative Endodontic Procedures. *Int J Oral Sci* **2022**, *14*, 55, doi:10.1038/s41368-022-00206-z.
153. Xu, J.; Gou, L.; Zhang, P.; Li, H.; Qiu, S. Platelet-rich Plasma and Regenerative Dentistry. *Aust Dent J* **2020**, *65*, 131–142, doi:10.1111/adj.12754.
154. Zbańska, J.; Herman, K.; Kuropka, P.; Dobrzyński, M. Regenerative Endodontics as the Future Treatment of Immature Permanent Teeth. *Applied Sciences* **2021**, *11*, 6211, doi:10.3390/app11136211.
155. Zein, N.; Harmouch, E.; Lutz, J.-C.; Fernandez De Grado, G.; Kuchler-Bopp, S.; Clauss, F.; Offner, D.; Hua, G.; Benkirane-Jessel, N.; Fioretti, F. Polymer-Based Instructive Scaffolds for Endodontic Regeneration. *Materials* **2019**, *12*, 2347, doi:10.3390/ma12152347.

156. Zhou, C.; Yuan, Z.; Xu, H.; Wu, L.; Xie, C.; Liu, J. Regenerative Endodontic Procedures in Immature Permanent Teeth With Dental Trauma: Current Approaches and Challenges. *Frontiers in Dental Medicine* **2021**, *2*, 767226, doi:10.3389/FDMED.2021.767226/BIBTEX.
157. Zoltowska, A.; Machut, K.; Pawlowska, E.; Derwich, M. Plasma Rich in Growth Factors in the Treatment of Endodontic Periapical Lesions in Adult Patients: A Narrative Review. *Pharmaceuticals* **2021**, *14*, 1041, doi:10.3390/ph14101041.
158. Monga, P.; Grover, R.; Mahajan, P.; Keshav, V.; Singh, N.; Singh, G. A Comparative Clinical Study to Evaluate the Healing of Large Periapical Lesions Using Platelet-Rich Fibrin and Hydroxyapatite. *Endodontology* **2016**, *28*, 27, doi:10.4103/0970-7212.184336.
159. Rastanawi, D.; Al-Halabiah Assistant, H.; Dentistry, O.; Al-Halabiah, H. Clinical and Radiographic Study to Investigate the Effect of Platelet-Rich Fibrin Membranes (PRF) Application as Partial Pulpotomy Agent. *International Journal of Applied Dental Sciences* **2020**, *6*, 74–79.
160. Wagih Tarek Ali; Jealan M. El-Shafei; Moushira Dahaba; Alaa El Baz Evaluation of Survival of Mature Second Premolar with Periapical Lesion Following Different Regenerative Treatment Protocols: A Randomized Controlled Trial. *Indian J Public Health Res Dev* **2021**, *12*, 533–543, doi:10.37506/IJPHRD.V12I3.16123.
161. Evaluation_of_healing_after_periapical_surgery.6., doi:10.4103/endo.endo_55_17.
162. Mohamed Mohsen Abielhassan; Nihal Ezzat Sabet; Alaa Abdelsalam El Baz Assessment of the Survival and Sensibility of Mature Anterior Teeth with Periapical Lesion after One Step Regenerative Approach Using Different Disinfection Maneuvers : A Randomized Clinical Trial. *Indian J Public Health Res Dev* **2021**, *12*, 416–425, doi:10.37506/ijphrd.v12i3.16093.
163. Abo-Heikal, M.M.; El-Shafei, J.M.; Shouman, S.A.; Roshdy, N.N. Evaluation of the Efficacy of Injectable Platelet-rich Fibrin versus Platelet-rich Plasma in the Regeneration of Traumatized Necrotic Immature Maxillary Anterior Teeth: A Randomized Clinical Trial. *Dental Traumatology* **2024**, *40*, 61–75, doi:10.1111/edt.12881.
164. Ahmed, G.M.; Nageh, M.; Ei-Baz, A.A.; Saif, N.; Moustafa, O.H.M.S. CBCT Volumetric Evaluation of Bone Healing after Endodontic Microsurgery Using Platelet-Rich Fibrin (PRF). *Endo-endodontic practice today* **2018**, *12*, 241–248.
165. Ahmed, Y.E.; Ahmed, G.M.; Ghoneim, A.G. Evaluation of Postoperative Pain and Healing Following Regenerative Endodontics Using Platelet-rich Plasma versus Conventional Endodontic Treatment in Necrotic Mature Mandibular Molars with Chronic Periapical Periodontitis. A Randomized Clinical Trial. *Int Endod J* **2023**, *56*, 404–418, doi:10.1111/iej.13886.
166. Alagl, A.; Bedi, S.; Hassan, K.; AlHumaid, J. Use of Platelet-Rich Plasma for Regeneration in Non-Vital Immature Permanent Teeth: Clinical and Cone-Beam Computed Tomography Evaluation. *Journal of International Medical Research* **2017**, *45*, 583–593, doi:10.1177/0300060517692935.
167. Alawwad, M.; Altinawi, M.; Rekab, M.S.; Kosyreva, T.; Almokaddam, H.; Katbeh, I. A Comparative Clinical Radiological Study Using Platelet Rich Fibrin and MTA in Pulpotomy of First Permanent Immature Molars. *Journal of Clinical and Diagnostic Research* **2020**, *14*, ZC01–ZC05, doi:10.7860/JCDR/2020/45490.14198.
168. Alawwad, M.; Altinawi, M.; Rekab, M.S.; Kosyreva, T.; Almokaddam, H.; Katbeh, I. A Randomised Clinical Radiological Study Using Platelet Rich Fibrin and MTA in Pulpotomy of First Permanent Immature Molars. *Journal of Clinic and Diagnostic Research* **2020**, *14*, ZC01–ZC05, doi:10.7860/JCDR/2020/45877.14178.
169. A Comparative Clinical Radiological Study between Using Formocresol, MTA and Platelet Rich Fibrin (PRF) in Pulpotomy of Second Primary Molars (Irreversible Pulpitis) Available online: https://www.discoveryjournals.org/medicalscience/current_issue/v25/n117/A13.htm (accessed on 31 May 2025).
170. Ali, W.T.; Shafie, J.E.; Dahaba, M.; Baz, A.E. Evaluation of Survival of Mature Second Premolar with Periapical Lesion Following Different Regenerative Treatment Protocols: A Randomized Controlled Trial. *Indian J Public Health Res Dev* **2021**, *12*, 533–543, doi:10.37506/ijphrd.v12i3.16123.
171. Angerame, D.; De Biasi, M.; Kastrioti, I.; Franco, V.; Castaldo, A.; Maglione, M. Application of Platelet-Rich Fibrin in Endodontic Surgery: A Pilot Study. *G Ital Endod* **2015**, *29*, 51–57, doi:10.1016/j.gien.2015.08.003.
172. Bezgin, T.; Yilmaz, A.D.; Celik, B.N.; Kolsuz, M.E.; Sonmez, H. Efficacy of Platelet-Rich Plasma as a Scaffold in Regenerative Endodontic Treatment. *J Endod* **2015**, *41*, 36–44, doi:10.1016/j.joen.2014.10.004.

173. Makkad, R.S. Platelet-Rich Fibrin and Titanium-Prepared Platelet-Rich Fibrin in Endoperio Lesion Management. *Bioinformation* **2023**, *19*, 133–137, doi:10.6026/97320630019133.
174. Devi Praja, V.; Muttath, A.; Duraisamy, V.; Selvarajan, N.; Suresh Kumar, V.; John, J. A Clinical and Radiographic Comparison of Platelet-Rich Fibrin and Lyophilized Platelet-Derived Preparation as Pulpotomy Agent in Primary Molars. *J Pharm Bioallied Sci* **2020**, *12*, 155, doi:10.4103/jpbs.JPBS_48_20.
175. Dhamija, R.; Tewari, S.; Sangwan, P.; Duhan, J.; Mittal, S. Impact of Platelet Rich Plasma in the Healing of Through-and-through Periapical Lesions Using 2-D and 3-D Evaluation: A Randomized Controlled Trial. *J Endod* **2020**.
176. Dhiman, M.; Kumar, S.; Duhan, J.; Sangwan, P.; Tewari, S. Effect of Platelet-Rich Fibrin on Healing of Apicomarginal Defects: A Randomized Controlled Trial. *J Endod* **2015**, *41*, 985–991, doi:10.1016/j.joen.2015.04.004.
177. Eid, A.; Mancino, D.; Rekab, M.S.; Haikel, Y.; Kharouf, N. Effectiveness of Three Agents in Pulpotomy Treatment of Permanent Molars with Incomplete Root Development: A Randomized Controlled Trial. *Healthcare* **2022**, *10*, 431, doi:10.3390/healthcare10030431.
178. Elkholly, A.; Negm, M.; Hassan, R.; Omar, N. Healing Assessment of Osseous Defects after Surgical Removal of Periapical Lesions in the Presence of Hydroxyapatite, Nanohydroxyapatite, and a Combination of Nanohydroxyapatite and Platelet-Rich Fibrin: A Clinical Study. *Open Access Maced J Med Sci* **2022**, *10*, 406–414, doi:10.3889/OAMJMS.2022.10766.
179. ElSheshtawy, A.S.; Nazzal, H.; El Shahawy, O.I.; El Baz, A.A.; Ismail, S.M.; Kang, J.; Ezzat, K.M. The Effect of Platelet-Rich Plasma as a Scaffold in Regeneration/Revitalisation Endodontics of Immature Permanent Teeth Assessed Using 2-Dimensional Radiographs and Cone Beam Computed Tomography: A Randomised Controlled Trial. *Int Endod J* **2020**.
180. Garg, M.; Srivastava, V.; Chauhan, R.; Pramanik, S.; Khanna, R. Application of Platelet-Rich Fibrin and Freeze-Dried Bone Allograft Following Apicoectomy. *Indian Journal of Dental Research* **2023**, *34*, 40–44, doi:10.4103/ijdr.ijdr_810_22.
181. Goyal, B.; Tewari, S.; Duhan, J.; Sehgal, P.K. Comparative Evaluation of Platelet-Rich Plasma and Guided Tissue Regeneration Membrane in the Healing of Apicomarginal Defects: A Clinical Study. *J Endod* **2011**, *37*, 773–780, doi:10.1016/j.joen.2011.03.003.
182. Gupta, S.; Mittal, N.; Baranwal, H.; Rath, C.; Shankari, T.; Gupta, S. Comparative Evaluation of Bioglass Nanofiber, Dexamethasone-Coated Bioglass Nanofiber, and Platelet-Rich Fibrin, as Scaffolds in Regenerative Endodontic Treatment of Immature Necrotic Teeth: A Randomized Controlled Trial. *Journal of Conservative Dentistry* **2022**, *25*, 561, doi:10.4103/jcd.jcd_264_22.
183. Jadhav, G.; Shah, N.; Logani, A. Revascularization with and without Platelet-Rich Plasma in Nonvital, Immature, Anterior Teeth: A Pilot Clinical Study. *J Endod* **2012**, *38*, 1581–1587, doi:10.1016/j.joen.2012.09.010.
184. Jayadevan, V.; Gehlot, P.M.; Manjunath, V.; Madhunapantula, S. V.; Lakshmikanth, J.S.D. A Comparative Evaluation of Advanced Platelet-Rich Fibrin (A-PRF) and Platelet-Rich Fibrin (PRF) as a Scaffold in Regenerative Endodontic Treatment of Traumatized Immature Non-Vital Permanent Anterior Teeth: A Prospective Clinical Study. *J Clin Exp Dent* **2021**, *13*, 463–472, doi:10.4317/JCED.57902,.
185. Johri, S.; Verma, P.; Tikku, A.P.; Bains, R.; Kohli, N. Effect of Amniotic Membrane and Platelet-Rich Fibrin Membrane on Bone Healing Post Endodontic Surgery: An Ultrasonographic, Randomized Controlled Study. *J Tissue Eng Regen Med* **2022**, *16*, 1208–1222, doi:10.1002/term.3362.
186. Karan, N.B.; Aricioğlu, B. Assessment of Bone Healing after Mineral Trioxide Aggregate and Platelet-Rich Fibrin Application in Periapical Lesions Using Cone-Beam Computed Tomographic Imaging. *Clin Oral Investig* **2020**, *24*, 1065–1072, doi:10.1007/s00784-019-03003-x.
187. Kavitha, M.; Sivaprakasam, S.P.; Arunaraj, D.; Hemamalini, R.; Velayudham, S.; Bakthavatchalam, B. Comparative Evaluation of Platelet-Rich Fibrin and Concentrated Growth Factor as Scaffolds in Regenerative Endodontic Procedure: A Randomized Controlled Clinical Trial. *J Contemp Dent Pract* **2023**, *23*, 1211–1217, doi:10.5005/jp-journals-10024-3443.
188. Keswani, D.; Pandey, R.K.; Ansari, A.; Gupta, S. Comparative Evaluation of Platelet-Rich Fibrin and Mineral Trioxide Aggregate as Pulpotomy Agents in Permanent Teeth with Incomplete Root Development: A Randomized Controlled Trial. *J Endod* **2014**, *40*, 599–605, doi:10.1016/j.joen.2014.01.009.

189. Kumar, V.; Juneja, R.; Duhan, J.; Sangwan, P.; Tewari, S. Comparative Evaluation of Platelet-Rich Fibrin, Mineral Trioxide Aggregate, and Calcium Hydroxide as Pulpotomy Agents in Permanent Molars with Irreversible Pulpitis: A Randomized Controlled Trial. *Contemp Clin Dent* **2016**, *7*, 512, doi:10.4103/0976-237X.194107.
190. Manhas, M.; Mittal, S.; Sharma, A.; Gupta, K.; Pathania, V.; Thakur, V. Biological Approach in Repair of Partially Inflamed Dental Pulp Using Second-Generation Platelet-Rich Fibrin and Mineral Trioxide Aggregate as a Pulp Medicament in Primary Molars. *Journal of Indian Society of Pedodontics and Preventive Dentistry* **2019**, *37*, 399, doi:10.4103/JISPPD.JISPPD_133_19.
191. Markandey, S.; Das Adhikari, H. Evaluation of Blood Clot, Platelet-Rich Plasma, and Platelet-Rich Fibrin-Mediated Regenerative Endodontic Procedures in Teeth with Periapical Pathology: A CBCT Study. *Restor Dent Endod* **2022**, *47*, e41–e41, doi:10.5395/rde.2022.47.e41.
192. Meschi, N.; EzEldeen, M.; Garcia, A.E.T.; Lahoud, P.; Van Gorp, G.; Coucke, W.; Jacobs, R.; Vandamme, K.; Teughels, W.; Lambrechts, P. Regenerative Endodontic Procedure of Immature Permanent Teeth with Leukocyte and Platelet Rich Fibrin: A Multicenter Controlled Clinical Trial. *J Endod* **2021**, doi:10.1016/j.joen.2021.08.003.
193. Meschi, N.; Fieuws, S.; Vanhoenacker, A.; Strijbos, O.; Van der Veken, D.; Politis, C.; Lambrechts, P. Root-End Surgery with Leucocyte- and Platelet-Rich Fibrin and an Occlusive Membrane: A Randomized Controlled Clinical Trial on Patients' Quality of Life. *Clin Oral Investig* **2018**, *22*, 2401–2411, doi:10.1007/s00784-018-2343-z.
194. Meschi, N.; Vanhoenacker, A.; Strijbos, O.; Camargo Dos Santos, B.; Rubbers, E.; Peeters, V.; Curvers, F.; Van Mierlo, M.; Geukens, A.; Fieuws, S.; et al. Multi-Modular Bone Healing Assessment in a Randomized Controlled Clinical Trial of Root-End Surgery with the Use of Leukocyte- and Platelet-Rich Fibrin and an Occlusive Membrane. *Clin Oral Investig* **2020**, *24*, 4439-4453, doi:10.1007/s00784-020-03309-1.
195. Mittal, N.; Parashar, V. Regenerative Evaluation of Immature Roots Using PRF and Artificial Scaffolds in Necrotic Permanent Teeth: A Clinical Study. *J Contemp Dent Pract* **2019**, *20*, 720–726, doi:10.5005/jp-journals-10024-2586.
196. Mohammed, S.E.; Gawdat, S.I.; Ibrahim, S.M. Role Of Prf With Mta And Theracal After Pulpotomy In Relieving Pain And Maintaining The Vitality Of The Remaining Radicular Pulp Tissue In Permanent Posterior Teeth With Closed Root Apices: "Randomized Controlled Trial." *J Pharm Negat Results* **2022**, *13*, 2559–2569, doi:10.47750/pnr.2022.13.S05.396.
197. Nageh, M.; Ibrahim, L.; Salam, E. Outcome of PRF Pulpotomy Using Different Calcium-Silicate Based Materials in Permanent Molars with Irreversible Pulpitis: A Randomized Controlled Trial. *J Dent Res* **2019**, *98*.
198. Narang, I.; Mittal, N.; Mishra, N. A Comparative Evaluation of the Blood Clot, Platelet-Rich Plasma, and Platelet-Rich Fibrin in Regeneration of Necrotic Immature Permanent Teeth: A Clinical Study. *Contemp Clin Dent* **2015**, *6*, 63, doi:10.4103/0976-237X.149294.
199. Naik, S. V; Attiguppe, P.; Prakash, A.J. Comparative Evaluation of the Regenerative Potential of Blood Clot and Platelet-Rich Fibrin in Young Permanent Teeth Based on the Revised American Academy of Endodontics Clinical Considerations for Regenerative Procedure: 2016. *Int J Clin Pediatr Dent* **2023**, *16*, S149–S154, doi:10.5005/jp-journals-10005-2654.
200. Patidar, S.; Kalra, N.; Khatri, A.; Tyagi, R. Clinical and Radiographic Comparison of Platelet-Rich Fibrin and Mineral Trioxide Aggregate as Pulpotomy Agents in Primary Molars. *Journal of Indian Society of Pedodontics and Preventive Dentistry* **2017**, *35*, 367, doi:10.4103/JISPPD.JISPPD_178_17.
201. Ragab, R.A.; Lattif, A.E.A. El; Dokky, N.A.E.W. El Comparative Study between Revitalization of Necrotic Immature Permanent Anterior Teeth with and without Platelet Rich Fibrin: A Randomized Controlled Trial. *Journal of Clinical Pediatric Dentistry* **2019**, *43*, 78–85, doi:10.17796/1053-4625-43.2.2.
202. Ramachandran, N.; Singh, S.; Podar, R.; Kulkarni, G.; Shetty, R.; Chandrasekhar, P. A Comparison of Two Pulp Revascularization Techniques Using Platelet-Rich Plasma and Whole Blood Clot. *Journal of Conservative Dentistry* **2020**, *23*, 637, doi:10.4103/JCD.JCD_221_20.
203. Razi, M.A.; Mahajan, A.; Qamar, S.; Mehra, S.; Roy, T.R.; Kumari, P. A Comparative Study of Platelet-Rich Fibrin (PRF) and Titanium-Prepared Platelet-Rich Fibrin (T-PRF) in Management of Endo-Perio Lesions. *J Contemp Dent Pract* **2020**, *21*, 997–1001, doi:10.5005/jp-journals-10024-2865.
204. Rizk, H.M.; Al-Deen, M.S.S.; Emam, A.A. Regenerative Endodontic Treatment of Bilateral Necrotic Immature Permanent Maxillary Central Incisors with Platelet-Rich Plasma versus Blood Clot: A Split Mouth Double-

Blinded Randomized Controlled Trial. *Int J Clin Pediatr Dent* **2019**, *12*, 332-339, doi:10.5005/jp-journals-10005-1656.

205. Rizk, H.M.; Salah Al-Deen, M.S.M.; Emam, A.A. Comparative Evaluation of Platelet Rich Plasma (PRP) versus Platelet Rich Fibrin (PRF) Scaffolds in Regenerative Endodontic Treatment of Immature Necrotic Permanent Maxillary Central Incisors: A Double Blinded Randomized Controlled Trial. *Saudi Dent J* **2020**, *32*, 224–231, doi:10.1016/j.sdentj.2019.09.002.
206. Sallam, N.M.; El Kalla, I.H.; Wahba, A.H.; Salama, N.M. Clinical and Radiographic Evaluation of Platelet-Rich Fibrin for Revascularization of Necrotic Immature Permanent Teeth: A Controlled Clinical Trial. *Pediatric Dental Journal* **2020**, *30*, 182–190, doi:10.1016/j.pdj.2020.09.001.
207. Santhakumar, M.; Yayathi, S.; Retnakumari, N. A Clinicoradiographic Comparison of the Effects of Platelet-Rich Fibrin Gel and Platelet-Rich Fibrin Membrane as Scaffolds in the Apexification Treatment of Young Permanent Teeth. *Journal of Indian Society of Pedodontics and Preventive Dentistry* **2018**, *36*, 65, doi:10.4103/JISPPD.JISPPD_180_17.
208. Shivashankar, V.Y. Comparison of the Effect of PRP, PRF and Induced Bleeding in the Revascularization of Teeth with Necrotic Pulp and Open Apex: A Triple Blind Randomized Clinical Trial. *Journal of Clinic and Diagnostic Research* **2017**, *11*, ZC34–ZC39, doi:10.7860/JCDR/2017/22352.10056.
209. Shobana, S.; Kavitha, M.; Srinivasan, N. Efficacy of Platelet Rich Plasma and Platelet Rich Fibrin for Direct Pulp Capping in Adult Patients with Carious Pulp Exposure- A Randomised Controlled Trial. *Eur Endod J* **2022**, *7*, 114–121, doi:10.14744/eej.2021.04834.
210. Gupta, S.; Gupta, P.; Raman, N.; Singh, A.; Shah, A.; Ramola, V. Comparison between Different Combinations of Alendronate, Platelet-Rich Fibrin, Hydroxyapatite in Bone Regeneration in Endodontic Surgeries Using Cone-Beam Computed Tomography. *J Contemp Dent Pract* **2022**, *23*, 337–342, doi:10.5005/jp-journals-10024-3312.
211. Singh, R.; Ismail, P.M.S.; Kambli, V.; Singh, K.D. Evaluation of Hydroxyapatite Granules, CERAMENT&Trade;, and Platelet-Rich Fibrin in the Management of Endodontic Apical Surgery. *J Contemp Dent Pract* **2020**, *21*, 554–557, doi:10.5005/jp-journals-10024-2825.
212. Thakur, V.; Mittal, S.; Tewari, S.; Kamboj, M.; Duhan, J.; Sangwan, P.; Kumar, V.; Gupta, A. Comparative Histological Evaluation of Two PRF Formulations (PRF High and PRF Medium) on Quality of Life and Healing Outcome of Apicomarginal Defects: A Randomized Clinical Trial. *Journal of Cranio-Maxillofacial Surgery* **2023**, *51*, 166–177, doi:10.1016/j.jcms.2023.02.004.
213. Tiwari, U.; Chandra, R.; Tripathi, S.; Jain, J.; Jaiswal, S.; Tiwari, R. Comparative Analysis of Platelet.Rich Fibrin, Platelet-Rich Fibrin with Hydroxyapatite and Platelet-Rich Fibrin with Alendronate in Bone Regeneration: A Cone-Beam Computed Tomography Analysis. *Journal of Conservative Dentistry* **2020**, *23*, 348, doi:10.4103/JCD.JCD_228_20.
214. Ulusoy, A.T.; Turedi, I.; Cimen, M.; Cehreli, Z.C. Evaluation of Blood Clot, Platelet-Rich Plasma, Platelet-Rich Fibrin, and Platelet Pellet as Scaffolds in Regenerative Endodontic Treatment: A Prospective Randomized Trial. *J Endod* **2019**, *45*, 560–566, doi:10.1016/j.joen.2019.02.002.
215. Uppala, S. A Comparative Evaluation of PRF, Blood Clot and Collagen Scaffold in Regenerative Endodontics. *European Journal of Molecular and Clinical Medicine* **2020**, *7*, 3401–3410.
216. Vaishnavi, C.; Mohan, B.; Narayanan, L.L. Treatment of Endodontically Induced Periapical Lesions Using Hydroxyapatite, Platelet-Rich Plasma, and a Combination of Both: An in Vivo Study. *J Conserv Dent* **2011**, *14*, 140–146, doi:10.4103/0972-0707.82614.
217. Wu, Z.; Lin, Y.; Xu, X.; Chen, Z.; Xiang, Y.; Yang, L.; Zhang, W.; Xiao, S.; Chen, X. Clinical Observation of Autologous Platelet Rich Fibrin Assisted Revascularization of Mature Permanent Teeth. *Head Face Med* **2023**, *19*, 9, doi:10.1186/s13005-023-00350-9.
218. Youssef, A.; Ali, M.; ElBolok, A.; Hassan, R. Regenerative Endodontic Procedures for the Treatment of Necrotic Mature Teeth: A Preliminary Randomized Clinical Trial. *Int Endod J* **2022**, *55*, 334–346, doi:10.1111/iej.13681.
219. Alawwad, M.; Altinawi, M.; Rekab, M.S.; Kosyreva, T.; Almokaddam, H.; Katbeh, I. A Randomised Clinical Radiological Study Using Platelet Rich Fibrin and MTA in Pulpotomy of First Permanent Immature Molars. *Journal of Clinical and Diagnostic Research* **2020**, doi:10.7860/JCDR/2020/45877.14178.

220. Chi, C.I. A Clinical Study of Effect of Platelet-Rich Preparations on Pulp Regeneration. *https://trialsearch.who.int/Trial2.aspx?TrialID=ChiCTR-INR-17010425* **2017**.
221. Ctri A Clinical Trial Comparing Different Materials in Treatment Invloving Incompletely Formed Teeth. *https://trialsearch.who.int/Trial2.aspx?TrialID=CTRI/2022/11/047698* **2022**.
222. Ctri A Comparison Of Single Vs Multiple Visit Regeneration Therapy, on Different Platforms. *https://trialsearch.who.int/Trial2.aspx?TrialID=CTRI/2019/03/018162* **2019**.
223. Pactr A Novel Way to Treat Immature Teeth. *https://trialsearch.who.int/Trial2.aspx?TrialID=PACTR201502001032409* **2015**.
224. Ctri A Randomized Control Clinical Trial to Evaluate the Efficacy of 2 Bio Resorbable Matrices in Apexification Procedure. *https://trialsearch.who.int/Trial2.aspx?TrialID=CTRI/2017/07/009029* **2017**.
225. Ctri Assessment of Bone Healing after Endodontic Surgery Using Two Bioresorbable Membranes. *https://trialsearch.who.int/Trial2.aspx?TrialID=CTRI/2020/12/029599* **2020**.
226. Ctri Assessment of Healing of Periapical Lesions by Surgical and Non Surgical Methods. *https://trialsearch.who.int/Trial2.aspx?TrialID=CTRI/2023/02/049775* **2023**.
227. Nct Assessment of Regenerative Potential of Mature Permanent Teeth With Necrotic Pulps Using Two Revascularization Protocols. (In Vivo Study). *https://clinicaltrials.gov/show/NCT04158232* **2019**.
228. Nct Cell-Free Autologous Regenerative Endodontics Treatment for Teeth With Periapical Lesions (CARETT). *https://clinicaltrials.gov/show/NCT05305417* **2022**.
229. Nct Clinical and Radiographic Assessment of Platelet Rich Fibrin and Mineral Trioxide Aggregate as Pulp Capping Biomaterials. *https://clinicaltrials.gov/show/NCT04488679* **2020**.
230. Ctri Comparasion of Success Rate of Pulpotomy with Biodentine Using PRF Membrane and Collagen Scaffold in Permanent Molars. *https://trialsearch.who.int/Trial2.aspx?TrialID=CTRI/2023/03/050834* **2023**.
231. Ctri Comparative Study of Healing after Periapical Surgery BY ULTRASOUND Using Platelet Rich Fibrin. *https://trialsearch.who.int/Trial2.aspx?TrialID=CTRI/2017/08/009415* **2017**.
232. Ctri Comparision of Different Scaffolds for Success of Regeneration in Immature Apex. *https://trialsearch.who.int/Trial2.aspx?TrialID=CTRI/2021/07/034841* **2021**.
233. Ctri Comparison of Two Plasma Derivates as Pulpotomy Agents in Human Immature Molars. *https://trialsearch.who.int/Trial2.aspx?TrialID=CTRI/2020/06/025938* **2020**.
234. Ctri Comparison of Two Procedures for Regeneration of Dental Pulp in Teeth with Open Apex. *https://trialsearch.who.int/Trial2.aspx?TrialID=CTRI/2021/09/036778* **2021**.
235. Nct Diode Laser Pulpotomy of Mature Permanent Molars With Irreversible Pulpitis. *https://clinicaltrials.gov/show/NCT05427851* **2022**.
236. Nct Effectiveness of Mineral Trioxide Aggregate and Platelet Rich Fibrin Along With Biodentine. *https://clinicaltrials.gov/show/NCT04773886* **2021**.
237. Nct Effectiveness of Regenerative Endodontics Therapy for Single-Rooted Mature Permanent Tooth With Pulp Necrosis. *https://clinicaltrials.gov/show/NCT04313010* **2020**.
238. Ctri Effect of Platelet Rich Fibrin (PRF) in Formation of Tooth Apex in Cases of Immature Front Teeth. *https://trialsearch.who.int/Trial2.aspx?TrialID=CTRI/2012/11/003086* **2012**.
239. Nct Efficacy of Combined Scaffolds and Sodium Hypochlorite in Regenerative Endodontics of Immature Teeth. *https://clinicaltrials.gov/show/NCT05803525* **2023**.
240. Nct Effect of Platelet-Rich Plasma Biomembrane on Enhancing Bone Regeneration Following Endodontic Surgery. *https://clinicaltrials.gov/show/NCT04109417* **2019**.
241. Nct Efficacy of PRF and MTA as Compared to Calcium Hydroxide for Pulpotomy in Human Irreversibly Inflamed Permanent Teeth. *https://clinicaltrials.gov/show/NCT05266859* **2022**.
242. Nct Efficacy of Regenerative Endodontic Treatment With PRF as a Secondary Treatment of Mature Necrotic Incisors in Adolescents. *https://clinicaltrials.gov/show/NCT05517187* **2022**.
243. Nct Endodontic Microsurgery With the Use of L-PRF and an Occlusive Membrane: A Randomized Controlled Clinical Trial. *https://clinicaltrials.gov/show/NCT02528240* **2015**.
244. Ctri Evaluating Efficacy of PRF and Chorion Membrane in Regeneration. *https://trialsearch.who.int/Trial2.aspx?TrialID=CTRI/2022/04/042051* **2022**.

245. Ctri Evaluation of Bone Healing in Periapical Lesion by Using Bone Repair Material. *https://trialsearch.who.int/Trial2.aspx?TrialID=CTRI/2022/02/040568* **2022**.
246. Nct Evaluation Of Different Pulpotomy Agents Used For Treatment Of Immature Molars. *https://clinicaltrials.gov/show/NCT05347160* **2022**.
247. Tctr Evaluation of Endodontic Treatment Outcomes in Open Apex Permanent Teeth Using Rich-Platelet Fibrin and MTA. *https://trialsearch.who.int/Trial2.aspx?TrialID=TCTR20221109006* **2022**.
248. Pactr Evaluation of Platelet Rich Fibrin Regenerative Pulpotomy in Mature Permanent Molars with Irreversible Pulpitis. *https://trialsearch.who.int/Trial2.aspx?TrialID=PACTR202001824413147* **2019**.
249. Ctri Evaluation of Regenerative Procedures on Vital Mandibular Posterior Teeth. *https://trialsearch.who.int/Trial2.aspx?TrialID=CTRI/2020/12/029804* **2020**.
250. Nct Healing Potentiality Of Blood Clot ,S-PRF and A-PRF In Treatment Of Necrotic Mature Single Rooted Teeth With Chronic Peri-Apical Periodontitis. *https://clinicaltrials.gov/show/NCT04606719* **2020**.
251. Nct Pain After PRP Revascularization and Endodontic Treatment of Non-Vital Mandibular Molars With Apical Periodontitis. *https://clinicaltrials.gov/show/NCT03350841* **2017**.
252. Ctri Periapical Healing Evaluation after Root End Surgery with Different Retro Filling Materials with or without Platelet Rich Fibrin. *https://trialsearch.who.int/Trial2.aspx?TrialID=CTRI/2020/02/023443* **2020**.
253. Han, B.; Wang, Y.; Chen, Z.; Zheng, C.; Zhang, Z.; Liu, Y.; Liu, K.; Wang, Z.; Wang, X. Platelet-Rich Fibrin/Anorganic Bovine Bone Mineral Complex as Grafting Materials in Endodontic Microsurgery with a Large Lesion Size: Study Protocol for a Randomised Controlled Trial. *BMJ Open* **2022**, *12*, doi:10.1136/BMJOPEN-2021-057068,.
254. Nct Platelet Rich Fibrin(PRF) Injection for Treatment of Dental Pulp Exposure: Randomized Clinical Trial. *https://clinicaltrials.gov/show/NCT04330768* **2020**.
255. Nct Platelet Rich Plasma in Healing of Through and Through Periradicular Lesions. *https://clinicaltrials.gov/show/NCT03430557* **2018**.
256. Nct Post-Operative Evaluation of Endodontic Microsurgeries Done Using a Piezoelectric Ultrasonic Technique: An in Vivo Study. *https://clinicaltrials.gov/show/NCT05863728* **2023**.
257. Ctri Post Operative Pain after Non Surgical Treatment of Open Apex Maxillary Central Incisors. *https://trialsearch.who.int/Trial2.aspx?TrialID=CTRI/2023/05/053223* **2023**.
258. Nct PRF and Bone Graft in Endodontic Surgeries. *https://clinicaltrials.gov/show/NCT04377698* **2020**.
259. Nct PRF Pulpotomy Using Different Bioceramic Materials in Permanent Molars. *https://clinicaltrials.gov/show/NCT04784949* **2021**.
260. Ctri Pulp Capping Agent to Induce Calcific Bridge Formation. *https://trialsearch.who.int/Trial2.aspx?TrialID=CTRI/2021/09/036129* **2021**.
261. Ctri Pulpotomy in Mature Permanent Tooth Using Various Biomaterials. *https://trialsearch.who.int/Trial2.aspx?TrialID=CTRI/2023/06/053989* **2023**.
262. Nct Regenerative Endodontic Procedure of Immature Permanent Teeth With Apical Periodontitis Using PRF. *https://clinicaltrials.gov/show/NCT02801552* **2016**.
263. Pactr Saving Immature Teeth. *https://trialsearch.who.int/Trial2.aspx?TrialID=PACTR201304000530301* **2013**.
264. Ctri The Effect of Blood-Derived Products on Bone Healing after Surgery : A Randomized Clinical Trial. *https://trialsearch.who.int/Trial2.aspx?TrialID=CTRI/2023/10/058270* **2023**.
265. Nct The Use of Platelet-Rich Fibrin in Partial Pulpotomy Procedure. *https://clinicaltrials.gov/show/NCT04331964* **2020**.
266. Ctri To Analyze the Effect of Bone Healing in Periapical Defects Using L-PRF, Simvastatin, AND Amniotic Membrane as Bone Grafts by Clinicaly and Radiographicaly. *https://trialsearch.who.int/Trial2.aspx?TrialID=CTRI/2022/08/044782* **2022**.
267. Ctri To Study Effect of Patients Own Platelet Concentrate Harvested from Blood on Dental Pulp Preservation to Prevent Root Canal Treatment. *https://trialsearch.who.int/Trial2.aspx?TrialID=CTRI/2020/05/025052* **2020**.
268. Ctri Treatment of Permanent Teeth with Dead Pulp by a Technique to Renew Pulp to Regain Natural Defence Mechanism for Pulp. *https://trialsearch.who.int/Trial2.aspx?TrialID=CTRI/2021/09/036395* **2021**.

269. Ctri Comparasion of Success Rate of Pulpotomy with Biodentine Using PRF Membrane and Collagen Scaffold in Permanent Molars. *https://trialsearch.who.int/Trial2.aspx?TrialID=CTRI/2023/03/050834* **2023**.
270. Ctri Pulpotomy in Mature Permanent Tooth Using Various Biomaterials. *https://trialsearch.who.int/Trial2.aspx?TrialID=CTRI/2023/06/053989* **2023**.
271. Nct Evaluation of the Effect of Leukocyte and Platelet-Rich Fibrin on Healing After Periradicular Surgery. *https://clinicaltrials.gov/show/NCT05847647* **2023**.
272. Kiaipour, Z.; Shafiee, M.; Ansari, G. Role of Platelet Concentrates in Dental-Pulp Regeneration: A Systematic Review of Randomized Clinical Trials. *J Dent* **2024**, *25*, 97, doi:10.30476/DENTJODS.2023.96000.1912.
273. Lin, L.; Chen, M.Y.H.; Ricucci, D.; Rosenberg, P.A. Guided Tissue Regeneration in Periapical Surgery. *J Endod* **2010**, *36*, 618–625, doi:10.1016/j.joen.2009.12.012.
274. Alghamdi, F.T.; Alqurashi, A.E. Regenerative Endodontic Therapy in the Management of Immature Necrotic Permanent Dentition: A Systematic Review. *Scientific World Journal* **2020**, *2020*, doi:10.1155/2020/7954357.
275. Alghamdi, F.; Alsulaimani, M. Regenerative Endodontic Treatment: A Systematic Review of Successful Clinical Cases. *Dent Med Probl* **2021**, *58*, 555–567, doi:10.17219/dmp/132181.
276. Al-Haddad, A.Y.; Al-Namnam, N.M. Regenerative Endodontic Treatment in Mature Teeth: A Systematic Review and Meta-Analysis. *G Ital Endod* **2022**, *36*, 170–184, doi:10.32067/GIE.2021.35.02.51.
277. Digka, A.; Sakka, D.; Lyroudia, K. Histological Assessment of Human Regenerative Endodontic Procedures (<scp>REP</Scp>) of Immature Permanent Teeth with Necrotic Pulp/Apical Periodontitis: A Systematic Review. *Australian Endodontic Journal* **2020**, *46*, 140–153, doi:10.1111/aej.12371.
278. (PDF) Review Article Regenerative Endodontic Therapy: A Systematic Review of Clinical Protocols Available online: https://www.researchgate.net/publication/309782167_Review_Article_Regenerative_endodontic_therapy_a_systematic_review_of_clinical_protocols (accessed on 31 May 2025).
279. Chen, Y.; Chen, X.; Zhang, Y.; Zhou, F.; Deng, J.; Zou, J.; Wang, Y. Materials for Pulpotomy in Immature Permanent Teeth: A Systematic Review and Meta-Analysis. *BMC Oral Health* **2019**, *19*, 1–9, doi:10.1186/S12903-019-0917-Z/FIGURES/4.
280. Bucchi, C.; Arias, A.; Fuentes, R. Platelet Concentrates in the Replantation of Avulsed Teeth: A Systematic Review. *Int J Clin Exp Med* **2016**, *9*, 22985–22992.
281. Chisini, L.A.; Grazioli, G.; Francia, A.; Martin, A.S.S.; Demarco, F.F.; Conde, M.C.M. Revascularization versus Apical Barrier Technique with Mineral Trioxide Aggregate Plug: A Systematic Review. *G Ital Endod* **2018**, *32*, 9–16, doi:10.1016/J.GIEN.2018.03.006.
282. Dadpe, A.M. Regenerative Endodontic Procedures in Teeth with Root Resorption: A Systematic Review. *Eur Endod J* **2023**, *8*, 170–186, doi:10.14744/eej.2023.77486.
283. Glynis, A.; Foschi, F.; Kefalou, I.; Koletsi, D.; Tzanetakis, G.N. Regenerative Endodontic Procedures for the Treatment of Necrotic Mature Teeth with Apical Periodontitis: A Systematic Review and Meta-Analysis of Randomized Controlled Trials. *J Endod* **2021**, *47*, 873–882, doi:10.1016/J.JOEN.2021.03.015,.
284. di Lauro, A.E.; Valletta, A.; Aliberti, A.; Cangiano, M.; Dolce, P.; Sammartino, G.; Gasparro, R. The Effectiveness of Autologous Platelet Concentrates in the Clinical and Radiographic Healing after Endodontic Surgery: A Systematic Review. *Materials* **2023**, *16*, 7187, doi:10.3390/ma16227187.
285. Ayousha Iqbal; Amna Riaz; Alia Waheed; Safi Ullah Khan; Kanza Nawadat; Sadaf Islam Reorienting Goals in Endodontic Therapy: Pulp Revitalization, on the Brink of a Paradigm Shift. *J Pak Med Assoc* **2021**, *71*, 2589–2595, doi:10.47391/JPMA.01908.
286. Joshi, S.; Palekar, A.; Pendyala, G.; Mopagar, V.; Padmawar, N.; Shah, P. Clinical Success of Platelet-Rich Fibrin and Mineral Trioxide Aggregate (MTA) or MTA-like Agents in Healing of Periapical Lesion in Nonsurgically Treated Pulpless Immature Permanent Teeth: A Systematic Review. *J Int Soc Prev Community Dent* **2020**, *10*, 379–383, doi:10.4103/JISPCD.JISPCD_97_20,.
287. Khurshid, Z.; Asiri, F.Y.I.; Najeeb, S.; Ratnayake, J. The Impact of Autologous Platelet Concentrates on the Periapical Tissues and Root Development of Replanted Teeth: A Systematic Review. *Materials* **2022**, *15*, 2776, doi:10.3390/ma15082776.

288. Li, J.; Zheng, L.; Daraqel, B.; Liu, J.; Hu, Y. Treatment Outcome of Regenerative Endodontic Procedures for Necrotic Immature and Mature Permanent Teeth: A Systematic Review and Meta-Analysis Based on Randomised Controlled Trials. *Oral Health Prev Dent* **2023**, *21*, 141–152, doi:10.3290/j.ohpd.b4100877.
289. Liu, T.J.; Zhou, J.N.; Guo, L.H. Impact of Different Regenerative Techniques and Materials on the Healing Outcome of Endodontic Surgery: A Systematic Review and Meta-Analysis. *Int Endod J* **2021**, *54*, 536–555, doi:10.1111/iej.13440.
290. Lolato, A.; Bucchi, C.; Taschieri, S.; Kabbaney, A. El; Fabbro, M. Del Platelet Concentrates for Revitalization of Immature Necrotic Teeth: A Systematic Review of the Clinical Studies. *Platelets* **2016**, *27*, 383–392, doi:10.3109/09537104.2015.1131255,.
291. MacInnes, A. What Is the Most Effective Endodontic Medicament for Pulpotomies in Immature Permanent Teeth? *Evid Based Dent* **2020**, *21*, 108–109, doi:10.1038/s41432-020-0126-2.
292. Meschi, N.; Castro, A.B.; Vandamme, K.; Quirynen, M.; Lambrechts, P. The Impact of Autologous Platelet Concentrates on Endodontic Healing: A Systematic Review. *Platelets* **2016**, *27*, 613–633, doi:10.1080/09537104.2016.1226497,.
293. Metlerska, J.; Fagogeni, I.; Nowicka, A. Efficacy of Autologous Platelet Concentrates in Regenerative Endodontic Treatment: A Systematic Review of Human Studies. *J Endod* **2019**, *45*, 20–30, doi:10.1016/j.joen.2018.09.003.
294. Moraschini, V.; Miron, R.J.; Mourão, C.F. de A.B.; Louro, R.S.; Sculean, A.; da Fonseca, L.A.M.; Calasans Maia, M.D.; Shibli, J.A. Antimicrobial Effect of Platelet-rich Fibrin: A Systematic Review of in Vitro Evidence-based Studies. *Periodontol 2000* **2024**, *94*, 131–142, doi:10.1111/prd.12529.
295. Murray, P.E. Platelet-Rich Plasma and Platelet-Rich Fibrin Can Induce Apical Closure More Frequently Than Blood-Clot Revascularization for the Regeneration of Immature Permanent Teeth: A Meta-Analysis of Clinical Efficacy. *Front Bioeng Biotechnol* **2018**, *6*, doi:10.3389/fbioe.2018.00139.
296. Castro-Gutiérrez, M.E.M.; Argueta-Figueroa, L.; Fuentes-Mascorro, G.; Moreno-Rodríguez, A.; Torres-Rosas, R. Novel Approaches for the Treatment of Necrotic Immature Teeth Using Regenerative Endodontic Procedures: A Systematic Review and Meta-Analysis. *Applied Sciences (Switzerland)* **2021**, *11*, 5199, doi:10.3390/APP11115199/S1.
297. Nicoloso, G.F.; Pötter, I.G.; Rocha, R. de O.; Montagner, F.; Casagrande, L. A Comparative Evaluation of Endodontic Treatments for Immature Necrotic Permanent Teeth Based on Clinical and Radiographic Outcomes: A Systematic Review and Meta-Analysis. *Int J Paediatr Dent* **2017**, *27*, 217–227, doi:10.1111/IPD.12261,.
298. Noor Mohamed, R.; Basha, S.; Al-Thomali, Y. Efficacy of Platelet Concentrates in Pulpotomy–a Systematic Review. *Platelets* **2018**, *29*, 440–445, doi:10.1080/09537104.2018.1445844.
299. Oktawati, S.; Siswanto, H.; Mardiana, A.; Supiaty; Neormansyah, I.; Basir, I. Endodontic–Periodontic Lesion Management: A Systematic Review. *Medicina Clínica Práctica* **2020**, *3*, 100098, doi:10.1016/j.mcpsp.2020.100098.
300. Andrès, E.; Amato, M.; Pantaleo, G.; Iandolo, A.; Panda, P.; Mishra, L.; Govind, S.; Panda, S.; Lapinska, B. Clinical Outcome and Comparison of Regenerative and Apexification Intervention in Young Immature Necrotic Teeth—A Systematic Review and Meta-Analysis. *Journal of Clinical Medicine 2022, Vol. 11, Page 3909* **2022**, *11*, 3909, doi:10.3390/JCM11133909.
301. Pecci-Lloret, M.P.; Nandin-Muttoni, G.; Pecci-Lloret, M.R.; Guerrero-Gironés, J.; Rodríguez-Lozano, F.J. Scaffolds for Pulp Revitalisation: A Systematic Review of Randomized Clinical Trials. *Annals of Anatomy* **2022**, *243*, doi:10.1016/j.aanat.2022.151936.
302. Rahul, M.; Lokade, A.; Tewari, N.; Mathur, V.; Agarwal, D.; Goel, S.; Keshari, P.; Sharma, S.; Bansal, K. Effect of Intracanal Scaffolds on the Success Outcomes of Regenerative Endodontic Therapy - A Systematic Review and Network Meta-Analysis. *J Endod* **2023**, *49*, 110–128, doi:10.1016/j.joen.2022.11.011.
303. Ríos-Osorio, N.; Caviedes-Bucheli, J.; Jimenez-Peña, O.; Orozco-Agudelo, M.; Mosquera-Guevara, L.; Jiménez-Castellanos, F.; Muñoz-Alvear, H. Comparative Outcomes of Platelet Concentrates and Blood Clot Scaffolds for Regenerative Endodontic Procedures: A Systematic Review of Randomized Controlled Clinical Trials. *J Clin Exp Dent* **2023**, *15*, e239–e249, doi:10.4317/jced.60150.
304. Sabeti, M.; Ghobrial, D.; Zanjir, M.; da Costa, B.R.; Young, Y.; Azarpazhooh, A. Treatment Outcomes of Regenerative Endodontic Therapy in Immature Permanent Teeth with Pulpal Necrosis: A Systematic Review and Network Meta-analysis. *Int Endod J* **2024**, *57*, 238–255, doi:10.1111/iej.13999.

305. Hugar, S.M.; Gokhale, N.; Soneta, S.P.; Joshi, R.S.; Dialani, P.K.; Saxena, N. Evaluation of the Treatment Protocols in the Management of Pulpally Involved Young Permanent Teeth in Children: A Systematic Review and Meta-Analysis. *Int J Clin Pediatr Dent* **2022**, *15*, S103–S113, doi:10.5005/jp-journals-10005-2218.
306. Scelza, P.; Gonçalves, F.; Caldas, I.; Nunes, F.; Lourenço, E.S.; Tavares, S.; Magno, M.; Pintor, A.; Montemezzi, P.; Di Edoardo, E.; et al. Prognosis of Regenerative Endodontic Procedures in Mature Teeth: A Systematic Review and Meta-Analysis of Clinical and Radiographic Parameters. *Materials* **2021**, *14*, 4418, doi:10.3390/MA14164418/S1.
307. Shaik, I.; Gaddam, B.; Patel, A.; Deshmukh, R.; Bhavana, M.; Sunku, M.S.M.; Minnikanti, A. Success Rate of Growth Factors for Existing Periapical Lesions in Failed Endodontically Treated Teeth in Adult Population: A Systematic Review and Meta-Analysis. *J Pharm Bioallied Sci* **2022**, *14*, S200–S202, doi:10.4103/jpbs.jpbs-139-22.
308. Sinha, A.; Jain, A.K.; Rao, R.D.; Sivasailam, S.; Jain, R. Effect of Platelet-Rich Fibrin on Periapical Healing and Resolution of Clinical Symptoms in Patients Following Periapical Surgery: A Systematic Review and Meta-Analysis. *J Conserv Dent* **2023**, *26*, 366–376, doi:10.4103/jcd.jcd_195_23.
309. Tang, Q.; Jin, H.; Lin, S.; Ma, L.; Tian, T.; Qin, X. Are Platelet Concentrate Scaffolds Superior to Traditional Blood Clot Scaffolds in Regeneration Therapy of Necrotic Immature Permanent Teeth? A Systematic Review and Meta-Analysis. *BMC Oral Health* **2022**, *22*, doi:10.1186/S12903-022-02605-4,.
310. Tirez, E.; Pedano, M.S. Regeneration of the Pulp Tissue: Cell Homing versus Cell Transplantation Approach: A Systematic Review. *MATERIALS* **2022**, *15*, doi:10.3390/ma15238603.
311. Vatankhah, M.; Najary, S.; Dianat, O. Clinical, Radiographic, and Histologic Outcomes of Regenerative Endodontic Treatment in Human Immature Teeth Using Different Biological Scaffolds: A Systematic Review and Meta-Analysis. *Curr Stem Cell Res Ther* **2024**, *19*, 611–627, doi:10.2174/1574888X17666220903141155,.
312. Wikström, A.; Brundin, M.; Lopes, M.F.; El Sayed, M.; Tsilingaridis, G. What Is the Best Long-Term Treatment Modality for Immature Permanent Teeth with Pulp Necrosis and Apical Periodontitis? *European Archives of Paediatric Dentistry* **2021**, *22*, 311–340, doi:10.1007/S40368-020-00575-1,.
313. Ong, T.K.; Lim, G.S.; Singh, M.; Fial, A. V. Quantitative Assessment of Root Development after Regenerative Endodontic Therapy: A Systematic Review and Meta-Analysis. *J Endod* **2020**, *46*, 1856-1866.e2, doi:10.1016/j.joen.2020.08.016.
314. Alenazy, M.S.; Al-Nazhan, S.; Mosadomi, H.A. Histologic, Radiographic, and Micro-Computed Tomography Evaluation of Experimentally Enlarged Root Apices in Dog Teeth with Apical Periodontitis after Regenerative Treatment. *Curr Ther Res Clin Exp* **2021**, *94*, doi:10.1016/j.curtheres.2020.100620.
315. Y. Alhazzazi, T.; M. Rashed, F.; A. Matar, M.; F. Bogari, D.; F. Mounir, M.M. Regenerative Endodontic Therapy Using Platelet-Rich Plasma (PRP) and Amelogenin for the Treatment of Non-Vital Immature Permanent Teeth with Apical Periodontitis: An Animal Study. *J Pharm Res Int* **2021**, 326–334, doi:10.9734/jpri/2021/v33i49B33370.
316. Ameghani, B.A.; Tavakoli, A.; Tabatabaei, M.H.; Valizadeh, S. Effects of Mineral Trioxide Aggregate and Platelet-Rich Fibrin on Histological Results of Direct Pulp Capping in Dogs. *G Ital Endod* **2021**, *35*, 13–22, doi:10.32067/GIE.2021.35.01.01.
317. Behnaz, M.; Izadi, S.S.; Mashhadi Abbas, F.; Dianat, O.; Sadeghabadi, S.; Akbarzadeh, T.; Haeri, A.; Kazem, M.; Younessian, F. The Impact of Platelet-rich Fibrin (PRF) on Delayed Tooth Replantation: A Preliminary Animal Study. *Australian Endodontic Journal* **2021**, *47*, 457–466, doi:10.1111/aej.12492.
318. Chen, Y.J.; Zhao, Y.H.; Zhao, Y.J.; Liu, N.X.; Lv, X.; Li, Q.; Chen, F.M.; Zhang, M. Potential Dental Pulp Revascularization and Odonto-/Osteogenic Capacity of a Novel Transplant Combined with Dental Pulp Stem Cells and Platelet-Rich Fibrin. *Cell Tissue Res* **2015**, *361*, 439–455, doi:10.1007/s00441-015-2125-8.
319. Eldessoky, A.E.; Khalefa, M.M.; Abu-Seida, A.M. Regenerative Endodontic Therapy in Mature Teeth with Necrotic Pulp and Apical Periodontitis Using Two Disinfection Protocols. *BMC Oral Health* **2023**, *23*, 163, doi:10.1186/s12903-023-02863-w.
320. El Halaby, H.M.; Abu-Seida, A.M.; Fawzy, M.I.; Farid, M.H.; Bastawy, H.A. Evaluation of the Regenerative Potential of Dentin Conditioning and Naturally Derived Scaffold for Necrotic Immature Permanent Teeth in a Dog Model. *Int J Exp Pathol* **2020**, *101*, 264–276, doi:10.1111/IEP.12372;PAGE:STRING:ARTICLE/CHAPTER.

321. El Kalla, I.H.; Salama, N.M.; Wahba, A.H.; Sallam, N.M. Histological Evaluation of Platelet-Rich Fibrin for Revascularization of Immature Permanent Teeth in Dogs. *PEDIATRIC DENTAL JOURNAL* **2019**, *29*, 72–77, doi:10.1016/j.pdj.2019.04.004.
322. Ghoddusi, J.; Maghsudlu, A.; Jafarzadeh, H.; Jafarian, A.; Forghani, M. Histological Evaluation of the Effect of Platelet-Rich Plasma on Pulp Regeneration in Nonvital Open Apex Teeth: An Animal Study. *J Contemp Dent Pract* **2017**, *18*, 1045–1050, doi:10.5005/jp-journals-10024-2173.
323. Gomes-Filho, J.E.; Duarte, P.C.; Ervolino, E.; Mogami Bomfim, S.R.; Xavier Abimussi, C.J.; Mota da Silva Santos, L.; Lodi, C.S.; Penha De Oliveira, S.H.; Dezan, E.; Cintra, L.T. Histologic Characterization of Engineered Tissues in the Canal Space of Closed-Apex Teeth with Apical Periodontitis. *J Endod* **2013**, *39*, 1549–1556, doi:10.1016/j.joen.2013.08.023.
324. Mohamed, D.A.-A.; Abdelwahab, S.A.; Mahmoud, R.H.; Taha, R.M. Radiographic and Immuno-Histochemical Evaluation of Root Perforation Repair Using MTA with or without Platelet-Rich Fibrin or Concentrated Growth Factors as an Internal Matrix in Dog's Teeth: In Vivo Animal Study. *Clin Oral Investig* **2023**, *27*, 5103–5119, doi:10.1007/s00784-023-05131-x.
325. Moradi, S.; Talati, A.; Forghani, M.; Jafarian, A.H.; Naseri, M.; Shojaeian, S. Immunohistological Evaluation of Revascularized Immature Permanent Necrotic Teeth Treated by Platelet-Rich Plasma: An Animal Investigation. *Cell J* **2016**, *18*, 389–396.
326. Moradi, S.; Saghravanian, N.; Moushekhian, S.; Fatemi, S.; Forghani, M. Immunohistochemical Evaluation of Fibronectin and Tenascin Following Direct Pulp Capping with Mineral Trioxide Aggregate, Platelet-Rich Plasma and Propolis in Dogs' Teeth. *Iran Endod J* **2015**, *10*, 188–192, doi:10.7508/IEJ.2015.03.009,.
327. Orhan, E.O.; Maden, M.; Senguüven, B. Odontoblast-like Cell Numbers and Reparative Dentine Thickness after Direct Pulp Capping with Platelet-Rich Plasma and Enamel Matrix Derivative: A Histomorphometric Evaluation. *Int Endod J* **2012**, *45*, 317–325, doi:10.1111/j.1365-2591.2011.01977.x.
328. Petrović, V.; Pejčić, N.; Čakić, S. The Influence of Different Therapeutic Modalities and Platelet Rich Plasma on Apexogenesis - A Preliminary Study in Monkeys. *Advances in Clinical and Experimental Medicine* **2013**, *22*, 469–479.
329. Puspita, S.; Utoro, T.; Haniastuti, T. Nestin Expressions of Exposed Pulp after Direct Pulp Capping by Calcium Hydroxide and Platelet Rich Plasma. *Eur J Dent* **2016**, *10*, 341–344, doi:10.4103/1305-7456.184157,.
330. Rodríguez-Benítez, S.; Stambolsky, C.; Gutiérrez-Pérez, J.L.; Torres-Lagares, D.; Segura-Egea, J.J. Pulp Revascularization of Immature Dog Teeth with Apical Periodontitis Using Triantibiotic Paste and Platelet-Rich Plasma: A Radiographic Study. *J Endod* **2015**, *41*, 1299–1304, doi:10.1016/j.joen.2015.05.002.
331. Stambolsky, C.; Rodríguez-Benítez, S.; Gutiérrez-Pérez, J.L.; Torres-Lagares, D.; Martín-González, J.; Segura-Egea, J.J. Histologic Characterization of Regenerated Tissues after Pulp Revascularization of Immature Dog Teeth with Apical Periodontitis Using Tri-Antibiotic Paste and Platelet-Rich Plasma. *Arch Oral Biol* **2016**, *71*, 122–128, doi:10.1016/j.archoralbio.2016.07.007.
332. Tabatabayi, M.H.; Tavakoli, A.; Ameghani, B.A. Regenerative Property of PRF Used as Capping Material in Pulpotomy in Dogs. *Biomedical Research (India)* **2017**, *28*, 4634–4639.
333. Tawfik, H.E.; Abu-Seida, A.M.; Hashem, A.A.; El-Khawlani, M.M. Treatment of Experimental Furcation Perforations with Mineral Trioxide Aggregate, Platelet Rich Plasma or Platelet Rich Fibrin in Dogs' Teeth. *Experimental and Toxicologic Pathology* **2016**, *68*, 321–327, doi:10.1016/j.etp.2016.03.004.
334. Torabinejad, M.; Milan, M.; Shabahang, S.; Wright, K.R.; Faras, H. Histologic Examination of Teeth with Necrotic Pulps and Periapical Lesions Treated with 2 Scaffolds: An Animal Investigation. *J Endod* **2015**, *41*, 846–852, doi:10.1016/j.joen.2015.01.026.
335. Torabinejad, M.; Faras, H.; Corr, R.; Wright, K.R.; Shabahang, S. Histologic Examinations of Teeth Treated with 2 Scaffolds: A Pilot Animal Investigation. *J Endod* **2014**, *40*, 515–520, doi:10.1016/j.joen.2013.12.025.
336. Petrovic, V.; Pejcic, N.; Rakic, M.; Lekovic, V.; Vasic, U.; Stojic, Z. Effects of the Platelet Rich Plasma on Apexogenesis in Young Monkeys: Radiological and Hystologycal Evaluation. *Acta Vet Brno* **2012**, *62*, 39–52, doi:10.2298/AVB1201039P.
337. Wang, Q.L.; Yang, P.P.; Ge, L.H.; Liu, H. Preliminary Evaluation of Platelet Rich Fibrin-Mediated Tissue Repair in Immature Canine Pulpless Teeth. *Chin J Dent Res* **2016**, *19*, 49–54, doi:10.3290/j.cjdr.a35697.

338. XI, X.; LIU, L.; CHEN, W.; HAN, G. Application of Platelet-Rich Plasma in Regenerated Pulp Treatment of Young Permanent Teeth, an Animal Study. *Indian J Pharm Sci* **2020**, *82*, 83–87, doi:10.36468/pharmaceutical-sciences.spl.90.
339. Yamada, M.; Nagayama, M.; Miyamoto, Y.; Kawano, S.; Takitani, Y.; Tanaka, M.; Ehara, M.; Nakao, J.; Ochiai, T.; Shibukawa, Y.; et al. Mineral Trioxide Aggregate (MTA) Upregulates the Expression of DMP1 in Direct Pulp Capping in the Rat Molar. *Materials 2021, Vol. 14, Page 4640* **2021**, *14*, 4640, doi:10.3390/MA14164640.
340. Yang, J.M.; Yang, K. Il; Lee, K.H.; Choi, S.H.; Kim, B.O.; Park, J.C.; Yu, S.J. Effects of Platelet-Rich Plasma on Tooth Replantation in Dogs: A Histologic and Histomorphometric Analysis. *J Periodontal Implant Sci* **2018**, *48*, 224–235, doi:10.5051/JPIS.2018.48.4.224.
341. Zaky, S.H.; AlQahtani, Q.; Chen, J.; Patil, A.; Taboas, J.; Beniash, E.; Ray, H.; Sfeir, C. Effect of the Periapical "Inflammatory Plug" on Dental Pulp Regeneration: A Histologic In Vivo Study. *J Endod* **2020**, *46*, 51–56, doi:10.1016/j.joen.2019.10.006.
342. Zhang, D.D.; Chen, X.; Bao, Z.F.; Chen, M.; Ding, Z.J.; Zhong, M. Histologic Comparison between Platelet-Rich Plasma and Blood Clot in Regenerative Endodontic Treatment: An Animal Study. *J Endod* **2014**, *40*, 1388–1393, doi:10.1016/j.joen.2014.03.020.
343. Zhou, R.; Wang, Y.; Chen, Y.; Chen, S.; Lyu, H.; Cai, Z.; Huang, X. Radiographic, Histologic, and Biomechanical Evaluation of Combined Application of Platelet-Rich Fibrin with Blood Clot in Regenerative Endodontics. *J Endod* **2017**, *43*, 2034–2040, doi:10.1016/j.joen.2017.07.021.
344. Zhu, W.; Zhu, X.; Huang, G.T.J.; Cheung, G.S.; Dissanayaka, W.L.; Zhang, C. Regeneration of Dental Pulp Tissue in Immature Teeth with Apical Periodontitis Using Platelet-Rich Plasma and Dental Pulp Cells. *Int Endod J* **2013**, *46*, 962–970, doi:10.1111/iej.12087.
345. Zhu, X.; Wang, Y.; Liu, Y.; Huang, G.T.J.; Zhang, C. Immunohistochemical and Histochemical Analysis of Newly Formed Tissues in Root Canal Space Transplanted with Dental Pulp Stem Cells plus Platelet-Rich Plasma. *J Endod* **2014**, *40*, 1573–1578, doi:10.1016/j.joen.2014.05.010.
346. Zhu, X.; Zhang, C.; Huang, G.T.J.; Cheung, G.S.P.; Dissanayaka, W.L.; Zhu, W. Transplantation of Dental Pulp Stem Cells and Platelet-Rich Plasma for Pulp Regeneration. *J Endod* **2012**, *38*, 1604–1609, doi:10.1016/j.joen.2012.09.001.
347. Vanja, P.; Vesna, D.; D., M.; S., C.; N., K.; Danica, M. The Effects of Hydroxyapatite and Platelet Rich Plasma on Apexogenesis in Monkeys. *Acta Vet Brno* **2009**, *59*, 277–289, doi:10.2298/AVB0903277P.
348. D. Prasanthi, NalamN. V; Simpsy, G.; Chittem, J.; Sajjan, G. Biological Approach in the Management of Permanent Molars with Irreversible Pulpitis Using Platelet-Rich Fibrin as a Pulpotomy Medicament: Case Reports with 2-Year Follow Up. *Journal of Interdisciplinary Dentistry* **2018**, *8*, 30, doi:10.4103/JID.JID_54_17.
349. Arora, A.; Bhesania, D.; Kapoor, S.; Kaur, H. A 5 Years' Follow-up of Root Anatomy-Based Maturogenesis Achieved in Infected Immature Molars Using Regenerative Techniques - A Case Series. *Journal of Conservative Dentistry* **2020**, *23*, 422, doi:10.4103/JCD.JCD_327_20.
350. Galagali, G. A Natural Meliorate: Revolutionary Tissue Engineering in Endodontics. *Journal of Clinic and Diagnostic Research* **2013**, *7*, 2644–2646, doi:10.7860/JCDR/2013/6915.3638.
351. Padhiary, S.K.; Pathak, H.M.; Subudhi, S.; Biswas, S. Apicocectomy with Placement of Prf in Periapical Lesions: Case Reports. *Indian J Public Health Res Dev* **2018**, *9*, 1127, doi:10.5958/0976-5506.2018.01606.6.
352. Singh, S.; Singh, A.; Singh, S.; Singh, R. Application of PRF in Surgical Management of Periapical Lesions. *Natl J Maxillofac Surg* **2013**, *4*, 94, doi:10.4103/0975-5950.117825.
353. Uppada, U.K.; Kalakonda, B.; Koppolu, P.; varma, N.; Palakurthy, K.; Manchikanti, V.; Prasad, S.; Samar, S.; Swapna, L.A. Combination of Hydroxyapatite, Platelet Rich Fibrin and Amnion Membrane as a Novel Therapeutic Option in Regenerative Periapical Endodontic Surgery: Case Series. *Int J Surg Case Rep* **2017**, *37*, 139–144, doi:10.1016/j.ijscr.2017.06.009.
354. Biradar, N.; Ragulakollu, R.; Bogishetty, C.; Tej, G.; Gandham, S.; Vardhan, P. Combination Therapy of Antibiotics and Platelet-Rich Fibrin for Apical Closure: Case Series. *Int J Clin Pediatr Dent* **2023**, *16*, 541–546, doi:10.5005/jp-journals-10005-2613.

355. Shah, N.; Logani, A.; Jadhav, G. Comparative Outcome of Revascularization in Bilateral, Non-Vital, Immature Maxillary Anterior Teeth Supplemented with or without Platelet Rich Plasma: A Case Series. *Journal of Conservative Dentistry* **2013**, *16*, 568, doi:10.4103/0972-0707.120932.
356. Bezgin, T.; Yılmaz, A.D.; Çelik, B.N.; Sönmez, H. Concentrated Platelet-Rich Plasma Used in Root Canal Revascularization: 2 Case Reports. *Int Endod J* **2014**, *47*, 41–49, doi:10.1111/iej.12144.
357. Shetty, S.; Suneetha, M.; Mittal, S.; Krishna Vallabhaneni, S.S.; Dande, S.; Kothari, S.; Tiwari, H. Coronal Pulpotomy Technique Analysis as an Alternative to Pulpectomy for Preserving the Tooth Vitality in Context to Tissue Regeneration: A Correlated Clinical Study across 4 Permanent Molars. *European Journal of Molecular and Clinical Medicine* **2020**, *7*, 5255–5262.
358. Solomon, R.V.; Faizuddin, U.; Karunakar, P.; Deepthi Sarvani, G.; Sree Soumya, S. Coronal Pulpotomy Technique Analysis as an Alternative to Pulpectomy for Preserving the Tooth Vitality, in the Context of Tissue Regeneration: A Correlated Clinical Study across 4 Adult Permanent Molars. *Case Rep Dent* **2015**, *2015*, 1–12, doi:10.1155/2015/916060.
359. Sharma, V.; Sharma, S.; Dudeja, P.; Grover, S. Endodontic Management of Nonvital Permanent Teeth Having Immature Roots with One Step Apexification, Using Mineral Trioxide Aggregate Apical Plug and Autogenous Platelet-Rich Fibrin Membrane as an Internal Matrix: Case Series. *Contemp Clin Dent* **2016**, *7*, 67–70, doi:10.4103/0976-237X.177107.
360. Sharma, S.; Sharma, V.; Passi, D.; Srivastava, D.; Grover, S.; Dutta, S.R. Large Periapical or Cystic Lesions in Association with Roots Having Open Apices Managed Nonsurgically Using 1-Step Apexification Based on Platelet-Rich Fibrin Matrix and Biodentine Apical Barrier: A Case Series. *J Endod* **2018**, *44*, 179–185, doi:10.1016/j.joen.2017.08.036.
361. Pires, M.D.; Martins, J.N.R.; Baruwa, A.O.; Pereira, B.; Ginjeira, A. Leukocyte Platelet-Rich Fibrin in Endodontic Microsurgery: A Report of 2 Cases. *Restor Dent Endod* **2022**, *47*, e17–e17, doi:10.5395/rde.2022.47.e17.
362. Nawal, R.R.; Utneja, S.; Sharma, V.; Yadav, S.; Talwar, S. Long-Term Follow-up of Traumatized Immature Necrotic Permanent Teeth Treated with Regenerative Endodontic Protocol Using Platelet-Rich Fibrin: A Prospective Case Series. *J Conserv Dent* **2020**, *23*, 417–421, doi:10.4103/JCD.JCD_460_20.
363. Chen, Y.; Lv, H.; Lei, L.; Zhou, R.; Huang, X. Management of Dens Evaginatus with an Immature Permanent Tooth with Apical Periodontitis Using Platelet-Rich Fibrin in Regenerative Endodontics: Case Reports and Literature Review. *Int J Clin Exp Med* **2017**, *10*, 3801–3808.
364. E, D. Management of Large Preiapical Lesion with the Combination of Second Generation Platelet Extract and Hydroxyapatite Bone Graft: A Report of Three Cases. *Journal of Clinic and Diagnostic Research* **2015**, *9*, ZD24–ZD27, doi:10.7860/JCDR/2015/10885.5482.
365. Zhao, J.H.; Tsai, C.H.; Chang, Y.C. Management of Radicular Cysts Using Platelet-Rich Fibrin and Bioactive Glass: A Report of Two Cases. *Journal of the Formosan Medical Association* **2014**, *113*, 470–476, doi:10.1016/j.jfma.2011.09.027.
366. Pruthi, P.J.; Goel, S.; Yadav, P.; Nawal, R.R.; Talwar, S. Novel Application of a Calcium Silicate–based Cement and Platelet-Rich Fibrin in Complex Endodontic Cases: A Case Series. *Gen Dent* **2020**, *68*, 46–49.
367. Bakhtiar, H.; Esmaeili, S.; Fakhr Tabatabayi, S.; Ellini, M.R.; Nekoofar, M.H.; Dummer, P.M. Second-Generation Platelet Concentrate (Platelet-Rich Fibrin) as a Scaffold in Regenerative Endodontics: A Case Series. *J Endod* **2017**, *43*, 401–408, doi:10.1016/j.joen.2016.10.016.
368. Gaviño Orduña, J.F.; Caviedes-Bucheli, J.; Manzanares Céspedes, M.C.; Berástegui Jimeno, E.; Martín Biedma, B.; Segura-Egea, J.J.; López-López, J. Use of Platelet-Rich Plasma in Endodontic Procedures in Adults: Regeneration or Repair? A Report of 3 Cases with 5 Years of Follow-Up. *J Endod* **2017**, *43*, 1294–1301, doi:10.1016/j.joen.2017.04.010.
369. Kandemir Demirci, G.; Güneri, P.; Çalışkan, M.K. Regenerative Endodontic Therapy with Platelet Rich Fibrin: Case Series. *J Clin Pediatr Dent* **2020**, *44*, 15–19, doi:10.17796/1053-4625-44.1.3.
370. Karunakar, P.; Prasanna, J.; Jayadev, M.; Shravani, G. Platelet-Rich Fibrin, "a Faster Healing Aid" in the Treatment of Combined Lesions: A Report of Two Cases. *J Indian Soc Periodontol* **2014**, *18*, 651–655, doi:10.4103/0972-124X.142467,.

371. Lee, K.-Y.; Lee, S.-H.; Lee, N.-Y. Vital Pulp Therapy Using Platelet-Rich Fibrin in an Immature Permanent Tooth: Case Reports. *THE JOURNAL OF THE KOREAN ACADEMY OF PEDTATRIC DENTISTRY* **2013**, *40*, 120–126, doi:10.5933/JKAPD.2013.40.2.120.
372. Topçuoğlu, G.; Topçuoğlu, H.S. Regenerative Endodontic Therapy in a Single Visit Using Platelet-Rich Plasma and Biodentine in Necrotic and Asymptomatic Immature Molar Teeth: A Report of 3 Cases. *J Endod* **2016**, *42*, 1344–1346, doi:10.1016/j.joen.2016.06.005.
373. Yang, Y.; Liu, Y.-L.; Jia, L.-N.; Wang, J.-J.; Zhang, M. Rescuing "Hopeless" Avulsed Teeth Using Autologous Platelet-Rich Fibrin Following Delayed Reimplantation: Two Case Reports. *World J Clin Cases* **2023**, *11*, 635–644, doi:10.12998/wjcc.v11.i3.635.
374. Yoshpe, M.; Kaufman, A.Y.; Lin, S.; Ashkenazi, M. Regenerative Endodontics: A Promising Tool to Promote Periapical Healing and Root Maturation of Necrotic Immature Permanent Molars with Apical Periodontitis Using Platelet-Rich Fibrin (PRF). *Eur Arch Paediatr Dent* **2021**, *22*, 527–534, doi:10.1007/s40368-020-00572-4.
375. Hong, S.; Chen, W.; Jiang, B. A Comparative Evaluation of Concentrated Growth Factor and Platelet-Rich Fibrin on the Proliferation, Migration, and Differentiation of Human Stem Cells of the Apical Papilla. *J Endod* **2018**, *44*, 977–983, doi:10.1016/j.joen.2018.03.006.
376. Panda, P.; Govind, S.; Sahoo, S.K.; Pattanaik, S.; Mallikarjuna, R.M.; Nalawade, T.; Saraf, S.; Khaldi, N. Al; Jahdhami, S. Al; Shivagange, V.; et al. Analysis of Pulp Tissue Viability and Cytotoxicity of Pulp Capping Agents. *J Clin Med* **2023**, *12*, 539, doi:10.3390/jcm12020539.
377. Singh, P.; Dey, S.; Pandey, V.; Abhas, A.; Sharan, S.; Kharat, N. Antibacterial and Antifungal Efficacy of Platelet-Rich Fibrin and Platelet-Rich Fibrin Matrix against Root Canal Microflora. *J Pharm Bioallied Sci* **2021**, *13*, S124–S127, doi:10.4103/jpbs.JPBS_601_20.
378. Woo, S.-M.; Kim, W.-J.; Lim, H.-S.; Choi, N.-K.; Kim, S.-H.; Kim, S.-M.; Jung, J.-Y. Combination of Mineral Trioxide Aggregate and Platelet-Rich Fibrin Promotes the Odontoblastic Differentiation and Mineralization of Human Dental Pulp Cells via BMP/Smad Signaling Pathway. *J Endod* **2016**, *42*, 82–88, doi:10.1016/j.joen.2015.06.019.
379. Gomez, T.W.; Gopal, R. V.; Gaffoor, F.M.A.; Kumar, S.T.R.; Sabari Girish, C.; Prakash, R. Comparative Evaluation of Angiogenesis Using a Novel Platelet-Rich Product: An in Vitro Study. *Journal of Conservative Dentistry* **2019**, *22*, 23–27, doi:10.4103/JCD.JCD_216_18,.
380. Khatri, S.; Mathew, S.; Nagaraja, S.; Hegde, S.; Ghosh, S.; Ravichandran, K. Comparative Evaluation of PH and Ca+ Ion Release from MTA on Interaction with Platelet-Rich Fibrin and Blood Clot: An in Vitro Study. *F1000Res* **2023**, *12*, 364, doi:10.12688/f1000research.130227.2.
381. Girija, K.; Kavitha, M. Comparative Evaluation of Platelet-Rich Fibrin, Platelet-Rich Fibrin + 50 Wt% Nanohydroxyapatite, Platelet-Rich Fibrin + 50 Wt% Dentin Chips on Odontoblastic Differentiation - An in Vitro Study-Part 2. *Journal of Conservative Dentistry* **2020**, *23*, 354, doi:10.4103/JCD.JCD_3_20.
382. Mahendran, K.; Kottuppallil, G.; Sekar, V. Comparative Evaluation of Radiopacity and Cytotoxicity of Platelet-Rich Fibrin, Platelet-Rich Fibrin + 50wt% Nano-Hydroxyapatite, Platelet-Rich Fibrin + 50wt% Dentin Chips: An in Vitro Study. *J Conserv Dent* **2019**, *22*, 28–33, doi:10.4103/jcd.jcd_281_18.
383. Duan, J.; Kuang, W.; Tan, J.; Li, H.; Zhang, Y.; Hirotaka, K.; Tadashi, K. Differential Effects of Platelet Rich Plasma and Washed Platelets on the Proliferation of Mouse MSC Cells. *Mol Biol Rep* **2011**, *38*, 2485–2490, doi:10.1007/s11033-010-0385-7.
384. Dou, L.; Yan, Q.; Yang, D. Effect of Five Dental Pulp Capping Agents on Cell Proliferation, Viability, Apoptosis and Mineralization of Human Dental Pulp Cells. *Exp Ther Med* **2020**, *19*, 2377–2383, doi:10.3892/etm.2020.8444.
385. Kim, J.H.; Woo, S.M.; Choi, N.K.; Kim, W.J.; Kim, S.M.; Jung, J.Y. Effect of Platelet-Rich Fibrin on Odontoblastic Differentiation in Human Dental Pulp Cells Exposed to Lipopolysaccharide. *J Endod* **2017**, *43*, 433–438, doi:10.1016/j.joen.2016.11.002.
386. Li, X.; Hou, J.; Wu, B.; Chen, T.; Luo, A. Effects of Platelet-Rich Plasma and Cell Coculture on Angiogenesis in Human Dental Pulp Stem Cells and Endothelial Progenitor Cells. *J Endod* **2014**, *40*, 1810–1814, doi:10.1016/j.joen.2014.07.022.
387. Chhaya, D.; Vaidya, N.; Patel, V.; Chudasama, K.; Doshi, S.; Kumar, P. Evaluation and Comparison of Mechanical Properties of Platelet-Rich Fibrin Membrane, Fish Collagen Membrane, Bovine Collagen Membrane

and Chorionic Membrane – An SEM Study. *Indian Journal of Dental Research* **2022**, *33*, 425, doi:10.4103/ijdr.ijdr_556_22.

388. Nagaraja, S.; Mathew, S.; Rajaram, R.; Pushpalatha, C.; Abraham, A.; Chandanala, S. Evaluation of Histological and PH Changes in Platelet-Rich Fibrin and Platelet-Rich Fibrin Matrix: A In Vitro Study. *Contemp Clin Dent* **2019**, *10*, 652, doi:10.4103/ccd.ccd_10_19.
389. Nagaraja, S.; Mathew, S.; Abraham, A.; Ramesh, P.; Chandanala, S. Evaluation of Vascular Endothelial Growth Factor - A Release from Platelet-Rich Fibrin, Platelet-Rich Fibrin Matrix, and Dental Pulp at Different Time Intervals. *Journal of Conservative Dentistry* **2020**, *23*, 359–363, doi:10.4103/JCD.JCD_465_19,.
390. Bi, J.; Liu, Y.; Liu, X.M.; Lei, S.; Chen, X. Platelet-Rich Fibrin Improves the Osteo-/Odontogenic Differentiation of Stem Cells from Apical Papilla via the Extracellular Signal-Regulated Protein Kinase Signaling Pathway. *J Endod* **2020**, *46*, 648–654, doi:10.1016/j.joen.2020.02.004.
391. Huang, F.M.; Yang, S.F.; Zhao, J.H.; Chang, Y.C. Platelet-Rich Fibrin Increases Proliferation and Differentiation of Human Dental Pulp Cells. *J Endod* **2010**, *36*, 1628–1632, doi:10.1016/j.joen.2010.07.004.
392. Altaii, M.; Kaidonis, X.; Koblar, S.; Cathro, P.; Richards, L. Platelet Rich Plasma and Dentine Effect on Sheep Dental Pulp Cells Regeneration/Revitalization Ability (in Vitro). *Aust Dent J* **2017**, *62*, 39–46, doi:10.1111/adj.12426.
393. Yeom, K.H.; Ariyoshi, W.; Okinaga, T.; Washio, A.; Morotomi, T.; Kitamura, C.; Nishihara, T. Platelet-rich Plasma Enhances the Differentiation of Dental Pulp Progenitor Cells into Odontoblasts. *Int Endod J* **2016**, *49*, 271–278, doi:10.1111/iej.12443.
394. Xu, H.; Xu, F.; Zhao, J.; Zhou, C.; Liu, J.; Hwa Lee, R.; El-Fiqi, A.; Austin Gregory, C. Platelet-Rich Plasma Induces Autophagy and Promotes Regeneration in Human Dental Pulp Cells. *Front Bioeng Biotechnol* **2021**, *9*, 659742, doi:10.3389/FBIOE.2021.659742.
395. Mullaguri, H.; Suresh, N.; Surendran, S.; Velmurugan, N.; Chitra, S. Role of PH Changes on Transforming Growth Factor-B1 Release and on the Fibrin Architecture of Platelet-Rich Fibrin When Layered with Biodentine, Glass Ionomer Cement, and Intermediate Restorative Material. *J Endod* **2016**, *42*, 766–770, doi:10.1016/j.joen.2016.02.009.
396. Nagaraja, S.; Mathew, S.; Jain, N.; Jethani, B.; Nambiar, S.; Kumari, M.; Nair, S. Study of Antibacterial and Antifungal Efficacy of Platelet-Rich Fibrin and Platelet-Rich Fibrin Matrix. *Journal of Conservative Dentistry* **2019**, *22*, 415–419, doi:10.4103/JCD.JCD_100_19,.
397. Chai, J.; Jin, R.; Yuan, G.; Kanter, V.; Miron, R.J.; Zhang, Y. Effect of Liquid Platelet-Rich Fibrin and Platelet-Rich Plasma on the Regenerative Potential of Dental Pulp Cells Cultured under Inflammatory Conditions: A Comparative Analysis. *J Endod* **2019**, *45*, 1000–1008, doi:10.1016/j.joen.2019.04.002.
398. Kritika, S.; Sujatha, V.; Srinivasan, N.; Renganathan, S.K.; Mahalaxmi, S. Prospective Cohort Study of Regenerative Potential of Non Vital Immature Permanent Maxillary Central Incisors Using Platelet Rich Fibrin Scaffold. *Sci Rep* **2021**, *11*, 13679, doi:10.1038/s41598-021-93236-2.
399. Thirumagal, K.; Pradeep, S. Retrospective Analysis on Type of Scaffolds Used in Revascularisation-A Institutional Based Study. *J Res Med Dent Sci* **2022**, *10*, 176–180.
400. Lv, H.; Chen, Y.; Cai, Z.; Lei, L.; Zhang, M.; Zhou, R.; Huang, X. The Efficacy of Platelet-Rich Fibrin as a Scaffold in Regenerative Endodontic Treatment: A Retrospective Controlled Cohort Study. *BMC Oral Health* **2018**, *18*, 1–8, doi:10.1186/S12903-018-0598-Z/TABLES/2.
401. Sahito, A.H.; Kuhuawar, S.R.; Jokhio, A.L.; Tagar, M.R.; Shaikh, M.A.; Kalwar, M.R. To Determine Clinical Outcome of Platelet Rich Fibrin in Pulpotomy of Permanent Teeth in Irreversible Pulpitis. *Journal of the Liaquat University of Medical and Health Sciences* **2022**, *21*, 117–120, doi:10.22442/jlumhs.2021.00823.
402. Yoshpe, M.; Ruparel, N.; Einy, S.; Ganatra, S.; Kaufman, A.Y. Treatment of Necrotic Anterior and Posterior Teeth with Regenerative Endodontic Procedures Using PRF as a Scaffold: A Retrospective Study. *Applied Sciences* **2022**, *12*, 6774, doi:10.3390/app12136774.
403. Thakkar, S.; Naik, S.; Nadig, B.; Bellal, S.; Thaliyil, A. A Comparative Clinico-Radiographic Analysis of Regenerative Endodontic Procedure on Immature Necrotic Permanent Teeth Using Blood Clot and PRF as Scaffold: A Retrospective Study. *Saudi Dent J* **2023**, *35*, 753–759, doi:10.1016/j.sdentj.2023.05.026.

404. Li, J.; Zheng, L.; Daraqel, B.; Liu, J.; Hu, Y. The Efficacy of Concentrated Growth Factor and Platelet-Rich Fibrin as Scaffolds in Regenerative Endodontic Treatment Applied to Immature Permanent Teeth: A Retrospective Study. *BMC Oral Health* **2023**, *23*, 482, doi:10.1186/s12903-023-03164-y.
405. Singh, R.; Kavita, K.; Kommula, A.; Kulkarni, G.; Jois, H.S. To Compare Mineral Trioxide Aggregate, Platelet-Rich Fibrin, and Calcium Hydroxide in Teeth with Irreversible Pulpitis: A Clinical Study. *J Pharm Bioallied Sci* **2020**, *12*, S436-S439.
406. Conde, M.C.M.; Chisini, L.A.; Sarkis-Onofre, R.; Schuch, H.S.; Nör, J.E.; Demarco, F.F. A Scoping Review of Root Canal Revascularization: Relevant Aspects for Clinical Success and Tissue Formation. *Int Endod J* **2017**, *50*, 860–874, doi:10.1111/iej.12711.
407. Chisini, L.A.; Conde, M.C.M.; Grazioli, G.; Martin, A.S.S.; Carvalho, R.V. de; Sartori, L.R.M.; Demarco, F.F. Bone, Periodontal and Dental Pulp Regeneration in Dentistry: A Systematic Scoping Review. *Braz Dent J* **2019**, *30*, 77–95, doi:10.1590/0103-6440201902053.
408. Ardila, C.M.; Vivares-Builes, A.M. Clinical Efficacy of Treatment of Endodontic-Periodontal Lesions: A Systematic Scoping Review of Experimental Studies. *Int J Environ Res Public Health* **2022**, *19*, 13649, doi:10.3390/ijerph192013649.
409. Caviedes-Bucheli, J.; Muñoz-Alvear, H.D.; Lopez-Moncayo, L.F.; Narvaez-Hidalgo, A.; Zambrano-Guerrero, L.; Gaviño-Orduña, J.F.; Portigliatti, R.; Gomez-Sosa, J.F.; Munoz, H.R. Use of Scaffolds and Regenerative Materials for the Treatment of Immature Necrotic Permanent Teeth with Periapical Lesion: Umbrella Review. *Int Endod J* **2022**, *55*, 967–988, doi:10.1111/iej.13799.
410. Lopes, L.; Neves, J.A.; Botelho, J.; Machado, V.; Mendes, J.J. Regenerative Endodontics Procedure: An Umbrella Review. *Int J Environ Res Public Health* **2021**, *18*, 1–17, doi:10.3390/IJERPH18020754,.
411. Ctri Comparasion of Success Rate of Pulpotomy with Biodentine Using PRF Membrane and Collagen Scaffold in Permanent Molars. *https://trialsearch.who.int/Trial2.aspx?TrialID=CTRI/2023/03/050834* **2023**.
412. Li, J.; Zheng, L.; Daraqel, B.; Liu, J.; Hu, Y. The Efficacy of Concentrated Growth Factor and Platelet-Rich Fibrin as Scaffolds in Regenerative Endodontic Treatment Applied to Immature Permanent Teeth: A Retrospective Study. *BMC Oral Health* **2023**, *23*, 482, doi:10.1186/s12903-023-03164-y.
